# Coral metabolome quality and contaminant loads track human land use

**DOI:** 10.1038/s41467-026-74960-7

**Published:** 2026-07-15

**Authors:** Zachary A. Quinlan, Austin Greene, William Leggat, Tess Moriarty, Tracy D. Ainsworth, Brunda Nijagal, Kim Falinski, E. Maggie Sogin, Jamie M. Caldwell, Scott F. Heron, Megan J. Donahue

**Affiliations:** 1 University of Hawai’i at Mānoa, Honolulu, HI USA; 2 Hawai’i Institute of Marine Biology, Kāne’ohe, HI USA; 3 Woods Hole Oceanographic Institution, Woods Hole, MA USA; 4 University of Newcastle, Ourimbah, NSW Australia; 5 University of New South Wales, Sydney, NSW Australia; 6 Metabolomics Australia, Bio21 Molecular Science and Biotechnology Institute, Parkville, VIC Australia; 7 The Nature Conservancy, Honolulu, HI USA; 8 Molecular Cell Biology, University of California Merced, Merced, CA USA; 9 High Meadows Environmental Institute, Princeton University, Princeton, NJ USA; 10 Physics and Marine Geophysical Laboratory, James Cook University, Townsville, QLD Australia

**Keywords:** Conservation biology, Marine chemistry, Chemical ecology, Metabolomics

## Abstract

Anthropogenic activities pose a significant risk to vulnerable marine ecosystems like coral reefs, particularly those near human population centers. Despite coral reefs being one of the systems most at risk, the relationship between contaminant exposure and coral health across gradients of ecosystem disturbance remains poorly understood. Our metabolomic analysis of 380 corals from 16 sites spanning an anthropogenic impact gradient reveals that the coral metabolome is predictive of anthropogenic ecosystem disturbance. We found that human activities both within catchments and in the marine ecosystem altered the chemodiversity of coral metabolomes with a direct relationship to historic trends in coral cover: nitrogen reserves and metabolome energetic potential are reduced, while stress metabolites are enriched at more impacted sites. We further identify 25 anthropogenic contaminants from agricultural, cosmetological, industrial, and pharmaceutical sources accumulated in coral metabolomes. Our results suggest that the bioaccumulation of pollutants and compositional changes in the metabolome of corals imposed by anthropogenic activities is correlated with coral benthic cover. Together, our findings suggest a direct relationship between coral health, accumulation of dangerous contaminants within corals, and anthropogenic disturbance that is consistent across species.

## Abstract (web summary)

The proximity of corals to human populations and nearshore activities exposes them to physical and chemical disturbances. This study suggests a direct relationship between coral health, the accumulation of harmful contaminants, and anthropogenic disturbance, consistent across species.

## Introduction

Reef-building corals, which foster the most biodiverse marine ecosystems^1,2^, are declining rapidly as a result of human activities^3^. The direct impact of near-shore human populations and associated development, agriculture, urbanization, and waste accumulation have resulted in reduced coastal water quality, reef community phase shifts, and decreased coral physiological performance^4–7^. A wide breadth of pollutants are associated with agricultural runoff, groundwater, and wastewater contamination. The introduction of pollutants via these human activity pathways to marine ecosystems poses a substantial threat to coral reef ecosystems^8^.

Given the rapid development of terrestrial landscapes and coastal catchments adjacent to coral reefs, there is an urgent need to quantify the accumulation of anthropogenic contaminants within the tissues of coral. Chronic pollutant exposure impacts coral development and function across life stages^9,10^, however, we do not know how chronic exposure to human activities modifies the metabolome potential of corals. Like other organisms, the coral metabolome contains an array of small molecules that reflect metabolic activity, physiological status, and exposure to pollutants^11–15^. Leveraging untargeted metabolomics in corals has greatly expanded our understanding of reef microbial structuring^16^, identified internal biomarkers of thermal stress^13^, and isolated pools of metabolites responsible for cueing coral larval settlement^17,18^. Building on these advancements, we now have the capacity to identify the direct impacts of human activities and anthropogenic chemical accumulation on coral metabolic potential.

In this study, we evaluate the metabolome composition of *Porites lobata* and *Montipora capitata* metabolomes at 16 sites along 70 km of coastline in Maui, Hawaiʻi, USA. Both species’ metabolome composition and chemodiversities are related to terrestrial and catchment ecosystem disturbance. Specifically, coral metabolomes that are depleted in nitrogen at higher-impacted sites, correspond to an enrichment of less energetically available structures. Corals in areas with high anthropogenic influence additionally accumulate an array of contaminants and stress metabolites. Historic coral cover data demonstrates that sites with higher contaminant loads, and lower energetic reserves experienced the steepest declines in coral cover since the 2015/2016 global bleaching event. These results suggest that corals use a portion of their energy reserves to respond to the stress levels imposed by local human activities, such as land-derived pollution, reducing their resilience by depleting the ability of these corals to respond to global stressors, such as increased sea surface temperature.

## Results

### Coral metabolome composition reflects terrestrial ecosystem disturbance

**Fig. 1:**
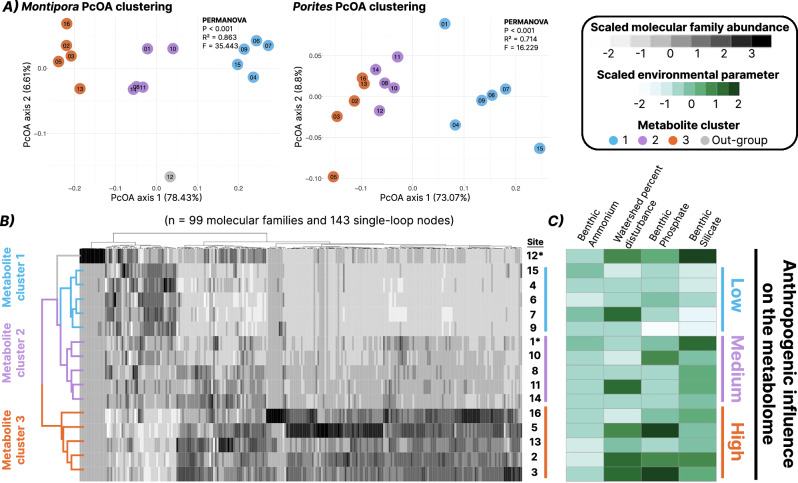
Terrestrial and catchment disturbance explain tissue metabolome diversity. **A** Principal coordinates analysis (PcOA) of the clustering of sites for both *M. capitata* and *P. lobata* tissue molecular family abundance. PcOA’s were calculated from Bray-Curtis distances of the site average molecular family abundance. Significant differences between clusters were tested using PERMANOVAs (degrees of freedom = 2). **B** Hierarchical clustering of metabolite abundance in *M. captiata* grouped sites into three distinct clusters. Metabolite relative abundance was summed within molecular families for each sample and averaged for each site. Cells are shaded by z-scored relative abundance of each molecular family. Clustering was performed using Euclidian distances. Asterisks next to site numbers indicate sites that change site clusters in *P. lobata* tissues (Supplementary Fig. 1). **C** Environmental parameters which significantly explained the variance in the metabolome chemodiversity. Benthic nutrients and onshore habitat percent disturbance are grouped and colored by the metabolite clusters from the hierarchical clustering. Each metabolite cluster is classified as less (blue), moderately (purple) or highly (orange) influenced by human activities.

Between March 12 and 23rd, 2018, we sampled 186 *Montipora capitata* colonies and 193 *Porites lobata* colonies (mean colony diameter, x̄ = 26 cm) at 16 reef sites across a spectrum of disturbance and land-use activities (mean depth, x̄ = 4 m) for untargeted metabolomic analysis. In each species, we identified 554 and 733 ion features, respectively (herein called metabolites) that varied in relative abundance between the sampling sites (linear mixed model FDR corrected *p* value < 0.05, full statistical results are available in the Supplementary Data 1). Principal coordinate analysis (Fig. 1A) and hierarchical clustering (Fig. 1B; Supplementary Fig. 1) of sites by mean molecular family relative abundance in coral tissue samples demonstrated consistent metabolome clustering in both coral species (Supplementary Fig. 2) corresponding with land use disturbance levels (*n* = 10 to 15 corals per site). The metabolome clustering was consistent whether dendrograms were built using molecular families that differed between sites or not (Supplementary Fig. 3). Importantly, these metabolome clusters were not defined *apriori* but were rather uncovered based on the separation of the sites metabolomic profiles in multidimensional space. For both coral species the metabolome clusters were significantly separated in multidimensional space (PERMANOVA p < 0.001; PERMANOVA R^2^ = 0.863 in *Montipora* and 0.714 in *Porites*; pairwise PERMANOVA < 0.01). There was no significant difference in dispersion within each metabolome cluster in either coral species (*p* > 0.05). Our results reflect land use gradients on Maui, which impact marine environments through connectivity of watersheds. For example, anthropogenic impacts to coastal waters includes disturbance induced by coastal development^19^, runoff of agricultural pesticide and nutrient pollution^10,20,21^, heavy tourism^22^, and the introduction of wastewater effluent and septage onto coral reefs via submarine groundwater discharge^23,24^.

**Fig. 2:**
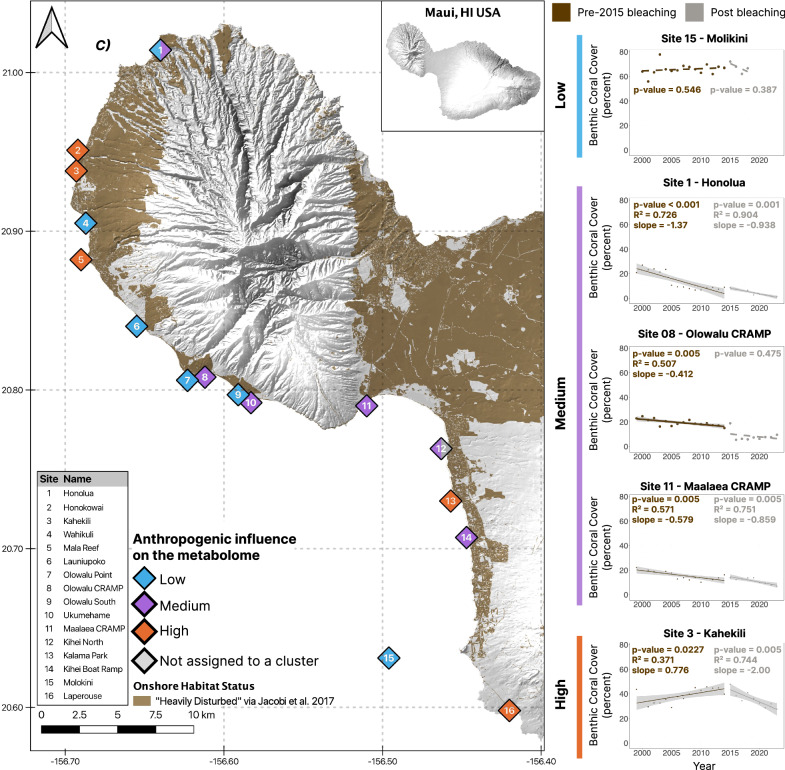
Historic trends in coral cover suggest that the most anthropogenically influenced sites experienced more severe responses to widespread bleaching events. Left) Map of sampling locations with sites colored by the relative amount of anthropogenic influence on coral metabolomes. Each site number is colored based on the metabolite cluster ecosystem disturbance. The two sites with different clusterings for *Porites/Montipora* (1, 12) have two colors to indicate their different cluster alignment. Right) The five sites from our metabolomic survey that had more than 19 years of coral cover survey data. Solid lines signify significant changes in coral cover over time defined from FDR-corrected linear models (full statistical reports are available in the Supplementary Data 1). Deviation bands represent the 95% confidence interval. Models were split into pre-2015 bleaching (brown) and post bleaching responses (gray) in coral cover.

Watershed disturbance and nutrient concentrations were correlated to variation in metabolome chemodiversity (linear model *p* value ≤ 0.017, df = 12; Fig. 1B). Watershed disturbance was inferred from satellite data^25^ wherein the proportional area of “Heavily Disturbed” habitat was calculated for a 3 km circular buffer immediately onshore of each coral sampling location. Hierarchical cluster membership explained more of the variability in multidimensional chemidiversity than the environmental disturbance metrics we collected for both *Montipora capitata* and *Porites lobata* (adjusted R^2^ = 97.17% and 84.84%, respectively, linear model *p* value < 0.001, df = 12). However, the continuous gradients of watershed disturbance, benthic ammonium, and benthic silicate disturbance still explained more than 50% of the chemodiversity variance at each site in both species (linear model *p* value ≤ 0.017; df = 12), with phosphate concentration explaining an additional 6.74% of the multidimensional chemodiversity variance in *Montipora capitata* (Supplementary Figs. 2 and 4). This suggests that metabolome chemodiversity and cluster membership was driven largely by ecosystem impacts. In fact, every site maintained the same cluster membership in both coral species except for Sites 1 and 12 (Supplementary Figs. 1,3,5). In *Montipora* tissues there was a single cluster of molecular families that were enriched in site 12. We investigated whether removal of these molecular families in *Montipora* tissues would change the clustering of sites. Indeed, once removed sites 12 and 1 clustered the exact same as in *Porites* tissues (Supplementary Fig 5). The close correspondence of watershed disturbance, water quality metrics, and cluster patterns in metabolite composition allowed us to assign sites into three groups based on the amount of influence that anthropogenic activities/nutrients had on the metabolome chemodiversity. We categorized these groups based on the data in terms of levels of metabolome disturbance (Fig. 2): low (cluster group 1), medium (cluster group 2) and to high (cluster group 3). Five sites had 19–25 years of benthic coral cover data from the Coral Reef Assessment and Monitoring Program (CRAMP)^26–28^. In 2015–2016, the Hawaiian reefs were part of the third global coral bleaching event because of a strong El Niño^29^. This survey data showed that the least resilient sites also had the most anthropogenically impacted metabolomes (Fig. 2). Prior to the 2015 bleaching event the only site where coral cover significantly increased was the site that had the most metabolome influence (Site 3: Kahekili). This increase can be largely attributed to the establishment of the Kahekili Herbivore Fisheries Management Area which sought to increase herbivorous fishes and contend with algal overgrowth at this location^30^. Since 2015, the steepest decline in coral cover was observed at the highly anthropogenically impacted Site 3 (Kahekili), followed by the moderately anthropogenically influenced Sites 1 (Honolua) and 11 (Māʻalaea) while the least influenced Site 15 (Molikini) and moderately influenced Site 8 (Olowalu) did not change significantly with time since 2015 (Fig. 2). In the case of Site 3, the Kahekili Herbivore Fisheries Management Area is still in effect, so the post-bleaching decline is in spite of these management efforts.

Sites belonging to the least influenced reef sites (site cluster 1) include those along southwest facing shores composed of agricultural lands (active or fallow), low development, and beachside highways (Sites 4, 6, 7, 9) with two sites having high mixing (Sites 6, 7), and one offshore site (Site 15; Fig. 2). Sites belonging to cluster group 2 include regions located next to a golf course, downstream of a sugar plantation (Sites 10, 11), areas with a high density of onsite wastewater systems and small wastewater treatment plants (Sites 8, 11), and sites downstream of former sugar plantation runoff (Sites 10, 11), and commercial tourism districts (Sites 12, 14). Finally, the most influenced sites (site cluster 3) include those located near wastewater treatment plants (sites 2, 3, 13), residential stream runoff outlet (Site 5), and regions using onsite waste disposal systems (Site 16).

### Greater ecosystem disturbance reduces coral energetic reserves and enriches contaminants

**Fig. 3:**
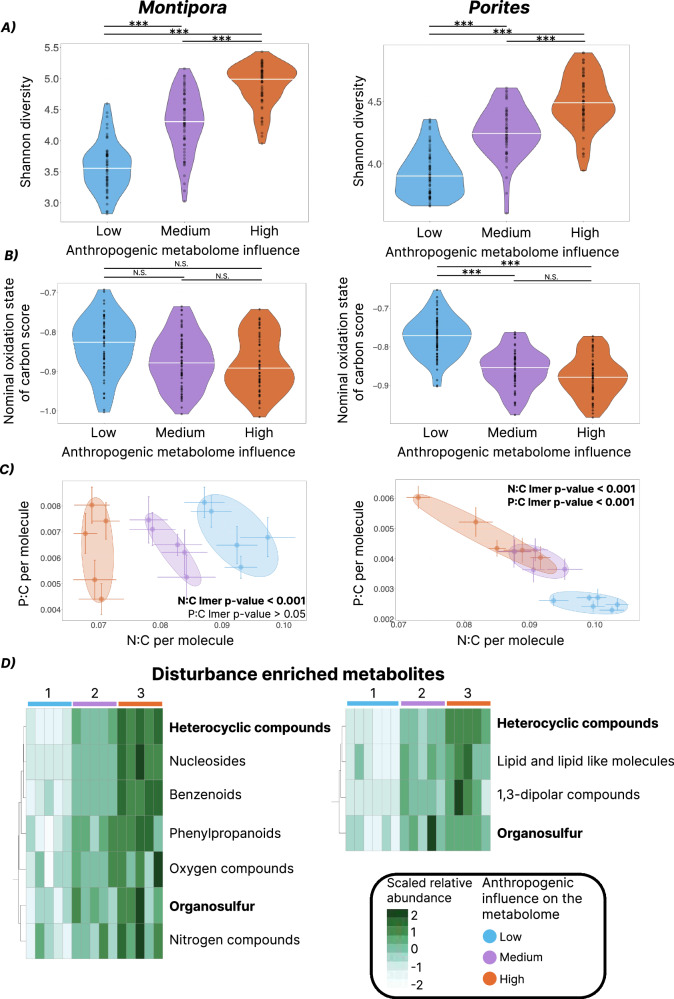
Metabolome chemodiversity, oxidation state, and nutrient content is disrupted by ecosystem disturbance. **A** Shannon diversity of metabolomes across sites. **B** Weighted nominal oxidation state of carbon (NOSC) for each sample. **C** Average site elemental ratios of P:C versus N:C weighted by metabolite relative abundance. Each point represents a single sampling site with the average N:C and P:C values for all the corals within that site. Error bars indicate standard errors of nutrient content. Ellipses were calculated using the Khachiyan algorithm^74^. The number of biological replicates at each site is available in Supplementary Table 1. **D** Hierarchical clustering of all metabolites enriched in high disturbance sites with the sum relative abundance of each chemical superclass, scaled to standard deviations of the mean (z-score). Full heatmaps including depleted metabolites are included in Supplementary Fig. 6. All panels are separated horizontally (left: *Montipora*, right: *Porites*). White horizontal lines divide each violin plot (Panels **A** & **B**) at the median. Panels **A**–**C** are colored by anthropogenic metabolome influence. Significant differences between clusters (Panels **A**–**C**) were compared using linear mixed models with pairwise significant differences assessed by FDR-corrected estimate marginal means comparison (*p < 0.05, **p < 0.01, ***p < 0.001; full statistical results are available in the Supplementary Data 1).

Metabolome energy reserves were significantly different between ecosystem disturbance clusters and correlated strongly with watershed and catchment metrics. To assess broad-scale shifts in metabolome composition and energy reserves, we evaluated the diversity of metabolites using Shannon diversity, the nutrient availability using nitrogen/phosphorus content, and the energetic availability of the metabolome using the nominal oxidation state of carbon (NOSC; all calculations are explained within the Methods) in both coral species metabolomes (Fig. 3). Elevated nitrogen and phosphorus^31^, and decreased ammonium^32^ are causally implicated with declines in coral health. Additionally, reduced water quality has led to decreases in lipid stores in corals^33^. In both coral species, Shannon diversity increased, and nitrogen content decreased in the sites with more anthropogenically influenced metabolomes (FDR-corrected estimate marginal means comparison *p* value < 0.001; Fig. 3; full statistical results are available in the Supplementary Data 1). In both corals, benthic phosphorus, benthic ammonium, benthic silicate, and onshore ecosystem disturbance explained 43.53% and 44.68% of the variance in Shannon diversity (*Montipora*: multiple linear regression *p* value < 0.001; *Porites*: multiple linear regression *p* value < 0.001; full statistical results are available in the Supplementary Data 1 and Table S1). In both corals, benthic phosphorus, benthic ammonium, benthic silicate, and onshore ecosystem disturbance explained 28.97% and 36.49% of variance in nitrogen content (*Montipora*: multiple linear regression p-value < 0.001; *Porites*: multiple linear regression *p* value < 0.001; full statistical results are available in the Supplementary Data 1 and Table S1). These results illustrate an increased metabolome diversity with reduced nutrient content of corals in sites with higher anthropogenic metabolome influence.

**Fig. 4:**
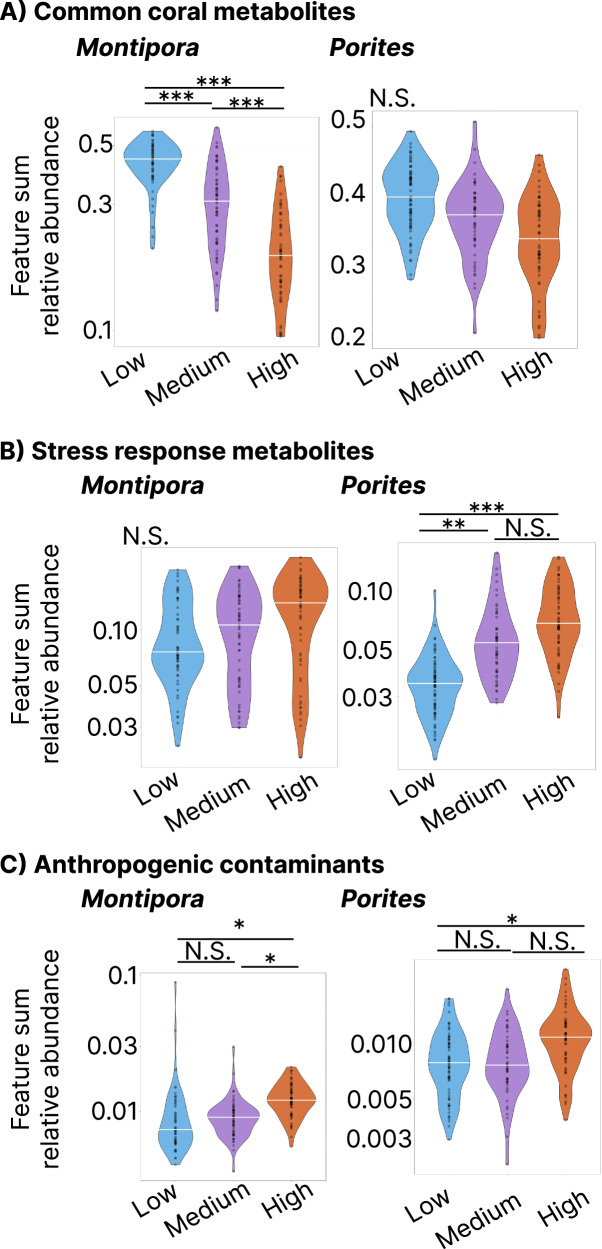
The relative abundance of coral metabolites is depleted with increased the amount of anthropogenic influence on the metabolome while anthropogenic contaminants are enriched. Sum abundance of common coral holobiont metabolites (**A**), stress response metabolites (**B**), and anthropogenic contaminants (**C**). Violin plots of site cluster enrichment of each type metabolite with medians marked as white horizontal bands. Pairwise significant differences assessed by FDR-corrected estimate marginal means comparison (***p < 0.001; **p < 0.01; *p < 0.05; N.S. = non-significant;; full statistical results are available in the Supplementary Data 1). Only metabolites with a library match that could be classified were included.

We posit that the increased metabolome diversity could reflect both an accumulation of anthropogenic contaminants and signal shifts in the coral metabolism resulting from increased ecosystem disturbances. Indeed, in our metabolome data we observed an increase in metabolome diversity, lower NOSC scores, and a depletion of metabolite nitrogen content in sites that are more heavily influenced. The reduction in nitrogen could be representative of increased stress as corals utilize nitrogen stores to metabolically respond to ecosystem stressors^34,35^. In *Montipora*, NOSC and phosphorus content were not significantly related to the amount of anthropogenic influence on the metabolomes (Fig. 3B, C; FDR-corrected estimate marginal means comparison *p* values > 0.05; full statistical results are available in the Supplementary Data 1). Benthic phosphorus, benthic ammonium, and benthic silicate explained 7.63% of the variance in NOSC (multiple linear regression *p* value = 0.0034; full statistical results are available in the Supplementary Data 1 and Table S1). In *Porites*, NOSC was significantly lower in more anthropogenically influenced metabolomes, while phosphorus content was higher in more anthropogenically influenced metabolomes (Fig. 3B, C; FDR-corrected estimate marginal means comparison *p* values < 0.001; full statistical results are available in the Supplementary Data 1). In *Porites*, benthic phosphorus, benthic ammonium, benthic silicate and onshore ecosystem disturbance explained 5.90% in NOSC score (multiple linear regression *p* value < 0.001; full statistical results are available in the Supplementary Data 1 and Table S1) and 30.53% in phosphorus content (multiple linear regression p-value < 0.001; full statistical results are available in the Supplementary Data 1 and Table S1). Lower NOSC scores in *Porites* at more influenced sites indicate that the metabolites within the coral metabolome are harder to degrade, as more reduced states (i.e., more negative NOSC) corresponds to harder to break structures^36,37^. The decrease in NOSC can be explained by two shifts in the metabolome: (1) both coral metabolomes were enriched in organoheterocyclics (Fig. 3D), which are recalcitrant to degradation^38^ and (2) increased phosphorus content within *Porites* tissues (Fig. 3C). The lower NOSC scores and organoheterocyclic compounds in coral tissues suggest an increased accumulation of recalcitrant metabolites, which overall reduce the availability of energy from metabolites within the coral metabolomes. Although an increase in phosphorus would suggest increased nutrient availability, 72.8% of all phosphorus-containing compounds were classified as the structural lipid, glycerophosphocholines. Lipids such as 1-(1Z-Hexadecenyl)-sn-glycero-3-phosphocholine^39^ (Fig. 4; Supplementary Data 1) and molecular species of phosphatidylcholine^40^ have previously been identified as structural lipids that incorporate into coral cell walls during stress^39,41–43^. We posit that because these structural lipids are a stress response and likely incorporated into the coral cells walls that the phosphorus they contain is not available for catabolism. Cumulatively, these results imply that corals in higher disturbance areas are using their nitrogen reserves to metabolically respond to environmental stress leaving their metabolomes depleted of nutrient rich and bioavailable metabolites.

All metabolite spectra that matched to a known library compounds were manually annotated as (i) common coral metabolites (metabolites previously associated with coral metabolomes), (ii) stress response metabolites (metabolites upregulated during stress), or (iii) anthropogenic contaminants (Supplementary Data 1; Supplementary Fig 7). Compared with the low metabolome influenced site cluster, highly influenced areas had a lower relative abundance of common coral metabolites by 50.9% in *Montipora* (Fig. 4A; FDR-corrected estimate marginal means comparison *p* value < 0.001; full statistical results are available in the Supplementary Data 1). In *Porites*, there was a relationship between common coral metabolites and amount of metabolome influence (linear mixed model *p* value = 0.028, df = 2), although pairwise comparisons between the groups were not significant (FDR-corrected estimate marginal means comparison *p* value > 0.05; full statistical results are available in the Supplementary Data 1). Potential stress metabolites in highly influenced areas were 113% higher in *Porites* than in the low metabolome influence cluster (Fig. 4B; FDR-corrected estimate marginal means comparison p-value < 0.001; full statistical results are available in the Supplementary Data 1) but were not significantly different for *Montipora* (Linear mixed model *p* value > 0.05, df = 2).The reduced nitrogen reserves in impacted coral communities concurrent with a greater diversity of metabolites (Fig. 3A), enrichment of hard to degrade compound (Fig. 3D) and increased stress responses (Fig. 4B), support our hypothesis that corals under worsening ecosystem conditions must use significant amounts of their nitrogen and energetic reserves to maintain homeostasis. This could largely reduce the resilience of these impacted corals as without available carbon and nitrogen, corals cannot maintain homeostasis, making them more susceptible to tissue damage, symbiont instability/loss, and disease^34,35^. We posit that human activities greatly reduce the resilience of nearby coral communities to additional stressors as the metabolomes of these impacted corals will have reduced energy reserves, although further experiments are needed to identify a direct link between coral resilience and metabolome energy reserves.

We measured metabolites derived from pharmaceutical, industrial, cosmetic, and agricultural sources in coral tissues (Fig. 4C). The relative abundances of anthropogenic contaminants increased significantly from low to high metabolome influence sites in both *Montipora* (26.0%; Fig. 4C; FDR-corrected estimate marginal means comparison *p* value < 0.01; full statistical results are available in the Supplementary Data 1) and *Porites* (32.8%; FDR-corrected estimate marginal means comparison *p* value = 0.048; full statistical results are available in the Supplementary Data 1). Similar to other animals^44,45^, exposure to contaminants impacts reef-building corals at multiple life stages^9^. For example, accumulation of contaminants, like octocrylene, is linked to mitochondrial dysfunction in corals^10^. Our work expands on this finding by identifying contaminants of concern that are accumulating in the tissues of reef corals. Furthermore, we show that ecosystem disturbance explains patterns in the accumulation of these compounds.

## Discussion

Coral reef ecosystem disturbances have continued to accelerate with changes in climate and land-use practices^3^. Here, we have identified two separate mechanisms by which coral resilience is potentially reduced as a function of ecosystem disturbance. First, the increased disturbance depletes the metabolomes of nitrogen and bioavailable metabolite reserves. Second, the metabolomes accumulate anthropogenic contaminants with untold physiological consequences. Coral physiology is becoming increasingly altered by the accumulation and impacts of anthropogenic contaminants introduced by human land use, and this ultimately contributes to declines in coral cover among near-shore, human-associated coral reef habitats^46,47^. We demonstrate, along 70 km of coastline, the multiple ways coral reefs are directly affected by human land use. In areas of increased land and catchment disturbance, corals accumulate a wide range of agricultural products, pharmaceuticals, and industrial contaminants. Bioaccumulation of pollutants in an array of anthropogenically disturbed sites highlights the lasting impact of human activities on coral reefs. It is not clear how the anthropogenic contaminants that we detected in our study impact coral health, but targeted studies have demonstrated previously demonstrated direct impacts of other pollutants. For example, Jafarabadi et al., (2021) demonstrated sediment-vectoring of pollutants to reefs which increased bleaching associated with persistent organic pollutants^11^. Further, as reviewed in both Ouédraogo et al. (2023)^48^ and Nalley et al. (2021)^9^, numerous pharmaceuticals, hydrocarbons, and pesticides have been shown to impact coral physiology, mortality rates, bleaching susceptibility, gamete fertilization, and settlement. In other systems these contaminants have been demonstrated to cause larval deformation, modify feeding behavior, and increase mortality in zebrafish, daphnia, and mussels even well below accepted environmental concentrations^44,49^.

As mixotrophic sessile organisms, corals are especially capable of bioaccumulating contaminants through both the uptake of dissolved contaminants and the biomagnification of contaminants via heterotrophic feeding^50–52^. While this does mean corals are more at risk from anthropogenic contaminants, our results demonstrate that monitoring sessile reef metabolomes may be a useful environmental survey indicator of anthropogenic ecosystem disturbance. Being sessile, the chemicals bioaccumulated in coral tissues serve as a marker of both short term and chronic pollution, giving a less temporally dependent assessment of contaminant exposure compared to the discrete measurements derived from water quality monitoring^53^. Further, contaminants within organismal metabolomes demonstrate the biological uptake and accumulation of contaminants that sediment cores and water samples cannot reveal^54^. Tissue collections, especially of endangered coral species, do have their drawbacks. However, newer techniques such as the 16-guage tissue punch protocol from Berg et al., (2025), minimize the invasiveness of these methods^55^. Future studies should further compare tissue metabolome characterization with sediment and water monitoring strategies to understand the potential for metabolomics to expand the abilities of reef monitoring and management. Additionally, the extraction solvent can drastically shift our analytical window^56–58^. While there have been recent efforts to standardize the extraction methodology for coral metabolomics^58^, currently there have been no direct comparisons of coral tissue extraction efficiencies across different solvents. In context with this study this means that there are likely more anthropogenic contaminants and other metabolites that lie outside of our analytical window.

As suggested by our data, existing management practices are insufficient to address the effects of watershed development. This is evident at Site 3 where The Kahekili Herbivore Fisheries Management Area (KHFMA) has prohibited the take of herbivorous fishes and urchins since 2009 and banned spearfishing by SCUBA since 2013^30^. While this was previously a successful management strategy, since the 2015/2016 bleaching event, this site has significantly declined in coral cover despite the KHFMA’s continued efforts. Understanding the conditions that predicate coral resilience are essential for both protection and restoration of coral reefs. The unseen effects of coastal development impede our ability to model, manage, and mediate reef health under a changing climate, and represent a previously unrecognized lens through which the effectiveness of management efforts must be considered. Recent work from Kalinski et al. (2026), demonstrated the nearly ubiquitous contamination of marine systems by anthropogenic contaminants^59^. As chemicals known to disrupt animal development and microbial communities continue to be accumulated by human-adjacent coral reefs, the contaminant-induced disruption of homeostasis will further disrupt reef communities across multiple, if not all, trophic levels and ultimately reduce their resilience under climate change^60^. Moreover, contamination of edible marine life will disproportionately impact indigenous, low income and marginalized communities that live near and draw resources from reefs^61^.

## Methods

### Sample collection

In March of 2018, fragments of approximately 186 *Montipora capitata*, and 193 *Porites lobata* colonies were collected from 16 locations across 70 km of coastline on West and South Maui. Coral samples were collected from visually healthy colonies using a hammer and chisel following methods described by Greene et al.^62^ with a section of skeleton and tissue 2 cm^2^ placed in 15 ml of HPLC grade methanol (Sigma Aldrich) 5.17 µmol L^–1^ 2-aminoanthracene (Sigma Aldrich) on ice for metabolite extraction and transferred to a freezer as soon as possible at –20 °C^62^. 2-aminoanthacene was dissolved into stock HPLC grade methanol prior to aliquoting into glass scintillation vials for later use as a quality control indicator. Sample collection and exchange permitting was provided by the Hawaii Department of Land and Natural Resources under SAP 2018-03 and CITES secretariat under permit #17US86408A/9. Approximately 11–15 samples were collected per sampling location with an effort made to standardize colony depth (mean 4 m) and colony size (mean 26 cm) across all reef sites. At each of the 16 coral sampling locations one 500 ml water sample was collected approximately 0.5 m above the reef using a large syringe. At the surface, 50 ml from the benthic water sample was aliquoted into amber glass vials and stored at 4 °C for later water quality analysis. Water samples were shipped immediately after collection to the University of Hawaii School of Ocean and Earth Science and Technology Laboratory for Analytical Biogeochemistry (S-LAB) for analysis of ammonia, phosphate, silicate, and nitrate/nitrite content on a Seal Analytical AA3 HR Nutrient Autoanalyzer (Seal Analytical, Southampton, United Kingdom).

### Environmental data

Historic percent coral cover data for Sites 1, 3, 8, 11, and 15 were retrieved from the Coral Reef Assessment and Monitoring Program (CRAMP)^26–28^. Satellite estimates of onshore habitat disturbance were generated using the Carbon Assessment of Hawaii Land Cover Map where proportional area of “Heavily Disturbed” habitat was calculated for a 3 km circular buffer immediately onshore of each coral sampling location using the software QGIS (version 3.18). It should be noted that we were unable to locate measures of beach use or vessel traffic to reef sites to use as explanatory variables. It appears that such data are either not collected or not made publicly available by the State of Hawaii and its county governments.

### Liquid chromatography tandem mass spectrometry data acquisition

Coral samples in LCMS methanol 5.17 µmol L^-1^ 2-aminoanthracene were allowed to extract for approximately 12 months and 200 µl of extractant aliquoted for shipment to and processing by Metabolomics Australia (University of Melbourne, Australia). It should be noted that this extraction period is longer than necessary and was the result of logistical complications. A further discussion of freezing length and temperature is available in the supplementary discussion. Metabolite separation was performed on a Vanquish Horizon UHPLC system (Thermo Scientific) coupled to an Orbitrap ID-X Tribrid mass spectrometer (Thermo Scientific) for metabolite detection by Metabolomics Australia. The chromatography conditions were modified from Tsugawa et al.^63^. In brief, separation was performed on an Acquity bridged ethyl hybrid C18 column (1.7 μm, 2.1 mm × 100 mm, Waters). The column compartment temperature was maintained at 40 °C. The mobile phases consisted of A (0.1% formic acid in H2O) and B (0.1% formic acid in acetonitrile). The following gradient was applied: 0–0.1 min, 0.5% B; 10 min, 80.0% B; 10.1 -min, 99.5% B; 12 min, 90% followed by a column wash-out phase between 10.1-14.5 min at a flowrate 400 μL·min–1, before returning to 300 μL·min–1 at 14.6 min for column re-equilibration. The samples were kept at 4 °C in the autosampler. The injection volume was 10 μL for all coral samples. Please note that the duration of methanol extraction reflects delays due to shipping and permitting restrictions; shorter extraction periods are recommended^63^.

Metabolite detection was performed on a Orbitrap ID-X Tribrid Mass Spectrometer (Thermo Scientific) coupled to heated electrospray ionisation (H-ESI) source with the following conditions: sheath gas flow 40 arbitrary units (Arb), auxiliary gas flow 10 Arb, sweep gas flow 1 Arb, ion transfer tube temperature 275 °C, and vaporizer temperature 320 °C. The RF lens value was 35%. Data was acquired in positive polarity with spray voltages of 3500 V. A data-dependent acquisition (DDA) was used for the coral samples with MS1 scans acquired at a resolution of 60 K with a standard AGC target (system determined target) and a maximum injection time of 100 ms. MS2 spectra were collected on [M + H]+ ions in positive polarity with an isolation window was set to 1.5 m/z. A stepped HCD Collision Energies of 0, 10, 20 and 30% were used as the collision energy. MS2 data were acquired with 15 K resolution, a standard AGC target, a maximum injection time of 100 ms, and a dynamic exclusion of 2 s. A 5 ppm mass tolerance was used for the detection of inclusion list entries, and the intensity threshold was set to 2.5e5.

### Feature detection, molecular networking and annotation of MS/MS data

Feature finding was performed with MZmine 4 (v4.1.0)^64^ and all the parameters for this study are listed in Supplementary Data 1 1. A Feature Based Molecular Network (FBMN)^65^ was built using the Global Natural Products Social Molecular Networking (GNPS) online platform^66^. Features (defined as a detected ion signal with a MS/MS spectra) were connected to spectrally similar features (defined as spectra with at least 4 matched peaks and a cosine score greater than 0.7) to form clusters of spectrally similar features (herein called subnetworks). The maximum size of a subnetwork was set to 100 features with the lowest scoring edges removed until the size was below the threshold. Features that were not spectrally similar to any other features were left orphaned (herein called singe-loop nodes). GNPS additionally matched experimental spectra to spectral libraries of previously identified compounds. Features that were matched to known library spectra were manually annotated with their prospective source based on previously identified literature (Supplementary Data 1 1). Each library match was additionally confirmed based on cosine score (>0.7), m/z, and mirror match alignment (Supplementary Fig 8). SIRIUS 4 (v6.3.4)^67^ provided molecular formulae (SIRIUS and ZODIAC frameworks) and neural network predicted structures^68^ (CANOPUS framework) for each feature using the parameters defined in Supplementary Data 1 1.

### Library match source annotation

The sources of each library match from GNPS were annotated based on a uniform literature search for prevalence of the compound in: (a) industrial, diet supplementation, cosmetological, or agricultural processes, (b) coral, (c) coral metabolic usage. Annotations, and cosine scores for each library match in this analysis are available in Supplementary Data 1 1.

### Nominal oxidation state of carbon score and nitrogen content calculations

Elemental formula predicted from SIRIUS and Zodiac^67^. Following the equations described within Wegley et al.^69^, the nominal oxidation state of carbon (NOSC) was calculated for each feature (Eq. 1):

1 $${NOSC}=-\frac{z+4a+b-3c-2d+5e-2f}{a}+4$$

In Eq. 1, z corresponds to the net charge of the organic compound and the subscripts a, b, c, d, e, and f refer to the stoichiometric numbers of the elements C, H, N, O, P and S, respectively. A NOSC score was calculated for each metabolite in each sample by weighting the NOSC value of that metabolite by its relative abundance. Nitrogen contribution of each metabolite in the sample was assessed by weighing each metabolite’s nitrogen to carbon ratio by the relative abundance of that metabolite. The nitrogen content of the entire metabolome was calculated by taking the sum value of every metabolite nitrogen contribution within each sample. The phosphorus content of the entire metabolome was calculated similarly.

### Metabolite feature cleaning

As each coral species was run in separate batches, the cheminformatic pipelines were run separately for each coral species with their results summarized in Supplementary Table 2. In total 2142 features (belonging to 615 subnetworks/single-loop nodes) were identified by the MzMine feature finding. To remove background and contaminant features, the chromatogram intensity (XIC) of each feature was compared between experimental blanks and samples. A background feature was defined as any feature that had an XIC in any blank that was twice as high as the average XIC across all the samples. A second filter removed entire subnetworks identified as background subnetworks by finding subnetworks wherein greater than 70% of the features were background features. After removing background features and subnetworks, a total of 1426 and 1315 features (belonging to 400 and 386 subnetworks/single-loop nodes) remained in the analysis for *Montipora capitata* and *Porites lobata*, respectively. A final filter removed rare features to eliminate potential machine noise and sample carry-over. Features were described as rare if their max XIC was below the first quartile of max XIC. From herein, all features that passed both background removal and rare filtration (*n* = 966 features in 266 subnetworks/single-loop nodes for *Montipora capitata* and *n* = 786 features in 240 subnetworks/single-loop nodes for *Porites lobata*) are classified as metabolites.

### Statistical analyses

All data pipelines, statistics, and figure panels were made in R (v4.5.1) using the libraries: ape, car, CHNOSZ, data.table, dendextend, DescTools, ggforce, lme4, MuMIn, pheatmap, randomForest, scales, tidyverse, pairwiseAdonis, and vegan. Linear models of percent coral cover were modeled with a breakpoint between the 2014 and 2015 surveys as 2015 marks the beginning the third global coral bleaching event which significantly impacted Hawaiian coral reefs^29^. To compare compositional differences between samples, metabolite XIC values were standardized to relative abundance of that metabolite within its sample by dividing the metabolite XIC value by its sample sum XIC (also referred to as total ion chromatogram or TIC). It should be noted that the XIC values and relative abundances within each sample are not quantitative (e.g., not equivalent to µM concentrations) and all comparisons in this manuscript are comparing the percent of total ion intensity within each sample to that of another sample. To approximate a gaussian distribution for linear statistics, relative abundances were angularly transformed (asin(√relative abundance)) and environmental analytes were log_10_ transformed. To identify molecular families that vary between our sampling sites we tested the effect of site number on subnetwork abundance with metabolite number as a random effect using a linear mixed model (subnetwork abundance ~ siteNumber + (1|metabolite)). All metabolites and molecular families presented herein were limited to those that were significantly different between sites as defined from the linear mixed model (false discovery rate corrected LMER *p* value < 0.05; full statistical results are available in the Supplementary Data 1). Significant separation and dispersion of metabolome clusters in multidimensional space was tested using PERMANOVA tests using the adonis2 and betadisper functions from the Vegan R package^70^ with pairwise PERMANOVA’s subsequently tested using the pairwiseAdonis2 function from pairwiseAdonis R Package^71^. Dispersion and group separation were tested using bray-curits distance matrices and in the case of *Montipora* did not include the outgroup site (site 12). To evaluate how much variance in metabolome chemodiversity, nitrogen content, phosphorus content, and NOSC score was explained by environmental parameters, multiple linear regression models were used, following a parameterization using the dredge average model (MuMIn v1.43.17; AICc <2). The linear regression compared each corals coordinate value extracted from PCoA axis 1 to the site environmental parameters (Supplementary Fig. 4). PCoA’s were built using bray-curtis distances of chemical superclass abundance. To compare metabolome shannon diversity, NOSC score, metabolome nitrogen/phosphorus content, superclass sum abundance, and metabolite sources, a linear mixed model tested for significant differences between our site clusters on each response variable with site code as a random effect (nutrient content ~ HCA + (1|siteCode)). Pairwise comparisons between site clusters in nutrient, NOSC, and contaminant accumulation were contrasted using Tukey-adjusted post-hoc tests based by computing the estimated marginal means using the *emmeans* package^72^. All p-values were false-discovery rate corrected^73^.

### Site clustering

Sites were clustered based on hierarchical clustering of ion features with the XIC summed at the subclass classification level (Fig. 1, S1). Hierarchical clustering was performed using Euclidian distances with dendrogram linkages generated using Ward’s minimum variance method. The only two sites that clustered differently between coral species were grouped according to cluster membership separately for each species in all downstream analyses (see the Supplementary Discussion for a discussion on taxa differences). Site 1 was assigned to cluster 2 in *Montipora* whilst for Porites aligned with cluster 1. Site 12 grouped with cluster 2 in the *Porites* analyses (Supplementary Figs. 1–3, 5) but was not aligned with any cluster for downstream *Montipora* analyses (Fig. 1).

### Reporting summary

Further information on research design is available in the Nature Portfolio Reporting Summary linked to this article.

## Supplementary information

Supplementary Information

Peer Review file

Description of Additional Supplementary Files

Supplementary Data 1

Reporting Summary

## Data Availability

The raw MS spectra generated in this study have been deposited in the MASSIVE database under the accession code MSV000098452 [https://massive.ucsd.edu/ProteoSAFe/dataset.jsp?task=4e8bd88197fb4bba8c802a2bb5d25220]. The raw data, plots and post-processed data frames has been deposited into Github and archived using Zenodo under the 10.5281/zenodo.20314872. Historical coral cover data was provided and available through the Coral Reef Assessment and Monitoring Program (CRAMP). Watershed disturbance was retrieved from the Carbon Assessment of Hawaii Land Cover Map (https://geoportal.hawaii.gov/datasets/HiStateGIS::carbon-assessment-of-hawaii-land-cover-biome-unit/about).

## Code availability

The custom-written code generated in this manuscript has been deposited into Github and archived using Zenodo under the 10.5281/zenodo.20314872.
