# Supplementary material for: Coral metabolome quality and contaminant loads track human land use
Source: Nat Commun. 2026 Jul 15;17:6034. doi: 10.1038/s41467-026-74960-7 (PMC13373168; doi:10.1038/s41467-026-74960-7)
Supplement: Supplementary file 1 — Supplementary Information [file 41467_2026_74960_MOESM1_ESM.pdf]

Supplementary Information

Coral metabolome quality and contaminant loads track human land use

Authors: Zachary A. Quinlan, Austin Greene, William Leggat, Tess Moriarty, Tracy D. Ainsworth, Brunda Nijagal, Kim Falinski, E. Maggie Sogin, Jamie M. Caldwell, Scott F. Heron, Megan J. Donahue

Supplemental Discussion... 2

Figure S1. Porites tissue metabolites cluster similarly to Montipora.. 6

Figure S2. Principal coordinates analysis (PcOA) displaying the clustering of sites for both Montipora and Porites tissues using different metabolite calculations... 7

Figure S3. Hierarchical clustering of sites using full metabolome dataset and not only those that differed significantly between sites... 8

Figure S4. Primary component analysis (PcOA) of sites colored by the three clusters of metabolites in the hierarchical clustering (Fig 1)... 9

Figure S5. Removal of site 12 enriched molecular families in Montipora tissues to investigate clustering differences between Porites and Montipora tissues..10

Figure S6. Tissue chemical makeup shows distinct patterns of enrichment within broad chemical classifications..12

Figure S7. Hierarchical cluster of enriched features with library ID's..14

Figure S8. Mirror plots comparing experimental spectra (top) to library spectra (bottom). Cosine scores indicate peak alignment within each mirror plot.. 15

Figure S8-continued.. 16

Table S1. Number of coral tissue samples per site (biological replicates).................................17

Table S2. Feature and molecular families/single-loop nodes (SLN) remaining after each step in the informatic pipeline.. 17

References:... 18

Supplemental Discussion

Differences between coral taxa

The two species evaluated within this study (*Montipora capitata* and *Porites lobata*) are morphologically distinct and vary greatly in life-history and growth strategies (Dubinikas, 2017). Surprisingly the majority of the sites maintained the mostly the same cluster designations within our analysis with the obvious exceptions of Site 1 and Site 12. Site 1 moved from cluster 2 (medium metabolome influence) in *Montipora* samples to cluster 1 in *Porites* (low metabolome). Although the clustering of sites still explains 71.4% of the variation in *Porites* metabolomes (PERMANOVA $p < 0.01$), the cluster dendrogram (Supplemental Figure 3) and overlap of the clusters in multidimensional space (Figure 1B) suggest the pattern underlying changes in *Porites* metabolomes may be continuous rather than discrete. This could explain some of the differences in site membership between the two corals: Site 12 grouped with cluster 2 in the *Porites* analyses but was a clear outgroup in *Montipora* samples. The differences in clustering was seemingly because of a single cluster of molecular families enriched in Site 12 evidenced by their removal producing the same clustering of sites in *Montipora* as *Porites* (Supplemental Figure 5). Of the 15 molecular families enriched at Site 12, only 4 had features with library matches. Molecular family 83 had library matches for Lauryldiethanolamine, Tetradecyldiethanolamine, Arachidoyl Ethanolamide, and C17-Sphinganine. Lauryldiethanolamine was the only clear contaminant as it is a well classified MS microtube contaminant. Whereas Tetradecyldiethanolamine was classified as an anthropogenic contaminant that is currently patented for use in industrial settings (Supplemental Table 2), and Arachidoyl Ethanolamide is a pharmaceutically active compound. The source of C17-Sphinganine is unknown. The single-node 145 was classified as the pesticide DEET. The library matches from molecular families 1020, 117 and 46 all had low cosine scores

and poor mirror match alignment and thus could not be annotated further. Although these metabolites may be contaminants, because of the high level of uncertainty in their annotations or potential sources, we chose to keep them in our dataset but instead remove Site 12 from the analysis of *Montipora* tissues.

Misidentification of metabolites

It is possible that our analysis could be misclassifying some of the compounds herein as many of the biologically active compounds in pharmaceuticals are natural products (Harvey 2008). Conversely, this analysis could also be misclassifying some contaminants as common coral metabolites. There is some evidence for the latter scenario within the subnetwork highlighted in Supplemental Figure 6. Molecular networking builds subnetworks based on structural similarities (cosine scores; Wang et al., 2016) and as a result we are able to putatively identify previously unnamed compounds because of their structural similarity to known spectra.

Freezing temperatures

The methanol extracts from our samples were stored at -20˚ C for one year. Best practices for untargeted metabolomics, suggest freezing at -80˚ C reduces the possibility of metabolite degradation. This could potentially risk the stability of some molecules however it is unlikely to bias our conclusions because: 1) All samples were stored together and since we only compare across sites any degradation would be consistent across all samples, 2) All samples were stored in Methanol with reduces the enzymatic activity and thus degradation of metabolites, 3) To our knowledge there have been no studies that test metabolite stability in methanol and/or ethanol at -20˚ C compared to -80˚ C.

For metabolomics, methanol is widely considered the best storage solvent because it quenches enzymatic activity increasing metabolite stability and its broad metabolite coverage (Sitnikov et al., 2016). Although there is still a risk that we may have observed more anthropogenic metabolites if our samples were stored at -80˚ C instead of -20˚ C, the decreased enzymatic activity within methanolic extracts and relative stability at -20˚ C observed in other sample types suggests there would be minimal degradation over 12 months. While the stability of metabolites in no extraction solvent over extended storage times (Petrick et al., 2024; Kalbitzer, and Heistermann 2013) or at higher temperatures (Petrick et al., 2024; Kalbitzer, and Heistermann 2013), and stability across different extraction solvents (Kalbitzer, and Heistermann 2013) have all been tested separately, there have been no direct tests of metabolite stability in methanol and/or ethanol at -20˚ C compared to -80˚ C. Petrick et al., (2024) demonstrated that in dried blood samples, storage at -20˚ C remained similar to those stored at -80˚ C over a 6 month period, although plasma had slightly decreased stability over the same time scale. Gondo et al., (2024) also found that fruit drinks remained stable for at least one year when stored at -20˚ C compared to -80˚ C.

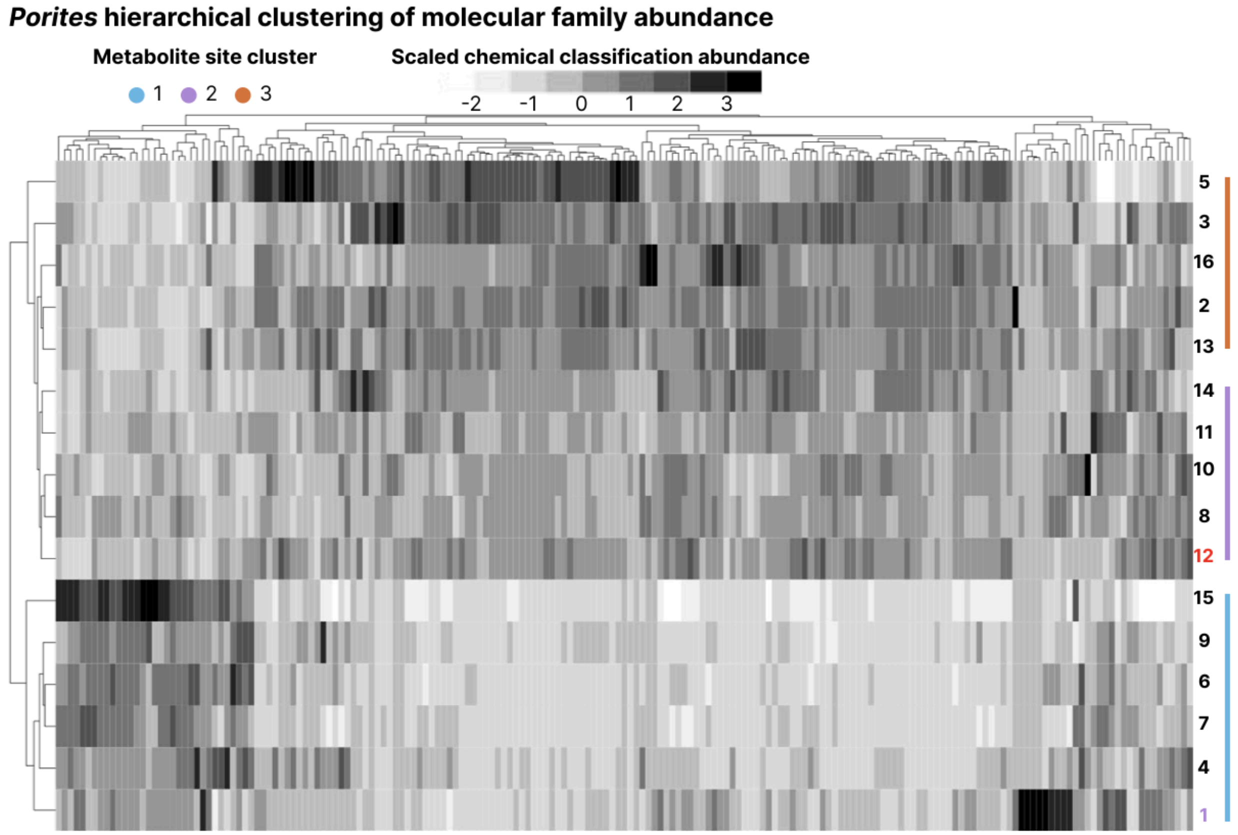

Figure S1. Porites tissue metabolites cluster similarly to Montipora.

Hierarchical clustering of molecular family abundance in Porites tissue. Cells are shaded by z-scored relative abundance of each molecular family. Sites 12 and 1 are colored by the metabolite cluster they belong to in the Montipora hierarchical clustering (Figure 1).

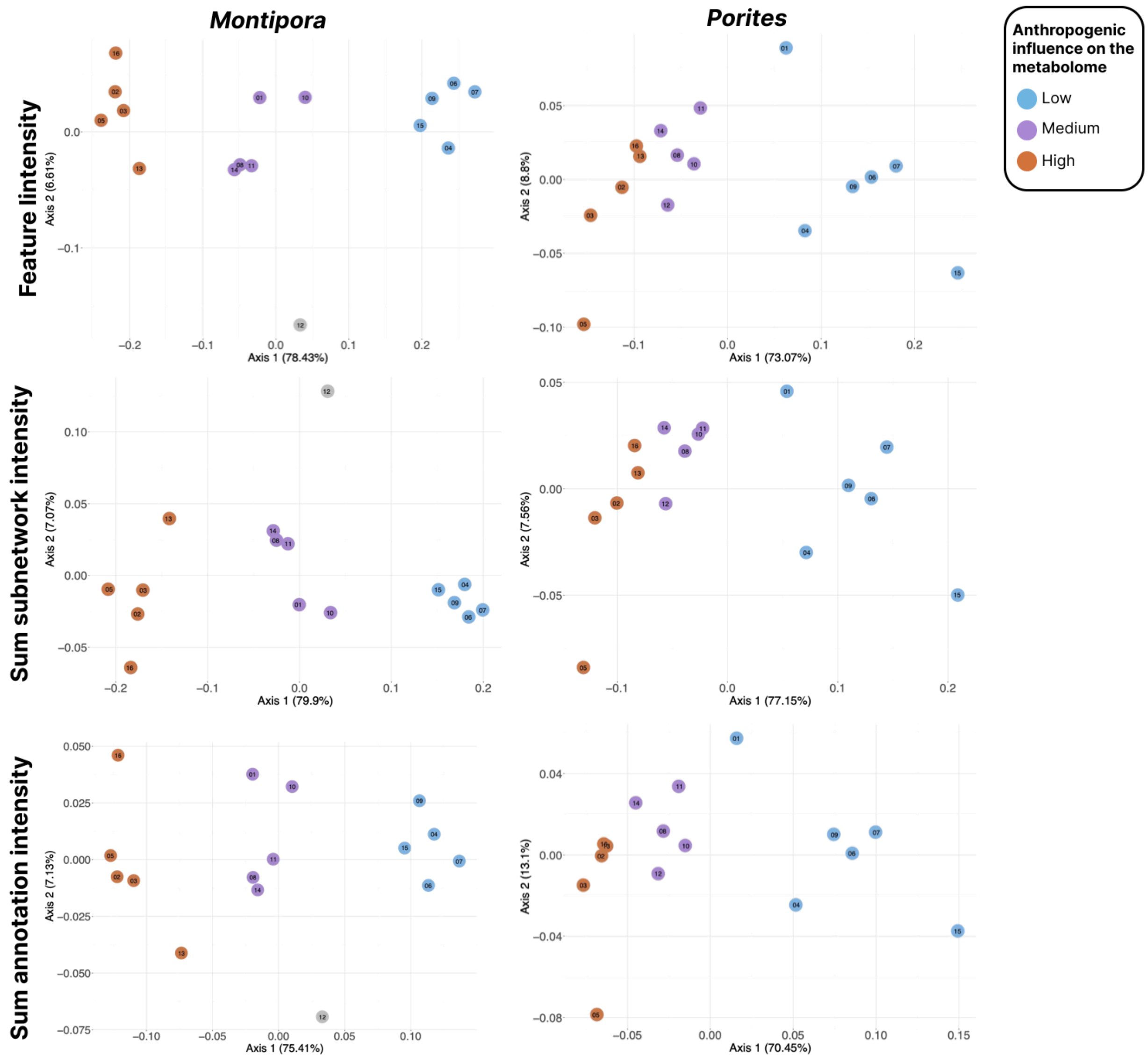

Figure S2. Principal coordinates analysis (PcOA) displaying the clustering of sites for both *Montipora* and *Porites* tissues using different metabolite calculations.

PcOA's were calculated from Bray-Curtis distances of the site average feature abundance (top), molecular family abundance (middle), or *in-silico* predicted annotation abundance (bottom). Each metabolite cluster is classified as less (blue), moderately (purple) or highly (orange) influenced by human activities.

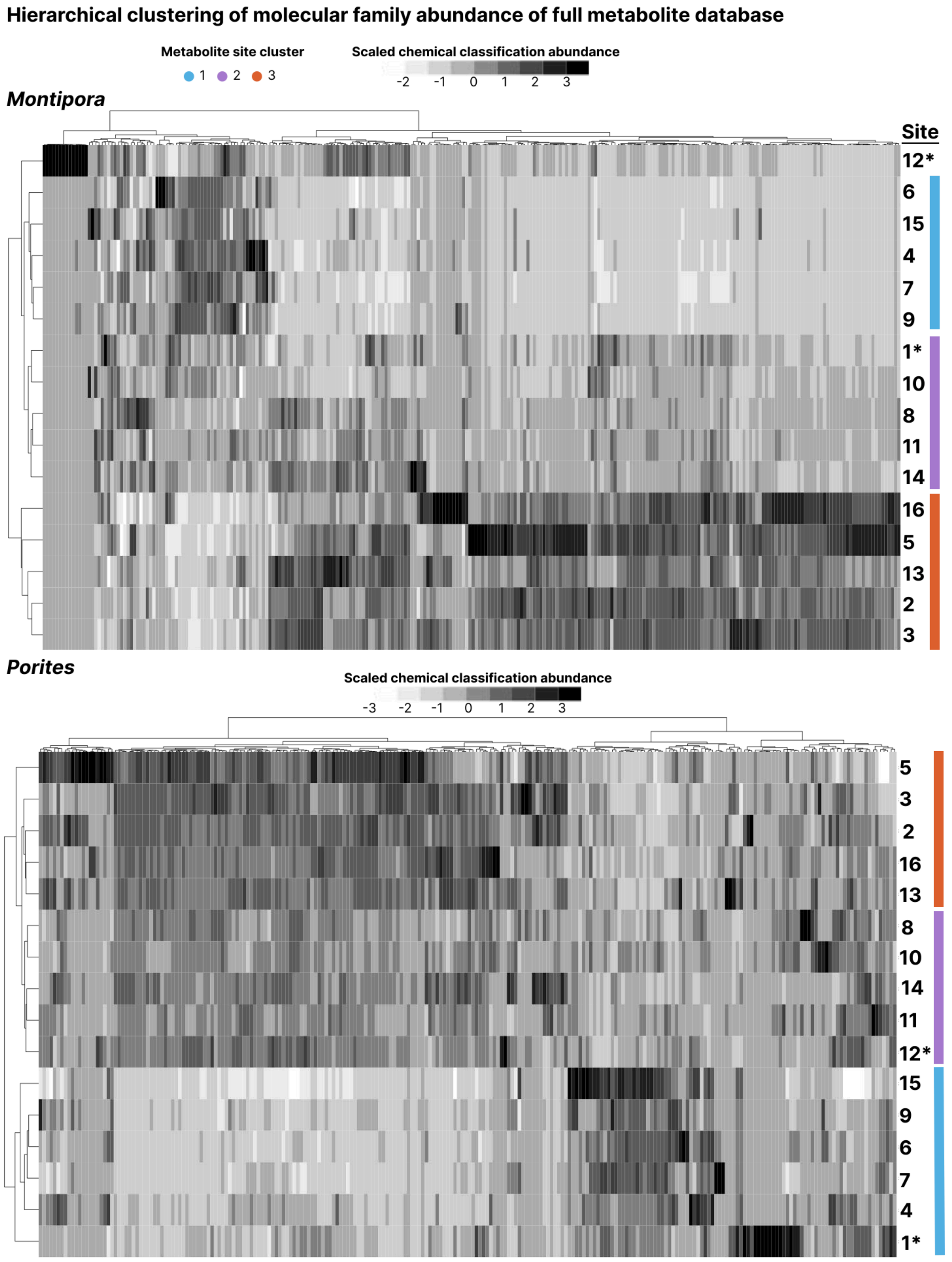

Figure S3. Hierarchical clustering of sites using full metabolome dataset and not only those that differed significantly between sites.

Hierarchical clusters were built using metabolite relative abundance summed within molecular families for each sample and averaged for each site. Cells are shaded by z-scored relative abundance of each molecular family. Clustering was performed using Euclidian distances. Dendrogram linkages were generated using Ward's minimum variance method. Asterisks denote sites that changed group classification between each species.

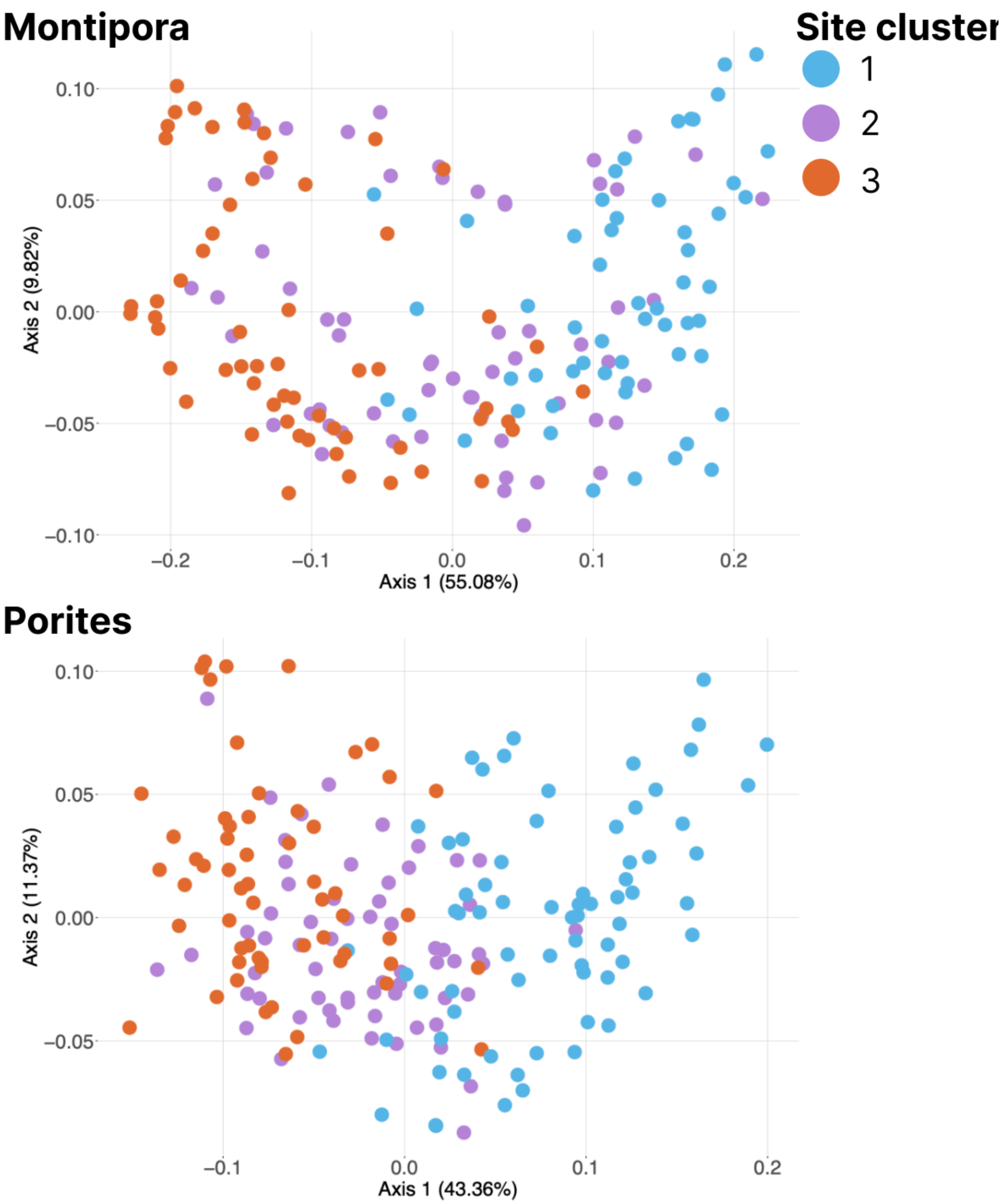

Figure S4. Primary component analysis (PcOA) of sites colored by the three clusters of metabolites in the hierarchical clustering (Fig 1).

Ion feature sum intensities were calculated at the most narrow subclass chemical annotation. Distance matrix was calculated using Bray-Curtis distances of chemical superclass relative abundances.

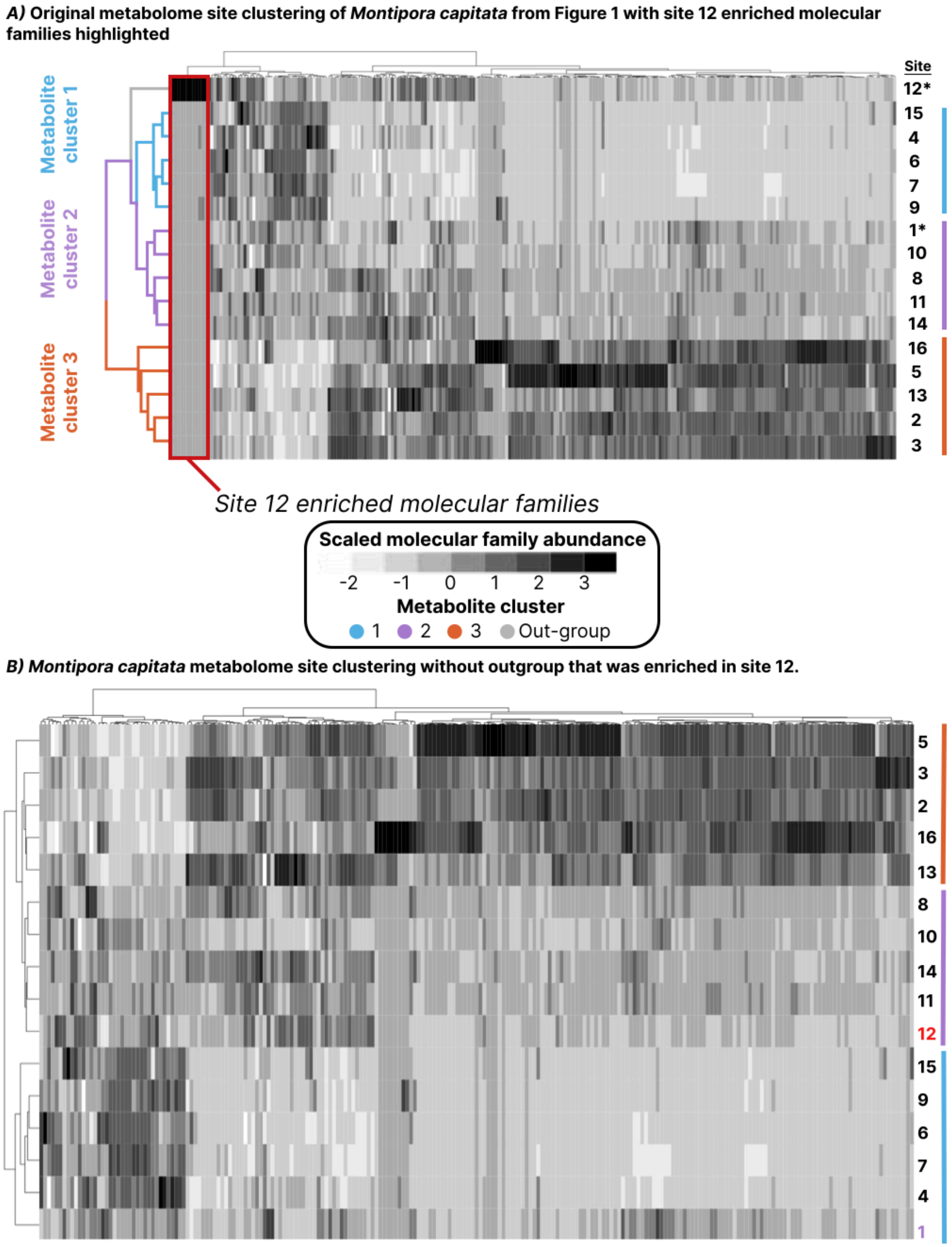

Figure S5. Removal of site 12 enriched molecular families in *Montipora* tissues to investigate clustering differences between *Porites* and *Montipora* tissues.

A) Original hierarchical clustering of *Montipora* metabolomes from Figure 1 with site 12 enriched molecular families highlighted with the red rectangle. B) Hierarchical clustering of *Montipora* tissues when the site 12 enriched molecular families were removed from the clustering. Cells

are shaded by z-scored relative abundance of each molecular family. Clustering was performed using Euclidian distances. Dendrogram linkages were generated using Ward’s minimum variance method. Asterisks denote sites that changed group classification between each species.

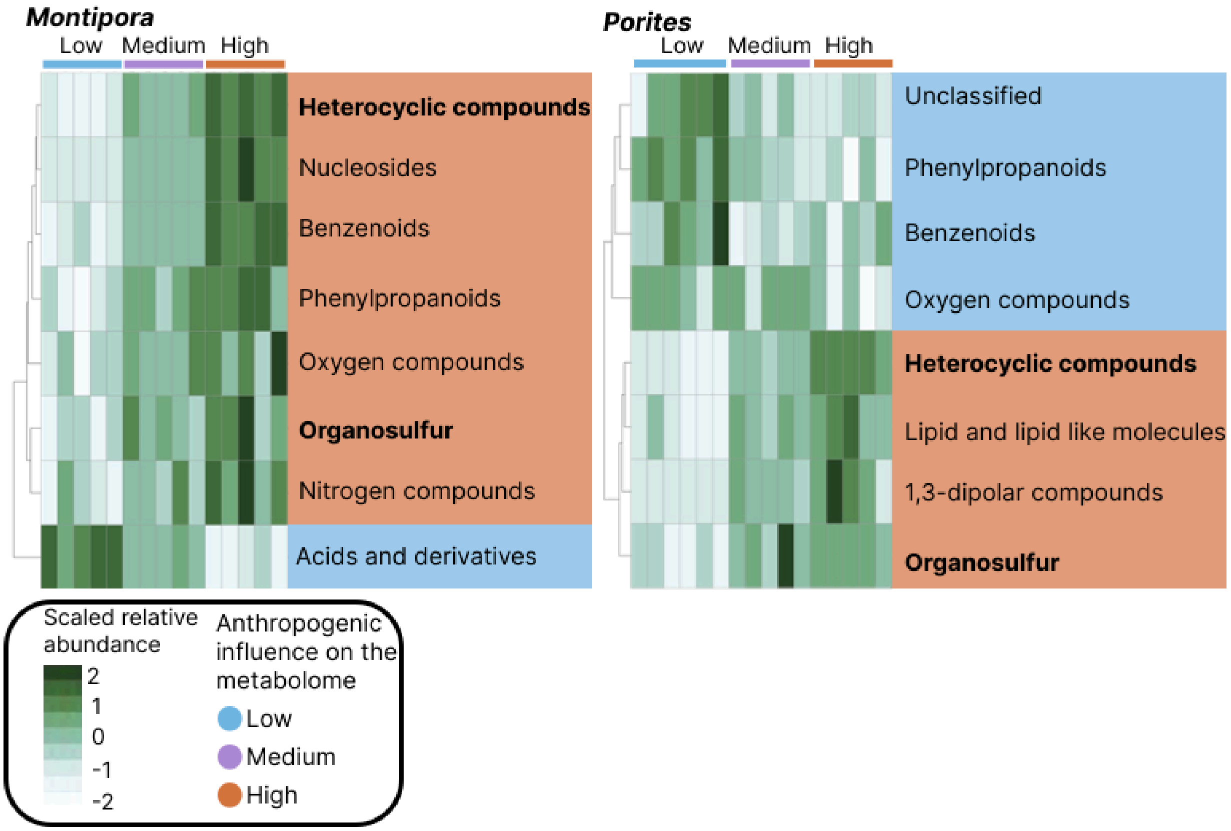

Figure S6. Tissue chemical makeup shows distinct patterns of enrichment within broad chemical classifications.

Hierarchical clustering of sum abundance of each chemical superclass, scaled to standard deviations of the mean (z-score). The backgrounds of each superclass names are colored based on the site cluster in which they are enriched. Only superclasses with significant differences between site clustering (as defined by a linear mixed model FDR corrected p-value < 0.05; asin(sqrt(ra))~ HCA + (1|Site) are visualized for each species (left: *Montipora*; right: *Porites*). Sites are colored by their metabolite site cluster.

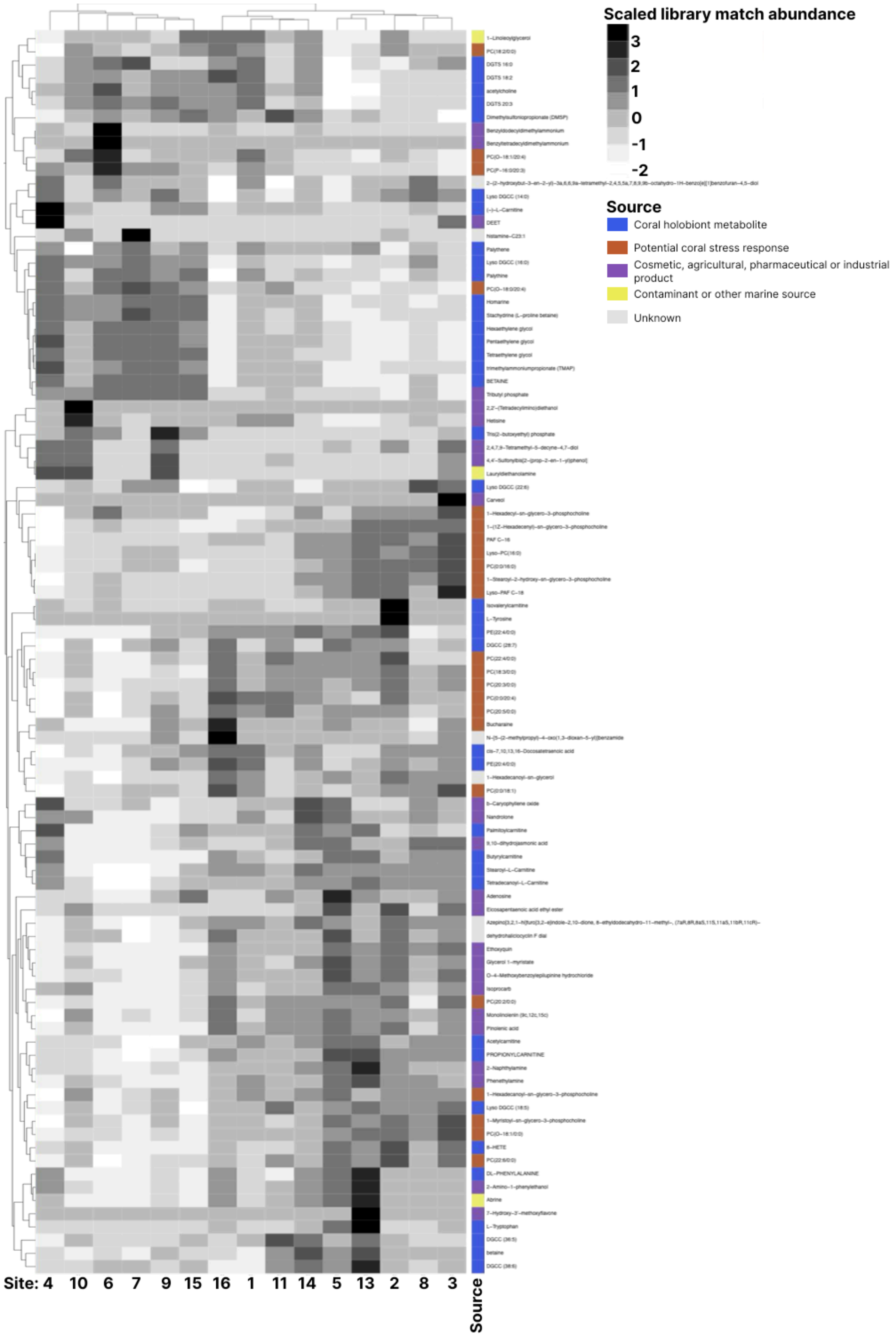

Scaled library match abundance
3
2
1
0
-1
-2
Source
Coral holobiont metabolite
Potential coral stress response
Cosmetic, agricultural, pharmaceutical or industrial product
Contaminant or other marine source
Unknown
Site: 4 10 6 7 9 15 16 1 11 14 5 13 2 8 3
Source

Figure S7. Hierarchical cluster of enriched features with library ID's.

The source of each metabolite is colored along the right side of the hierarchical cluster. Each cell of the heatmap is colored by the z-score scaled relative abundance of the library match.

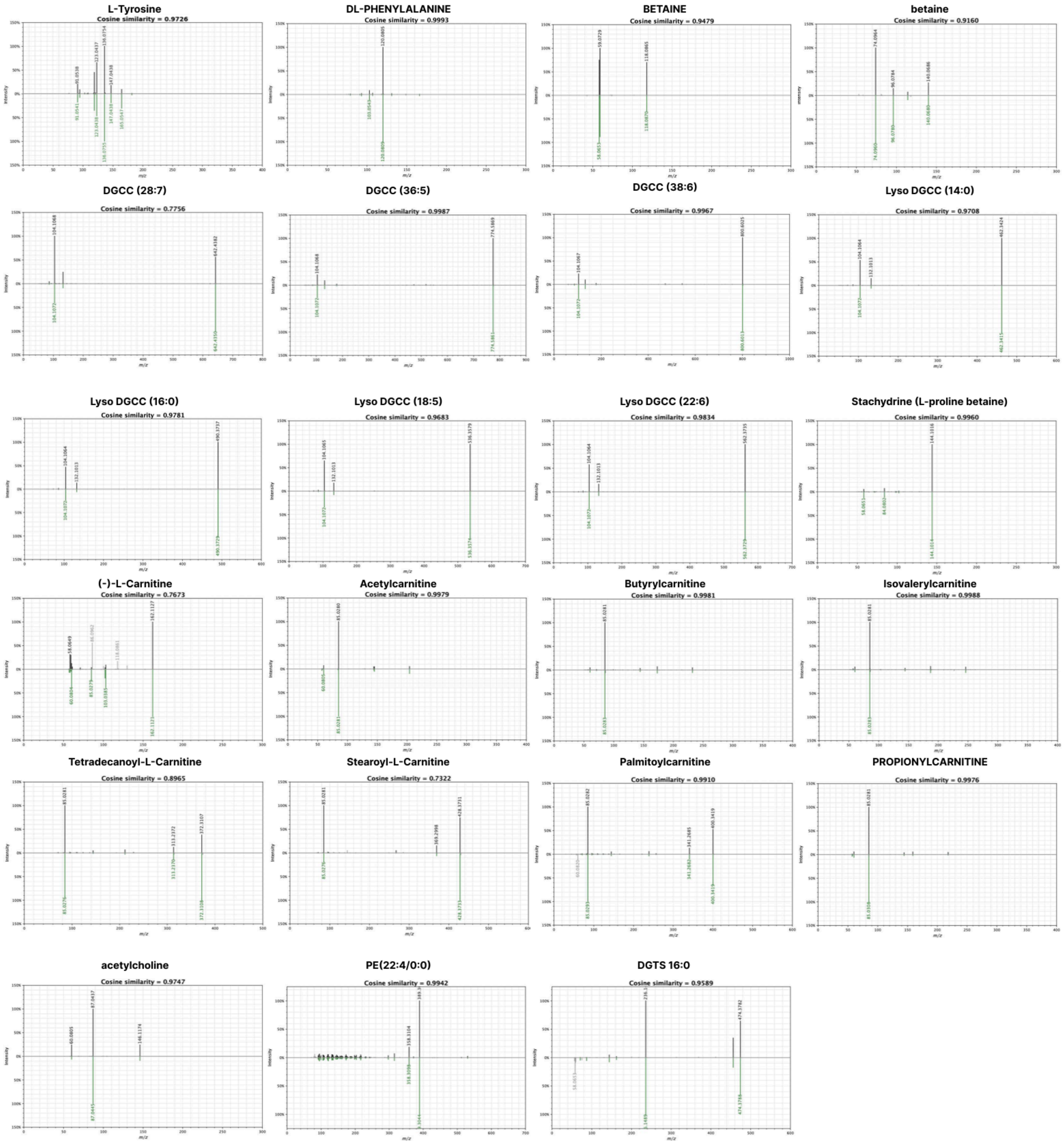

Figure S8. Mirror plots comparing experimental spectra (top) to library spectra (bottom). Cosine scores indicate peak alignment within each mirror plot.

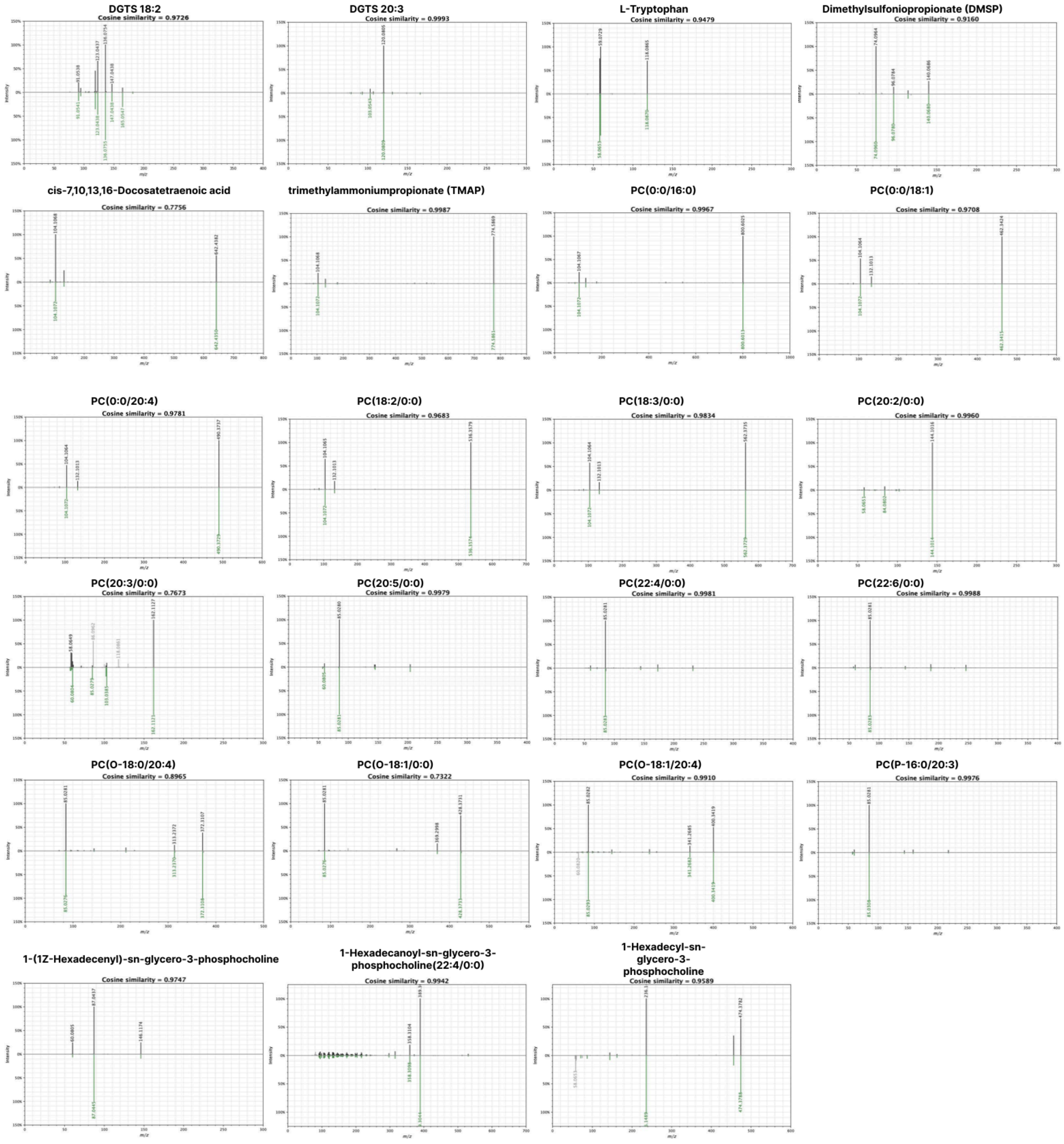

Figure S8-continued.

Table S1. Number of coral tissue samples per site (biological replicates)

Site	*Montipora* biological replicates	*Porites* biological replicates
1	11	12
2	12	12
3	12	12
4	11	12
5	12	12
6	12	12
7	11	12
8	12	12
9	11	12
10	14	15
11	12	12
12	10	11
13	12	12
14	12	12
15	11	12
16	11	11

Table S2. Feature and molecular families/single-loop nodes (SLN) remaining after each step in the informatic pipeline

	Raw		Background Removal		Rare filter		Linear mixed model	
Species	Features	Molecular families/SLN	Features	Molecular families/SLN	Features	Molecular families/SLN	Features	Molecular families/SLN
Montipora capitata	2142	615	1426	400	966	266	554	242
Porites lobata	2142	615	1315	386	786	240	733	189

References:

1. Dubininkas, V. (2017). Effects of substratum on the growth and survivorship of Montipora capitata and Porites lobata transplants. Journal of experimental marine biology and ecology, 486, 134-139.

2. Gondo, T. F., Huang, F., Marungruang, N., Heyman-Lindén, L., & Turner, C. (2024). Investigating the quality of extraction and quantification of bioactive compounds in berries through liquid chromatography and multivariate curve resolution. Analytical and Bioanalytical Chemistry, 416(24), 5387-5400. doi:10.1007/s00216-024-05474-8.

3. Harvey, A.L., (2008). Natural products in drug discovery. *Drug discovery today,* 13(19-20): 894-901.

4. Kalbitzer, U., & Heistermann, M. (2013). Long-term storage effects in steroid metabolite extracts from baboon (Papio sp.) faeces – a comparison of three commonly applied storage methods. Methods in Ecology and Evolution, 4(5), 493-500. doi:https://doi.org/10.1111/2041-210X.12054

5. Petrick, L. M., Niedzwiecki, M. M., Dolios, G., Guan, H., Tu, P., Wright, R. O., & Wright, R. J. (2024). Effects of storage temperature and time on metabolite profiles measured in dried blood spots, dried blood microsamplers, and plasma. Sci Total Environ, 912, 169383. doi:10.1016/j.scitotenv.2023.169383

6. Sitnikov, D. G., Monnin, C. S., & Vuckovic, D. (2016). Systematic Assessment of Seven Solvent and Solid-Phase Extraction Methods for Metabolomics Analysis of Human Plasma by LC-MS. Scientific Reports, 6(1), 38885. doi:10.1038/srep38885

7. Wang, M., Carver, J. J., Phelan, V. V., Sanchez, L. M., Garg, N., Peng, Y., . . . Bandeira, N. (2016). Sharing and community curation of mass spectrometry data with Global Natural Products Social Molecular Networking. Nature Biotechnology, 34(8), 828-837. doi:10.1038/nbt.3597
