# Supplementary material for: Coral metabolome quality and contaminant loads track human land use
Source: Nat Commun. 2026 Jul 15;17:6034. doi: 10.1038/s41467-026-74960-7 (PMC13373168; doi:10.1038/s41467-026-74960-7)
Supplement: Supplementary file 3 — Description of Additional Supplementary Files [file 41467_2026_74960_MOESM3_ESM.pdf]

Filename: Supplementary Data 1

Description: Statistical results from, references for spectral library match table and parameters for mzMine and SIRIUS 4.

Tab: Coral Cover

Description: Statistical results from the linear models of coral cover through time

Tab: Site Linear Mixed Model

Description: Multiple linear regression models were used to evaluate to what degreee environmental parameters could predict different aspects of coral metabolomes (dependent variables). This table is the summary of those statistics

Tab: Regression Model Statistics

Description: Statistical results from estimation of marginal means tests following linear mixed models for tests of stoichiometry, shannon diveristy, and metabolite source versus metabolite site clusters.

Tab: emmeans tests vs clusters

Description: Spectral library matches within the dataset with their sources annotated, including citations for each source.

Tab: Spectral library matches

Description: Spectral library matches within the dataset with their sources annotated, including citations for each source.

Tab: References

Description: References for the Spectral library match table

Tab: MzMine parameters

Description: All parameters used for feature finding in Mzmine

Tab: SIRIUS parameters

Description: All parameters used for molecular formala and structure prediction in SIRIUS 4
