# Supplementary material for: Coral metabolome quality and contaminant loads track human land use
Source: Nat Commun. 2026 Jul 15;17:6034. doi: 10.1038/s41467-026-74960-7 (PMC13373168; doi:10.1038/s41467-026-74960-7)
Supplement: Supplementary file 5 — Reporting Summary [file 41467_2026_74960_MOESM5_ESM.pdf]

nature portfolio

Corresponding author(s): Zachary A. Quinlan, Zquinlan@gmail.com

Last updated by author(s): 5/22/2026

Reporting Summary

Nature Portfolio wishes to improve the reproducibility of the work that we publish. This form provides structure for consistency and transparency in reporting. For further information on Nature Portfolio policies, see our Editorial Policies and the Editorial Policy Checklist.

Statistics

For all statistical analyses, confirm that the following items are present in the figure legend, table legend, main text, or Methods section.

n/a	Confirmed	
☐	☒	The exact sample size (*n*) for each experimental group/condition, given as a discrete number and unit of measurement
☐	☒	A statement on whether measurements were taken from distinct samples or whether the same sample was measured repeatedly
☐	☒	The statistical test(s) used AND whether they are one- or two-sided<br>*Only common tests should be described solely by name; describe more complex techniques in the Methods section.*
☐	☒	A description of all covariates tested
☐	☒	A description of any assumptions or corrections, such as tests of normality and adjustment for multiple comparisons
☐	☒	A full description of the statistical parameters including central tendency (e.g. means) or other basic estimates (e.g. regression coefficient) AND variation (e.g. standard deviation) or associated estimates of uncertainty (e.g. confidence intervals)
☐	☒	For null hypothesis testing, the test statistic (e.g. *F*, *t*, *r*) with confidence intervals, effect sizes, degrees of freedom and *P* value noted<br>*Give P values as exact values whenever suitable.*
☐	☒	For Bayesian analysis, information on the choice of priors and Markov chain Monte Carlo settings
☐	☒	For hierarchical and complex designs, identification of the appropriate level for tests and full reporting of outcomes
☐	☒	Estimates of effect sizes (e.g. Cohen's *d*, Pearson's *r*), indicating how they were calculated

Our web collection on statistics for biologists contains articles on many of the points above.

Software and code

Policy information about availability of computer code

Data collection	Raw MS spectra were converted to the open-source MzML format using the GNPS open-convert online platform. Peak picking, gap-filling, and pre-processing was conducted in publicly available software: MzMine 4 (v4.1.0). Molecular formula and structures were predicted using the open-source software SIRIUS 4 (v6.3.4). Molecular families (subnetworks) were generated using the free online platform GNPS. The map of Maui was produced using QGIS (v3.18)
Data analysis	Data analysis was conducted in R (v4.5.1).

For manuscripts utilizing custom algorithms or software that are central to the research but not yet described in published literature, software must be made available to editors and reviewers. We strongly encourage code deposition in a community repository (e.g. GitHub). See the Nature Portfolio guidelines for submitting code & software for further information.

Data

Policy information about availability of data

All manuscripts must include a data availability statement. This statement should provide the following information, where applicable:

- Accession codes, unique identifiers, or web links for publicly available datasets
- A description of any restrictions on data availability
- For clinical datasets or third party data, please ensure that the statement adheres to our policy

The raw MS spectra generated in this study have been deposited in the MASSIVE database under the accession code MSV000098452 [https://massive.ucsd.edu/ProteoSAFe/dataset.jsp?task=4e8bd88197fb4bba8c802a2bb5d25220]. The raw data, plots and post-processed data frames has been deposited into Github and

archived using Zenodo under the DOI: http://doi.org/10.5281/zenodo.20314872

Research involving human participants, their data, or biological material

Policy information about studies with human participants or human data. See also policy information about sex, gender (identity/presentation), and sexual orientation and race, ethnicity and racism.

Reporting on sex and gender	Not Applicable
Reporting on race, ethnicity, or other socially relevant groupings	Not Applicable
Population characteristics	Not Applicable
Recruitment	Not Applicable
Ethics oversight	Not Applicable

Note that full information on the approval of the study protocol must also be provided in the manuscript.

Field-specific reporting

Please select the one below that is the best fit for your research. If you are not sure, read the appropriate sections before making your selection.

☐ Life sciences ☐ Behavioural & social sciences ☒ Ecological, evolutionary & environmental sciences

For a reference copy of the document with all sections, see nature.com/documents/nr-reporting-summary-flat.pdf

Ecological, evolutionary & environmental sciences study design

All studies must disclose on these points even when the disclosure is negative.

Study description	This study collected fragments of approximately 186 Montipora capitata, and 193 Porites lobata colonies were collected from 16 locations across 70 km of coastline on West and South Maui. Approximately 11-15 samples were collected per sampling location. For water quality assessments at each of the 16 coral sampling locations, one 500 ml water sample was collected approximately 0.5 m above the reef using a large syringe. At the surface, 50 ml from the benthic water sample was aliquoted into amber glass vials and stored at 4˚C for later water quality analysis.
Research sample	Montipora capitata and Porites lobata were selected as our target species because of their prevalence and high cover across the Hawaiian Archipelago as well as their varied life strategies. An effort was made to standardize colony depth (mean 4 m) and colony size (mean 26 cm) across all reef sites.
Sampling strategy	Approximately 11-15 samples were collected per sampling location. These samples were collected at each location to represent the location-specific metabolomes for each coral species. Coral samples were collected from visually healthy colonies using a hammer and chisel following methods described by Greene et al. with a section of skeleton and tissue 2 cm2 placed in 15 ml of HPLC grade methanol (Sigma Aldrich) 5.17 µmol L-1 2-aminoanthracene (Sigma Aldrich) on ice for metabolite extraction and transferred to a freezer as soon as possible at -20˚ C. No sample size calculations were performed as these calculations are not reliable for natural systems. While many studies may have chosen smaller sample sizes, we chose to collect samples from 11-15 corals at each site to capture a wider variety of metabolomic variability as at the time this was the largest single collection of coral metabolome tissues.
Data collection	Samples were collected by Austin Greene, William Leggat, Tracy Ainsworth, Megan Donahue, Jamie Caldwell, and Scott Heron. Coral fragments were standardized to 2 cm^2 using caliper estimates and placed in methanol to reduce enzymatic degradation of metabolites during storage. Metabolite ions were analyzed on an Vanquish Horizon UHPLC system (Thermo Scientific) by Brunda Nijagal and Metabolomics Australia. Historic percent coral cover data for Sites 1, 3, 8, 11, and 15 were retrieved from the Coral Reef Assessment and Monitoring Program (CRAMP). At each of the 16 coral sampling locations one 500 ml water sample was collected approximately 0.5 m above the reef using a large syringe. At the surface, 50 ml from the benthic water sample was aliquoted into amber glass vials and stored at 4˚C for later water quality analysis.
Timing and spatial scale	All samples were collected in March of 2018.
Data exclusions	Three samples were excluded from the dataset because of clear signs of contamination.
Reproducibility	This study has not been repeated. To guarantee this study was repeatable, we collected large sample sizes from each location to make sure we were not capturing small communities which were not representative of the location overall. We also made sure to standardize all measurements and sampling strategies.
Randomization	Samples were allocated into groups based on the sampling location and transect from which they were sampled.
Blinding	There was no a-priori designation of anthropogenic impact for each site. Initially, all metabolomics data was analyzed prior to any analysis of water quality or land-use data was assessed. Trends in metabolomic influence were described prior to the latter analysis.

Did the study involve field work? ☒ Yes ☐ No

Field work, collection and transport

Field conditions	Samples were collected In March 2018 from coastal coral reefs on days without significant rainfall. Samples were collected between March 12 and 23rd, 2018.
Location	All samples were collected from the West and South shores of Maui at an average depth of 4m.
Access & import/export	Sample collection and exchange permitting was provided by the Hawaii Department of Land and Natural Resources under SAP 2018-03 and CITES secretariat under permit #17US86408A/9. .
Disturbance	The fragments taken from corals were standardized to small sections and only taken from corals that were approximately 26 cm.

Reporting for specific materials, systems and methods

We require information from authors about some types of materials, experimental systems and methods used in many studies. Here, indicate whether each material, system or method listed is relevant to your study. If you are not sure if a list item applies to your research, read the appropriate section before selecting a response.

Materials & experimental systems

n/a	Involved in the study	
☒	☐	Antibodies
☒	☐	Eukaryotic cell lines
☒	☐	Palaeontology and archaeology
☐	☒	Animals and other organisms
☒	☐	Clinical data
☒	☐	Dual use research of concern
☒	☐	Plants

Methods

n/a	Involved in the study	
☒	☐	ChIP-seq
☒	☐	Flow cytometry
☒	☐	MRI-based neuroimaging

Animals and other research organisms

Policy information about studies involving animals; ARRIVE guidelines recommended for reporting animal research, and Sex and Gender in Research

Laboratory animals	Not applicable.
Wild animals	4 cm^2 fragments were collected from Montipora capitata and Porites lobata that were approximately 26 cm using hammer and chisel. These samples were transported to the surface and immediately separated into equal 2 cm^2 fragments. One fragment was used for metabolomics within this study and the second fragment was used for 16S amplicon work within a separate manuscript.
Reporting on sex	It is unclear what the sex of each coral colony was as this can only be determined during spawning.
Field-collected samples	Not applicable
Ethics oversight	No ethical approval was necessary for this study as it was conducted on coral which are invertebrates.

Note that full information on the approval of the study protocol must also be provided in the manuscript.

Plants

Seed stocks	Not Applicable
Novel plant genotypes	Not Applicable
Authentication	Not Applicable
