# Supplementary material for: Coral metabolome quality and contaminant loads track human land use
Source: Nat Commun. 2026 Jul 15;17:6034. doi: 10.1038/s41467-026-74960-7 (PMC13373168; doi:10.1038/s41467-026-74960-7)
Supplement: Supplementary file 2 — Peer Review file [file 41467_2026_74960_MOESM2_ESM.pdf]

Peer Review File

Coral metabolome quality and contaminant loads track human land use

Corresponding Author: Dr Zachary Quinlan

This file contains all reviewer reports in order by version, followed by all author rebuttals in order by version.

Version 0:

Reviewer comments:

Reviewer #1

(Remarks to the Author)
This manuscript describes the analysis of coral samples collected across several sites in Hawaii and analyzed for metabolomic content. While the overall project is very important to understand the impact of human activity to the coral environment, there are several key methodological issues with the project that cause concern when interpreting the results. Figure 1 is nearly impossible to read with the high number of metabolite classes depicted. I had to expand to over 400% in order to read the text. It is very unusual to use this narrow classification for metabolites to find clusters of metabolites that are similar from the different corals. The averages are used here so the variability between corals is not included thus the significance of each metabolite in terms of a specific statistical test is not described.
The authors stated that 12-15 samples were collected per sampling location. Does this infer that those samples were collected from different regions of the coral reef in that area?
On page 16 line 286, the authors state that 16 locations were used, but on line 292, they refer to 20 coral sampling locations. Can the authors clarify how many locations were used for sampling?
Samples were taken and then immediately put into 15 mL of methanol and frozen, but the temperature used for freezing is not included. Since it was about 1 year before the samples were analyzed, this is a critical piece of information as anything under -80C will likely have significant degradation associated with metabolite expression. While the authors note that this is an issue, it does not mean that it should be overlooked as part of a complication for research. If the metabolite identified is due to degradation rather than anthropogenic activity, then the results could be invalid or could lead to the erroneous identification of the source of the metabolite. In addition, there is no mention to how the samples were normalized for metabolomics and this could be a very important factor. Sampling using a hammer and chisel will provide an inconsistent amount of material per sample and thus an inconsistent extraction for each. Is it possible that coral reefs in areas with anthropogenic impact are easy to chisel and thus provided more material? If so, this would offset the overall results. With a methanol extract, it might be possible to pellet the protein and resolubilize that pellet to get an overall protein amount. Samples could then be normalized to protein content unless that protein content is significantly different between the sites.
For the LC-HRMS method, a 200 uL aliquot was provided to Metabolomics Australia. Was this aliquot used without further processing? It is atypical to use methanol as the injection solvent for an LC-HRMS method as it can cause inconsistency during injection. Often this sample would be dried down and reconstituted in a more compatible injection solvent for the reversed phase method used. Can the authors clarify this aspect? Why was negative ion analysis not collected? This will miss many key metabolites.
The methanolic extracts were spiked with 2-aminoantracene, but the authors do not state how much was spiked in and what concentration was spiked. In addition, the authors should expand as to why this was added.
Figure 4 is very difficult to read and require increase to size to 200% in order to review and evaluate it. Reviewing panel B, the authors have identified both PCs and PAFs (and lyso versions). However, the authors are likely aware that these are isomeric and thus would have to be separated via LC in order to distinguish them from each other. Did the authors verify if separation was achieved to distinguish them? Note that a simple searching using GNPS or other database would identify them because MS/MS is identical. They also list generic names for Lyso-PAF and Lyso-PC and PC. These should be listed as the specific species that was measured instead of the overall class since in this section, they are referring to specific metabolites. In panel C, they identified Adenosine, which is an important nucleoside. Due to their extraction conditions, the identification of this could be due to degradation of ATP, ADP and AMP. Also, they label adenosine as pharmaceutical in origin which is incorrect. Overall, this gives little confidence that the identified metabolites are indeed correct.
Why were the water samples not used to analyze for agricultural products in addition to the water quality? This seems like a missed opportunity as it could be used to evaluate contamination on the day of collection.

Another key concern is whether a classical ANOVA was conducted based on site and metabolite rather than summed metabolite based on subclass? It is unclear as to the overall variability of the sampling approach and methods used as to whether individual metabolites would be identified as significant. This would be important to clarify.

(Remarks on code availability)

Reviewer #2

(Remarks to the Author)
I co-reviewed this manuscript with one of the reviewers who provided the listed reports

(Remarks on code availability)
I co-reviewed this manuscript with one of the reviewers who provided the listed reports

Reviewer #3

(Remarks to the Author)
Summary
The manuscript describes the exploration of the relationship between the metabolome of two coral species, including accumulated anthropogenic chemicals, and human land use in Hawaii. The collected corals are grouped into three main clusters based on the detected metabolome, and those clusters are assigned anthropogenic influence levels based on adjacent land use and water quality parameters (i.e., nutrient concentrations). The metabolomes of the clusters show different levels of chemical diversity, carbon oxidation state, and nutrient ratios. Specifically, they also show differences in the chemical composition or relative abundances of coral metabolites, stress metabolites, and anthropogenic chemicals. The authors conclude that the metabolic signatures of the investigated coral tissues represent the level of human land use. The research question is highly relevant, the study design is warranted, and the paper presents an interesting and valuable dataset. However, the authors seem to overstate the implications of their findings and have based their conclusion on sometimes unsupported or unfit arguments and statistical analyses.
The paper deals with a relevant and emerging topic: the impacts of anthropogenic activities, through the release of chemical pollutants, on corals and coral reef ecosystems. Since the collected dataset and its implications are potentially of great value, I support its publication in a high-quality platform like Nature Communications. However, given the serious shortcomings in the data analysis and interpretation, the manuscript requires substantial revisions before it can be considered for publication. I appreciate the work put into this manuscript by the authors, and hope the provided comments will help them improve the manuscript.

Milo L. de Baat, Ph.D.

General comments
1) l. 36-37. "…the bioaccumulation of pollutants in corals impacts all but the most remote reef ecosystems." Did the study actually demonstrate the impact of the accumulated chemicals on coral health? Although this relationship is likely, the study only showed a positive relationship between the biotic concentrations of anthropogenic chemicals and the intensity of human land use, rather than providing proof of a causal relationship between those concentrations and reduced coral health, for example, through toxicity.

2) l. 37. "…which will disproportionately threaten communities reliant on reefs." Do the authors mean biological communities, human communities, or both? And why disproportionally? If anything, I would say the reef-dependent communities are threatened proportionally to the level of anthropogenic activities nearby. And did the authors compare the impacts of the bioaccumulation of chemicals between reef-dependent and independent communities?

3) The metabolite clusters were "classified as less, moderately or highly influenced by human activities based on the water quality and the onshore habitat disturbance." The metabolic clusters were defined by hierarchical clustering of the relative abundances of nontarget features. I have two concerns regarding this approach:
a) To do this, the relative abundances of metabolites within samples were used. However, these relative abundances are not directly related to actual concentrations in the coral tissues, since nontargeted analyses are at best semi-quantitative. Moreover, since they are relative within samples, they may not be comparable between samples. Additionally, they were grouped into subnetworks/single-loop nodes, making a quantitative comparison and thus grouping based on metabolite abundances problematic, since we don't know what the composition and relative and absolute abundances of the individual features within these subnetworks were, and whether they were comparable between samples. I understand the authors are looking for an approach to make untargeted metabolomics data manageable and interpretable, but I worry that this approach may oversimplify the complexity of the situation in the field. I am curious how the authors see this, and whether they can come up with a more quantitative way of comparing their samples in terms of chemical diversity. An approach visualizing the overlap in individual features between samples, for example, using a Venn diagram, may better represent actual differences between sites. Additionally, a multivariate analytical tool such as NMDS and subsequent analysis of similarity may help group sites based on the actual composition of metabolites. Multilevel pattern analysis may then identify particular (groups of) features associated with those site clusters.
b) It seems that the qualification of the clusters as low, medium, or high anthropogenic metabolome influence was done on a

purely qualitative basis. Although that classification may seem intuitive, the values for water quality and the onshore habitat disturbance represented in the heat map in Fig. 1 do not seem to allow such classifications. For example, there are locations in clusters 1 and 2 that have a relatively high level of watershed disturbance. Moreover, the water quality parameters are also not uniformly distinct for each of the clusters, with relatively high and low values distributed over the clusters. Is it correct that these classifications were performed by the authors and were not quantitatively determined based on the numerical values of the parameters? In that case, I strongly suggest refraining from such a bias-prone approach and applying a continuous numerical disturbance score based on measured values.

4) The extraction of metabolites from the coral tissue used solely methanol as the extraction solvent. However, given the focus of the study on metabolites in tissue, a more nonpolar extraction solvent may have improved the extraction of the lipophilic chemicals that generally accumulate more strongly in biota. Why did the authors choose to extract only in methanol? Are any data available on the extraction efficiencies of this method?

5) The authors use the nitrogen/phosphorus content to draw conclusions on the nutrient availability in the field, and the nominal oxidation state of carbon to derive the "energetic availability of the metabolome." What is the energetic availability? And can such conclusions be drawn from these data? Is previous research available that demonstrates these correlations? Moreover, these values were, again, based on the relative abundance of the metabolites, which are not directly related to concentrations. Hence, it seems premature to draw conclusions on nutrient availability and energetic availability from these values.

6) L. 153-154 & 164-168. Tukey's test is not a correlation analysis. It is a post-hoc test after an ANOVA that can indicate significant differences between treatments. A correlation analysis would require a continuous numerical value for the disturbance level and a different statistical test. See also my general comment 3b.

7) L. 177-178. I understand the argument here, but is this metabolome represented by small organic molecules really the main energy reserve of corals? I assume that would be much better represented by larger lipids and complex DOM molecules, and labile energy sources such as glucose and ATP. I doubt whether these molecules are adequately preserved and extracted using the applied methanol protocol, and they also do not seem amenable to the applied LC-MS method.

8) L. 212-216. I find the drawn conclusions here too strong based on the presented data. Especially the "increased stress responses", based only on the enrichment of DMSP, seem too readily concluded. In Figure 4B, the proportional DMSP enrichment seems rather equally distributed across the clusters, based on the little pie chart. Can a generalized increased stress really be concluded based on these data?

9) Figure 4. What is the identification level of these chemical features? The authors present these features as fully identified, but no measure is given, for example, as Schymanski scores, of the certainty of these identifications. If these are only tentative identifications, the conclusions may certainly be too strong (see general comment 8).

10) The discussion contains very few references to the relevant scientific literature. As such, the findings are not properly placed in the context of the current state-of-the-art. For example, in l. 251-252, the authors state that anthropogenic chemicals accumulate with untold physiological consequences. Indeed, too little is known about the toxic effects of anthropogenic chemicals on coral health. However, multiple studies have demonstrated such effects, while the authors did not seem to try to link detected chemicals to potential negative outcomes in corals. The authors are strongly recommended to consult the review paper by Ouedraogo et al. (2023) as well as more recent publications to link detected chemicals with potential ecotoxicological effects in corals. This should also help revise the provided interpretation of the available literature in l. 259-264.

11) L. 280-282. I agree that the presented approach to coral reef impact monitoring is novel. However, monitoring of hydrophobic anthropogenic chemicals on coral reefs could also be performed using sediment samples, which is much less invasive and does not carry the downsides of working with often endangered coral species. Although it does not provide the same information as biotic sampling, it could be an accessible and low-impact way of monitoring anthropogenic chemical pollution on coral reefs. Moreover, the authors applied chemical analytical methods that are generally more fit for detecting small molecules in the more polar spectrum. These methods seem more applicable when monitoring the water phase rather than biota or sediments. Ideally, water quality assessment on coral reefs would include anthropogenic chemical monitoring of water, sediment, and biota. The authors are recommended to include a reflection on these points, supported by the appropriate scientific literature, in their discussion.

12) Were seawater blanks for each site included in the chemical analyses? Detected metabolomes should be corrected for the local seawater background to allow conclusions on the bioaccumulation of anthropogenic chemicals. If this was not done, how can the authors be sure that the detected anthropogenic chemicals were accumulated in the coral tissue, rather than simply being present in the surrounding water?

13) L. 397-421. I assume that the "statistical analyses" section will be amended based on the provided general and specific comments above and below. Hence, I will not provide the same feedback again in this section.

Specific comments
L. 1. The word "metabolism" in the title suggests that the authors studied the metabolism of the corals. Although the paper focuses on the metabolome, I would argue that they did not measure the metabolism of the studied corals.

L. 1. The word “track” in the title suggests active tracking by the corals. I understand why the authors chose this term, but it seems more correct to use a more passive term like follow or trace.
L. 48. “…poses a substantial threat…”
l. 49-50. Why particularly CECs? I would argue that exposure to mixtures of all pollutants, regardless of whether they are legacy or emerging contaminants, is potentially harmful to corals. The cited references mostly focus on legacy pollutants, highlighting a knowledge gap on the impacts of CECs on coral health.
l. 56-57. This is a repetition of l. 50-52. I suggest removing the first mention because it will result in a better line of argument while maintaining the specific mention of water quality as a driver of coral reef health.
l. 66. “was” = were.
l. 66. Remove “directly”.
l. 69-70. “…were additionally accumulating…” = additionally accumulated.
l. 71. Add a comma behind “reserves”.
l. 72-75. Suggest changing to: These results suggest that corals use a portion of their energy reserves to respond to the stress levels imposed by local human activities, such as land-derived pollution, reducing their resilience by depleting the ability of these corals to respond to global stressors, such as increased sea surface temperature.
l. 82. “…features respectively…”.
l. 88. “through” = of
l. 89. Not only agricultural nutrient pollution, but also pesticides, especially in light of the manuscript’s focus on anthropogenic chemical pollution.
l. 88-91. Most anthropogenic contaminants find their way to the reefs through runoff, alongside other pollutants from tire wear and other road uses, untreated wastewater, and pesticide use, but this is not specifically mentioned, while subsurface discharge is mentioned. Please add this.
L. 103-104. How were they correlated? Was this statistically tested?
l. 105. “…wherein the proportional” – such grammatical errors of leaving out the definite article occur throughout the manuscript. The authors are advised to do a final grammatical check of the whole manuscript.
l. 107. “greater” = more
l. 112-115. See general comment 3b.
l. 117. “experienced” = were part of
l. 120. “from” = observed at
l. 120. However, there is more information in these trends. For example, how do the authors explain that, according to their classification, the most heavily impacted monitored site was the only site that showed an increase in coral cover over the years preceding the bleaching?
l. 126-127. Remove “…along with…”
l. 147-148. How is the diversity of metabolites indicative of metabolome energy reserves?
l. 156 & 168. From which analyses are these explained variances derived?
L. 220. Is it really the reduced energy reserves that drive the susceptibility of these corals to additional stressors? Could it be something else? There seems to be a lot of room for confounding effects of other stressors.
l. 226-227. But octocrylene is not listed as an anthropogenic chemical detected in the present study. Are toxic effects of the detected chemicals to coral described in the scientific literature? The review by Ouedraogo et al. (2023) may provide insights here.
Fig. 4C. DEET is a common personal care product rather than an agricultural chemical.
l. 257. Why “known”? Aren’t all the identified features known chemicals?
l. 265. Which management practices are currently being applied in the studied area?
l. 269. Although novel, anthropogenic chemical pollution is not a previously unrecognized threat to coral reef ecosystems. See, for example, the papers by Ouedraogo et al. (2023), Nalley et al. (2021), and De Jong et al. (2025).
L. 275. I agree that corals are potentially susceptible to bioaccumulation. However, why do the authors think those chemicals would otherwise break down in the environment, and not in corals? Corals have detoxification mechanisms and a rich microbiome, after all. Moreover, some of these chemicals are very persistent and resistant to (microbial) degradation. I would rather argue that the mixotrophic nature of the coral holobiont, alongside their sessile life history, makes them more susceptible to bioaccumulation since they accumulate chemicals through a variety of pathways.
L. 279. See general comment 2.
L. 394. Porites lobata, not capitata

(Remarks on code availability)
I did not install and run the code, but checked the provided documents and README. All files were accessible, and together with the README file, allow reproduction of the data analyses.

Version 1:

Reviewer comments:

Reviewer #1

(Remarks to the Author)
Overall, the authors did a good job in responding to this reviewers comments. While I am still concerned with samples stored at -20C in methanol, as along as the authors note this as a potential issue in their manuscript, then it is acceptable.

(Remarks on code availability)

Reviewer #2

(Remarks to the Author)

(Remarks on code availability)

Reviewer #3

(Remarks to the Author)
The authors have carefully reviewed the manuscript based on both reviewers' comments and revised sections accordingly. I appreciate the informed discussion that this gives rise to, and believe it has strongly improved the manuscript. It is now more fit for publication, but some minor concerns remain that should be addressed. I have listed those below:

- In response to my comment 3a, the authors explain their approach to standardizing the detected feature intensities. This is now sufficiently addressed. However, rather than using the term "relativized", I suggest the authors use the more common and appropriate term "normalized" or "standardized".
- I appreciate the explanation given for my comment 3b. However, based on the response, it seemed that the authors indeed assigned sites based on a qualitative measure of anthropogenic influence. My point was that this should be based on quantitative data, because the current approach appears to introduce a bias. Since subsequent data analyses are performed on these groups, the grouping is of great influence on the outcomes of the study, and should be performed purely based on numerical values and statistics. Either I haven't fully understood the quantitative way in which the clusters were assigned, suggesting that this requires better explanation in the manuscript, or this was indeed assigned based on expert opinion. In the latter case, I invite the authors to come up with a fully unbiased and quantitative approach to the clustering.
- For comment 4, it may be true that MeOH is a common solvent for untargeted metabolomics in corals, and I understand the value of that. However, in the field of biomonitoring of anthropogenic chemicals, it is common to use (mixtures of) solvents that efficiently extract both polar and nonpolar anthropogenic chemicals from tissue samples. Any extraction method will provide only a certain fraction of the chemical universe, and that is not a problem, especially if the extraction efficiency is reported. Do the authors have any numbers on the extraction efficiencies for anthropogenic pollutants from coral tissue using this method? That would greatly help readers interpret the presented data. For example, if a common pollutant, say sucralose or caffeine, was not detected in the coral tissue, is this because it was not extracted by the selected method or because it was absent from the tissue?
- The expansion of the discussion in response to comment 11 is valuable. However, some grammatical changes to the amended text are advised: (l. 292-293) "While this does mean corals are more susceptible at risk to anthropogenic contaminants…" should be "more susceptible to" OR "more at risk from"; (l.295) "bioaccumulates" should be "the chemicals bioaccumulated in coral tissues…"
- I understand the challenges of high salt content in LC-MS analyses, given in response to comment 12. However, I disagree that taking seawater blanks is not advisable. Any environmental chemistry study should include blanks, ideally, to correct for background contamination. This is especially true for non-target analyses. Even though I understand why the authors did not include them in this study, I strongly suggest they start using seawater blanks, despite the challenges, in future projects. However, the current findings are still of value, and we can leave it at this for the present study.

(Remarks on code availability)

Reviewer #4

(Remarks to the Author)
The dataset collected here is impressive, particularly the multi-species comparison and the attempt to link metabolomic profiles to anthropogenic disturbance. The overall question is clearly relevant. That said, even after revision, I still find the definition and interpretation of the site clusters problematic, and this carries through several of the downstream analyses and ultimately affects how convincing the conclusions are.

A central issue is that the manuscript relies heavily on clusters derived from hierarchical clustering of metabolomic data, yet I was not able to find a formal statistical test showing that these clusters are actually distinct in multivariate space. The additional ordination analyses (PCA/PCoA) help to visualize the structure, but they do not resolve this point. In fact, in Porites in particular, the pattern looks more like a continuum than clearly separated groups, which makes the current interpretation difficult to reconcile with the data.

At a minimum, I think the authors need to test for group separation using PERMANOVA, based on the same distance matrix used for clustering, and report effect sizes such as R2 alongside significance. It would also be important to include a test of dispersion using PERMDISP, since differences in within-group variance can otherwise give a misleading impression of separation. As it stands, the clusters appear to be defined visually rather than statistically.

Related to this, the interpretation assumes discrete "low", "medium", and "high" impact groups, whereas the ordination suggests that a continuous gradient may underlie the data. It would help to test this explicitly, for example by asking whether

metabolomic variation is better explained by cluster membership as a categorical factor or by environmental variables treated as continuous predictors. Looking at correlations between ordination axes and measures such as land-use disturbance or contaminant load would already go a long way toward clarifying this.

I also found it difficult to fully understand how the clustering was performed. While hierarchical clustering of XIC intensities aggregated at subclass level is mentioned, key parameters are either missing or not clearly stated. In particular, I could not find a clear description of the distance metric used (Bray Curtis? Euclidean?), the linkage method, the preprocessing steps applied to the data, or how the number of clusters was chosen. Given how strongly these choices can influence the outcome, this level of detail is really needed for the analysis to be evaluated properly.

Another point that is not entirely clear to me is how the filtering of metabolites affects the downstream analyses. The manuscript states that analyses focus on metabolites that are significantly different between sites. While that is reasonable in principle, it does introduce a potential circularity, since features are selected precisely because they differ between sites, and are then used to interpret differences between clusters derived from the same data. It would therefore be helpful to clarify whether clustering itself was performed on the full dataset or on a filtered subset. As a simple robustness check, repeating the clustering on the full, unfiltered dataset would help to see how stable the structure actually is.

The classification into “low”, “medium”, and “high” impact groups also seems to be derived from the metabolomic clustering and then interpreted in relation to environmental variables. This is fine as an exploratory approach, but it should be stated explicitly. At the moment, it reads somewhat as if these categories were defined independently. It would also strengthen the manuscript to quantify these relationships more formally, rather than relying mainly on descriptive comparisons.

Finally, the fact that some sites cluster differently depending on species is mentioned, but not really explored. This made me wonder to what extent the clustering framework is consistent across taxa, and whether some of the conclusions might depend on species-specific patterns. A more explicit comparison would be useful here.

Overall, this is a valuable dataset addressing an important question, and I think the study has real potential. However, the current analysis relies on clusters that are not statistically validated and whose methodological basis is not sufficiently described. Addressing these points would make the conclusions much more robust.

(Remarks on code availability)

Version 2:

Reviewer comments:

Reviewer #4

(Remarks to the Author)
Thank you for the thorough revision. The manuscript is clearly stronger than the previous version, and I appreciate the serious and constructive way the authors responded to the earlier comments.

The additional detail on how the clustering was performed is useful, and the inclusion of the PERMANOVA and PERMDISP analyses addresses an important gap in the original submission. I also found the added robustness check using the full metabolome dataset reassuring, as well as the expanded discussion of the species-specific patterns.

My only remaining point is more about interpretation than analysis. Even though the clustering framework is now much better supported, some of the ordination patterns, particularly for Porites, still read to me more as a gradient than sharply separated groups. I would therefore encourage the authors to keep some restraint in describing these categories as fully discrete states, and to note that continuous environmental variation is also likely contributing to the observed structure.

Overall, I think the main concerns have been dealt with satisfactorily. This is an interesting and timely study based on a substantial dataset, and in my view the manuscript is now suitable for publication.

(Remarks on code availability)

Open Access This Peer Review File is licensed under a Creative Commons Attribution 4.0 International License, which permits use, sharing, adaptation, distribution and reproduction in any medium or format, as long as you give appropriate credit to the original author(s) and the source, provide a link to the Creative Commons license, and indicate if changes were made.
In cases where reviewers are anonymous, credit should be given to 'Anonymous Referee' and the source.
The images or other third party material in this Peer Review File are included in the article's Creative Commons license, unless indicated otherwise in a credit line to the material. If material is not included in the article's Creative Commons license and your intended use is not permitted by statutory regulation or exceeds the permitted use, you will need to obtain permission directly from the copyright holder.
To view a copy of this license, visit https://creativecommons.org/licenses/by/4.0/

Reviewer 1

This manuscript describes the analysis of coral samples collected across several sites in Hawaii and analyzed for metabolomic content. While the overall project is very important to understand the impact of human activity to the coral environment, there are several key methodological issues with the project that cause concern when interpreting the results.

Thank you for your time and effort in reviewing this manuscript. We believe the points which this reviewer has brought up are very helpful and have done our utmost to address and respond to each individual comment below. We believe the comments have made this manuscript stronger. Thank you so much!

Figure 1 is nearly impossible to read with the high number of metabolite classes depicted. I had to expand to over 400% in order to read the text. It is very unusual to use this narrow classification for metabolites to find clusters of metabolites that are similar from the different corals. The averages are used here so the variability between corals is not included thus the significance of each metabolite in terms of a specific statistical test is not described.

Thank you for bringing up this point of confusion. The underlying statistics that were used before any of this was analyzed was a linear mixed model (with the feature number as the random effect) to evaluate whether the entire molecular family (molecular subnetworks as defined by feature-based molecular networking; Nothias et al., 2020; Nguyen et al., 2013) were enriched differently between locations. We have found that this can help to identify enrichment more effectively than a similar one-way ANOVA would if just using individual ion features to test for significance. The linear mixed model helps to identify molecular families enrichment more readily as it is less biased by 0’s, noise, and differences in ionization of individual features within the molecular family. A similar analysis was suggested and utilized in Quinlan et al., 2025 and Quinlan et al., 2023.

Originally, we used the sum chemical classification in an effort to better summarize chemical differentiation and enrichment between locations. e.g. some molecular families are classified the same and this reduces the total number of columns in the dendrogram while still conveying the same message. However, seeing how it has actually caused more confusion instead of less, we have changed Figure 1 to be built upon the total relative abundance of each molecular family instead of the total relative abundance of each chemical classification. This demonstrated the same clustering of sites; indeed we now show in Figure S2 that regardless of the level of characterization (feature, molecular family, or *in silico* predicted annotation), our site classification remains robust. Please see Figure 1 and S2 below.

Additionally on line 404 we have added the text “To identify molecular families that vary between our sampling sites we tested the effect of site number on subnetwork abundance with metabolite number as a random effect using a linear mixed model (subnetwork abundance ~ siteNumber + (1|metabolite)). All metabolites and molecular families presented herein were limited to those that were significantly different between sites as defined from the linear mixed model (false discovery rate corrected LMER p-value < 0.05)”.

Figure 1:

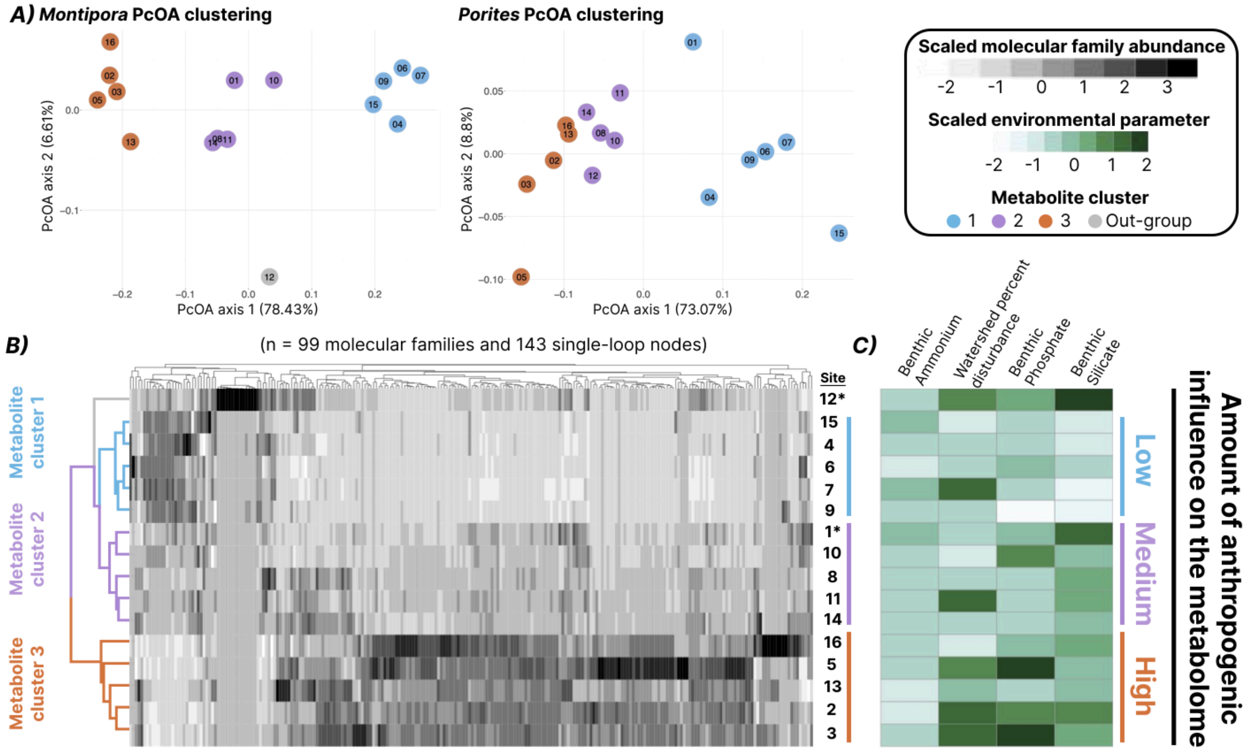

Figure S2:

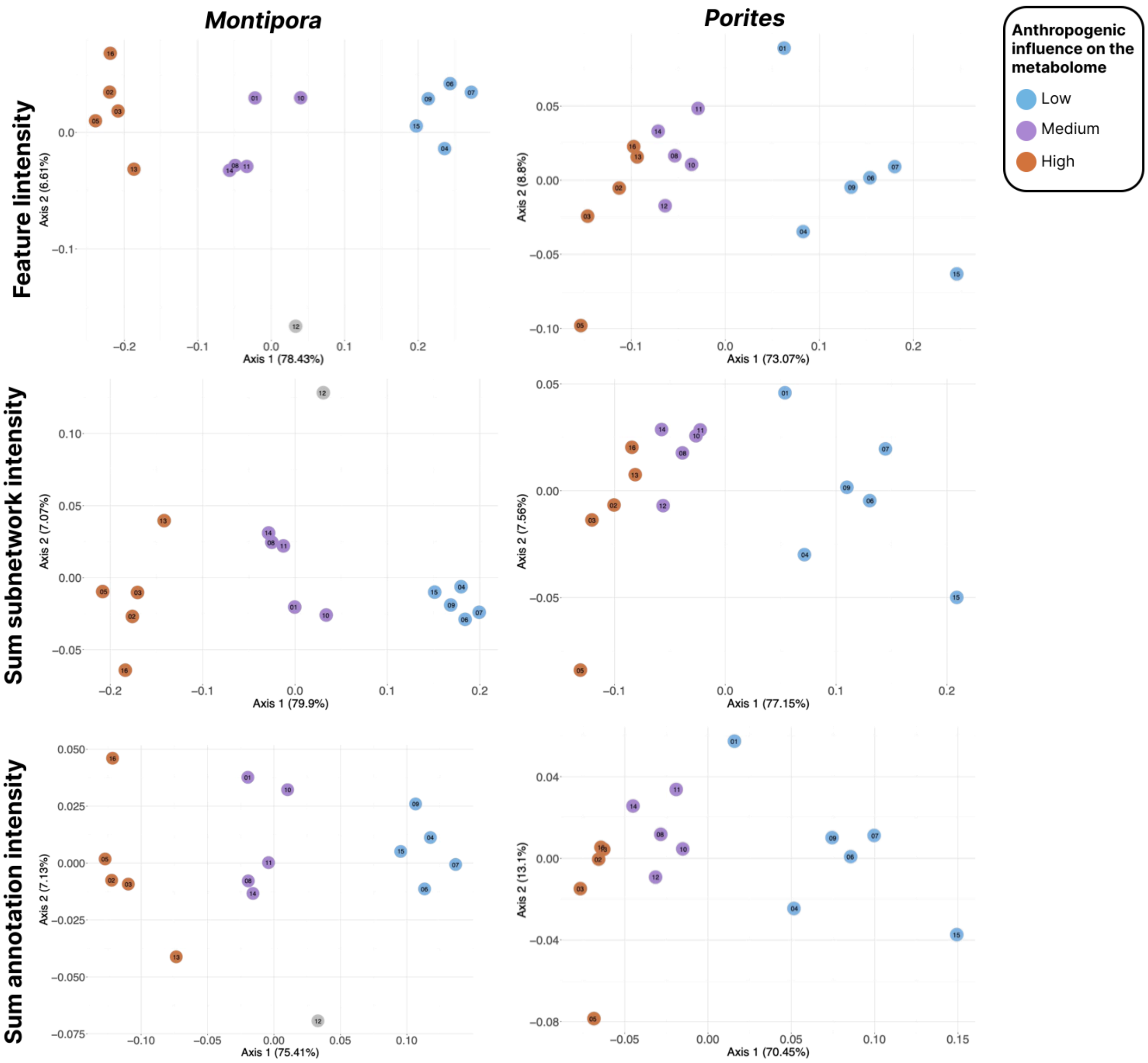

The authors stated that 12-15 samples were collected per sampling location. Does this infer that those samples were collected from different regions of the coral reef in that area?

Yes, the 12-15 samples at each location were all from separate corals across that individual reef. We have added more information about the sampling scheme to line 292 to explain this: “Colonies were sampled across transect lines alongside TNC surveys of the same reefs. Colonies along each transect were only sampled if they were visually healthy without any signs of disease.“

On page 16 line 286, the authors state that 16 locations were used, but on line 292, they refer to 20 coral sampling locations. Can the authors clarify how many locations were used for sampling?

There were 16 sites sampled for metabolites. In total during these surveys 20 sites were sampled by TNC for water quality metrics. Thank you for catching this error, we have changed any mention of 20 sites to 16 as those are the only ones with metabolome information.

Samples were taken and then immediately put into 15 mL of methanol and frozen, but the temperature used for freezing is not included. Since it was about 1 year before the samples were analyzed, this is a critical piece of information as anything under -80C will likely have significant degradation associated with metabolite expression. While the authors note that this is an issue, it does not mean that it should be overlooked as part of a complication for research. If the metabolite identified is due to degradation rather than anthropogenic activity, then the results could be invalid or could lead to the erroneous identification of the source of the metabolite.

We agree and have now included in the methods that all samples were stored and frozen at -20˚ C prior to analysis. We have added a deeper discussion of this in the supplemental discussion and referenced this discussion in the main text on line 321 “A further discussion of freezing length and temperature is available in the supplemental discussion”.

supplemental discussion: “The methanol extracts from our samples were stored at -20˚ C for one year. Best practices for untargeted metabolomics, suggest freezing at -80˚ C reduces the possibility of metabolite degradation. This could potentially risk the stability of some molecules however it is unlikely to bias our conclusions because: 1) All samples were stored together and since we only compare across sites any degradation would be consistent across all samples, 2) All samples were stored in Methanol with reduces the enzymatic activity and thus degradation of metabolites, 3) To our knowledge there have been no studies that test metabolite stability in methanol and/or ethanol at -20˚ C compared to -80˚ C.

For metabolomics, methanol is widely considered the best storage solvent because it quenches enzymatic activity increasing metabolite stability and its broad metabolite coverage (Sitnikov et al., 2016). Although there is still a risk that we may have observed more anthropogenic metabolites if our samples were stored at -80˚ C instead of -20˚ C, the decreased enzymatic activity within methanolic extracts and relative stability at -20˚ C observed in other sample types suggests there would be minimal degradation over 12 months. While the stability of metabolites in no extraction solvent over extended storage times (Petrick et al., 2024; Kalbitzer, and Heistermann)

or at higher temperatures (Petrick et al., 2024; Kalbitzer, and Heistermann 2013), and stability across different extraction solvents (Kalbitzer, and Heistermann 2013) have all been tested separately, there have been no direct tests of metabolite stability in methanol and/or ethanol at -20˚ C compared to -80˚ C. Petrick et al., (2024) demonstrated that in dried blood samples, storage at -20˚ C remained similar to those stored at -80˚ C over a 6 month period, although plasma had slightly decreased stability over the same time scale. Gondo et al., (2024) also found that fruit drinks remained stable for at least one year when stored at -20˚ C compared to -80˚ C. ”

In addition, there is no mention to how the samples were normalized for metabolomics and this could be a very important factor. Sampling using a hammer and chisel will provide an inconsistent amount of material per sample and thus an inconsistent extraction for each. Is it possible that coral reefs in areas with anthropogenic impact are easy to chisel and thus provided more material? If so, this would offset the overall results. With a methanol extract, it might be possible to pellet the protein and resolubilize that pellet to get an overall protein amount. Samples could then be normalized to protein content unless that protein content is significantly different between the sites.

During collection, each feature was standardized to a surface area of 2 cm^2 (line 288) and then analytically standardized to sample total ion chromatogram (TIC) intensity. In the manuscript we refer to this data standardization as “relative abundance”. We have revised the sentences on line 288 and 399 to more clearly state these methods: “Coral samples were collected from visually healthy colonies using a hammer and chisel following methods described by Greene et al. with a section of skeleton and tissue 2 cm^2…” and “To compare compositional differences between samples, metabolite XIC values were relativized to relative abundance of that metabolite within its sample by dividing the metabolite XIC value by its sample sum XIC (also referred to as total ion chromatogram or TIC).”. This data relativization was done for two reasons: 1) our hypotheses are fundamentally compositional requiring the data to be proportional abundance of metabolites within coral tissues and 2) relative abundances are directly related to actual concentrations in that by relativizing to TIC we control for variance in tissue thickness, despite standardization during sample collection (to 2 cm^2).This data relativization is very common within the field of microbial ecology (specifically 16S amplicon sequencing) as the total amount of biomass can be hard to accurately assess – especially when the samples are taken from water. Finally, relative abundance transformations help to correct for differences in ionization between ion features as the peak areas (xic values) are not directly related to the same molecular quantities across different molecules. Again, this is akin to how relative abundances are used within the sequencing field to correct for differences in sequencing affinity.

For the LC-HRMS method, a 200 uL aliquot was provided to Metabolomics Australia. Was this aliquot used without further processing? It is atypical to use methanol as the injection solvent for an LC-HRMS method as it can cause inconsistency during injection. Often this sample would be dried down and reconstituted in a more compatible injection solvent for the reversed phase method used. Can the authors clarify this aspect? Why was negative ion analysis not collected? This will miss many key metabolites.

We agree that we would typically use a different injection solvent. Metabolomics Australia in partnership with our group was working towards developing a robust methodology for environmental samples, with minimal sample preparation, that could be used by non-experts. Using the 100% methanol extract instead of our more typical 80:20 MeOH:Water + 0.1% formic acid mixture did likely reduce our analytical window. However, as evidenced by the results summarized within this manuscript we still retained a broad range of metabolites that we would expect from corals such as betaine lipids.

Only analyzing in positive ion mode, came down to time and money limitations where we could only run the samples in one mode because of the sheer number of samples combined with having to pay for them through a private company. When we are only able to run a single ion mode, we use positive ion mode for two reasons: 1) The grand majority of the libraries which we use are run in positive mode and so the ability to label our spectra would be greatly reduced if we only ran in negative ion mode 2) All of our other datasets and the majority of the datasets available on massive are run in positive mode. Running in negative ion mode would reduce our ability to do repository scale analyses in the future where we compare other datasets to this one.

The methanolic extracts were spiked with 2-aminoantracene, but the authors do not state how much was spiked in and what concentration was spiked. In addition, the authors should expand as to why this was added.

Thank you for highlighting the need to specify why this was added. Now on line 288 we have added: "..with a section of skeleton and tissue 2 cm^2 placed in 15 ml of HPLC grade methanol (Sigma Aldrich) 5.17 $\mu mol\ L^{-1}$ 2-aminoanthracene (Sigma Aldrich) on ice for metabolite extraction and transferred to a freezer as soon as possible at -20° C[48]. 2-aminoanthacene was dissolved into stock HPLC grade methanol prior to aliquoting into glass scintillation vials for later use as a quality control indicator."

Figure 4 is very difficult to read and require increase to size to 200% in order to review and evaluate it. Reviewing panel B, the authors have identified both PCs and PAFs (and lyso versions). However, the authors are likely aware that these

are isomeric and thus would have to be separated via LC in order to distinguish them from each other. Did the authors verify if separation was achieved to distinguish them? Note that a simple searching using GNPS or other database would identify them because MS/MS is identical. They also list generic names for Lyso-PAF and Lyso-PC and PC. These should be listed as the specific species that was measured instead of the overall class since in this section, they are referring to specific metabolites.

Thank you for pointing this out. It is clear from this comment that in trying to simplify/summarize our data for non-chemist audiences (and to save space within the figure) we have both oversimplified the chemical data too much and not allowed for enough space for the presentation of the actual trends we are seeing. Because of this comment and a few from Reviewer 3 we have decided to bring Table S1 into the main text and reduce figure 4 down to just the original left-most panel:

Figure 4:

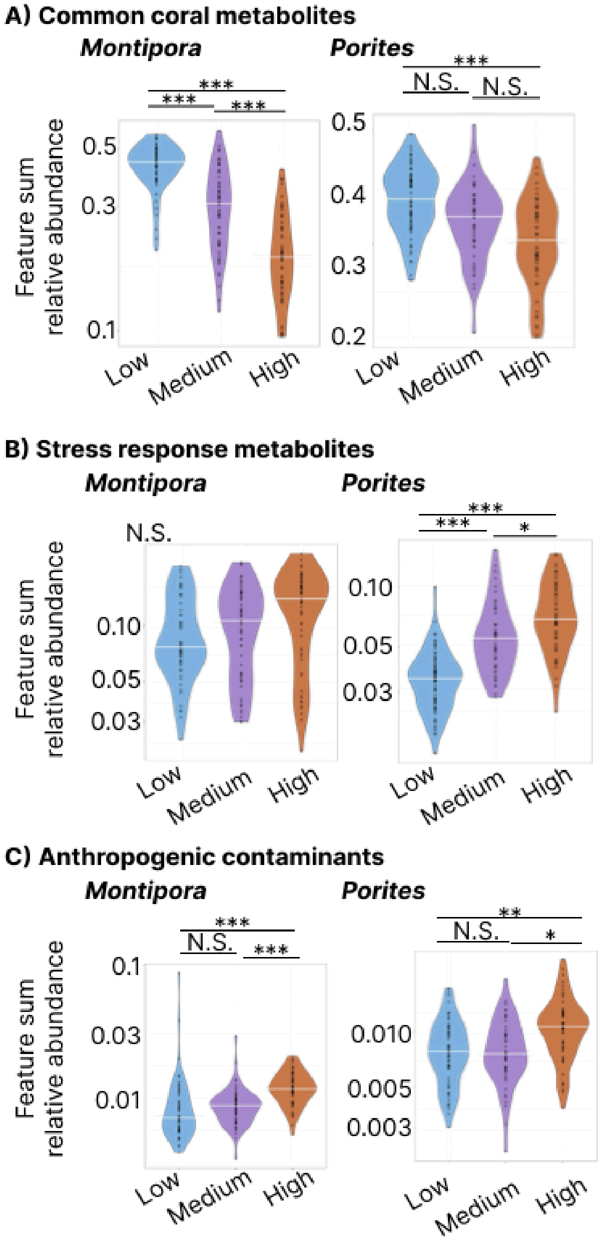

Table 1. Site enriched features spectral library matches

Compound name from spectral libraries	Cosine score	Source
L-Tyrosine	0.9726	Coral holobiont metabolite
DL-PHENYLALANINE	0.9993	Coral holobiont metabolite
BETAINE	0.9479	Coral holobiont metabolite
betaine	0.916	Coral holobiont metabolite
DGCC (28:7)	0.7756	Coral holobiont metabolite
DGCC (36:5)	0.9987	Coral holobiont metabolite
DGCC (38:6)	0.9967	Coral holobiont metabolite
Lyso DGCC (14:0)	0.9708	Coral holobiont metabolite
Lyso DGCC (16:0)	0.9781	Coral holobiont metabolite
Lyso DGCC (18:5)	0.9683	Coral holobiont metabolite
Lyso DGCC (22:6)	0.9834	Coral holobiont metabolite
Stachydrine (L-proline betaine)	0.996	Coral holobiont metabolite
(-)-L-Carnitine	0.7673	Coral holobiont metabolite
Acetylcarnitine	0.9979	Coral holobiont metabolite
Butyrylcarnitine	0.9981	Coral holobiont metabolite
Isovalerylcarnitine	0.9988	Coral holobiont metabolite
Tetradecanoyl-L-Carnitine	0.8965	Coral holobiont metabolite
Stearoyl-L-Carnitine	0.7322	Coral holobiont metabolite
Palmitoylcarnitine	0.991	Coral holobiont metabolite
PROPIONYLCARNITINE	0.9976	Coral holobiont metabolite
acetylcholine	0.9747	Coral holobiont metabolite
PE(20:4/0:0	0.9926	Coral holobiont metabolite
PE(22:4/0:0)	0.9942	Coral holobiont metabolite
DGTS 16:0	0.9589	Coral holobiont metabolite
DGTS 18:2	0.935	Coral holobiont metabolite
DGTS 20:3	0.9385	Coral holobiont metabolite
L-Tryptophan	0.9998	Coral holobiont metabolite
Dimethylsulfoniopropionate (DMSP)	0.9807	Coral holobiont stress response
Homarine	0.9981	Coral holobiont metabolite
Palythene	0.8455	Coral holobiont metabolite
Palythine	0.8792	Coral holobiont metabolite
8-HETE	0.8866	Coral holobiont metabolite
cis-7101316-Docosatetraenoic acid	0.758	Coral holobiont metabolite
trimethylammoniumpropionate (TMAP)	0.9874	Coral holobiont metabolite
PC(0:0/16:0)	0.9954	Coral holobiont stress response
PC(0:0/18:1)	0.9991	Coral holobiont stress response
PC(0:0/20:4)	0.9964	Coral holobiont stress response
PC(18:2/0:0)	0.9992	Coral holobiont stress response
PC(18:3/0:0)	0.9989	Coral holobiont stress response
PC(20:2/0:0)	0.9963	Coral holobiont stress response
PC(20:3/0:0)	0.996	Coral holobiont stress response
PC(20:5/0:0)	0.9953	Coral holobiont stress response
PC(22:4/0:0)	0.9978	Coral holobiont stress response
PC(22:6/0:0)	0.996	Coral holobiont stress response
PC(O-18:0/20:4)	0.9992	Coral holobiont stress response
PC(O-18:1/0:0)	0.9638	Coral holobiont stress response
PC(O-18:1/20:4)	0.9994	Coral holobiont stress response
PC(P-16:0/20:3)	0.9995	Coral holobiont stress response
1-(1Z-Hexadecenyl)-sn-glycero-3-phosphocholine	0.9921	Coral holobiont stress response
1-(1Z-Hexadecenyl)-sn-glycero-3-phosphocholine	0.9919	Coral holobiont stress response
1-Hexadecanoyl-sn-glycero-3-phosphocholine	0.9492	Coral holobiont stress response
1-Hexadecyl-sn-glycero-3-phosphocholine	0.9975	Coral holobiont stress response
1-Myristoyl-sn-glycero-3-phosphocholine	0.9853	Coral holobiont stress response
1-Stearoyl-2-hydroxy-sn-glycero-3-phosphocholine	0.9977	Coral holobiont stress response
Lyso-PAF C-18	0.9574	Coral holobiont stress response
Lyso-PC(16:0)	0.9958	Coral holobiont stress response

PAF C-16	0.9976	Coral holobiont stress response
9,10-dihydrojasmonic acid	0.8801	Anthropogenic contaminant
DEET	0.9927	Anthropogenic contaminant
44'-Sulfonylbis[2-(prop-2-en-1-yl)phenol]	0.6332	Anthropogenic contaminant
44'-Sulfonylbis[2-(prop-2-en-1-yl)phenol]	0.5198	Anthropogenic contaminant
Isoprocarb	0.9283	Anthropogenic contaminant
Monolinolenin (9c,12c,15c)	0.8615	Anthropogenic contaminant
2-Naphthylamine	0.9911	Anthropogenic contaminant
Tetradecyldiethanolamine	0.9201	Anthropogenic contaminant
O-4-Methoxybenzoylepilupinine hydrochloride	0.9667	Anthropogenic contaminant
2,4,7,9-Tetramethyl-5-decyne-4,7-diol	0.8645	Anthropogenic contaminant
Benzyldodecyldimethylammonium	0.9688	Anthropogenic contaminant
Benzyltetradecyldimethylammonium	0.8645	Anthropogenic contaminant
Decynediol tetramethyl di(2-hydroxyethyl) ether	0.5794	Anthropogenic contaminant
Glycerol 1-myristate	0.9644	Anthropogenic contaminant
Nandrolone	0.569	Anthropogenic contaminant
Phenethylamine	0.9963	Anthropogenic contaminant
2-Amino-1-phenylethanol	0.9409	Anthropogenic contaminant
7-Hydroxy-3'-methoxyflavone	0.7023	Anthropogenic contaminant
b-Caryophyllene oxide	0.784	Anthropogenic contaminant
Carveol	0.6255	Anthropogenic contaminant
Adenosine	1	Anthropogenic contaminant
Ethoxyquin	0.9377	Anthropogenic contaminant
Hetisine	0.9957	Anthropogenic contaminant
Eicosapentaenoic acid ethyl ester	0.8066	Anthropogenic contaminant
Pinolenic acid	0.8578	Anthropogenic contaminant
Tributyl phosphate	0.9764	Anthropogenic contaminant
Tris(2-butoxyethyl) phosphate	0.6972	Anthropogenic contaminant
Abrine	0.9729	Marine fungi sponges and E. Coli
Lauryldiethanolamine	0.9655	MS microtube contaminant
Pentaethylene glycol	0.9925	MS microtube contaminant
Hexaethylene glycol	0.8836	MS microtube contaminant
Tetraethylene glycol	0.7925	MS microtube contaminant
1-Linoleoylglycerol	0.9305	Plant metabolite
2-(2-hydroxybut-3-en-2-yl)-3a669a-tetramethyl-2455a7899 b-octahydro-1H-benzo[e][1]benzofuran-45-diol	0.9712	Unknown
Bucharaine	0.9757	Unknown
N-[5-(2-methylpropyl)-4-oxo(13-dioxan-5-yl)]benzamide	0.9788	Unknown
dehydrohaliclocyclin F dial	0.9899	Unknown
histamine-C23:1	0.9169	Unknown
NCGC00384783-01_C17H25NO3_Azepino[321-hi]furo[3 2-e]indole-210-dione 8-ethyldodecahydro-11-methyl- (7aR8R8aS11S11aS11bR11cR)-	0.9934	Unknown
1-Hexadecanoyl-sn-glycerol	0.9531	Unknown

**A full table with citations for the source and additional chemical classification information is available as Supplemental Table 2*

Figure 4 panel C, they identified Adenosine, which is an important nucleoside. Due to their extraction conditions, the identification of this could be due to degradation of ATP, ADP and AMP. Also, they label adenosine as pharmaceutical in origin which is incorrect.

We agree that there are multiple sources of adenosine, both internal pools and external inputs, that we cannot untangle. We have further investigated the proportional abundance of adenosine within our data to address the reviewers concern that the adenosine observed within this experiment was from internal pools (ATP/ADP/AMP

degradation), rather than external inputs of pharmaceutical and skin cosmetic applications (Samsel et al., 2013; Szendzielorz and Spiewak, 2025; Cherian et al., 2024). Going into this analysis we hypothesized that the internal pools of adenosine (including adenosine from ATP/ADP/AMP degradation) would be stable and consistent across our corals and sites with some inherent variability that is small in proportion. Below are the results of that short analysis. Because the tissue concentrations increase consistently from low to high anthropogenic influence on the metabolome it is reasonable to conclude that the anthropogenic portion (Samsel et al., 2013; Szendzielorz and Spiewak, 2025; Cherian et al., 2024) of adenosine recovered from the tissues is indeed increasing with land use while the internal pools are likely stable.

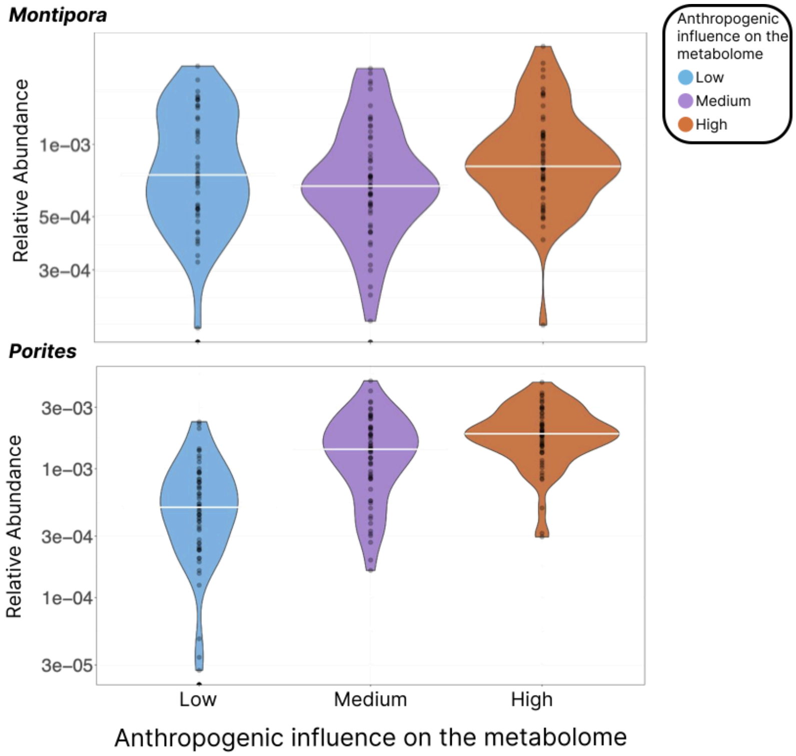

Why were the water samples not used to analyze for agricultural products in addition to the water quality? This seems like a missed opportunity as it could be used to evaluate contamination on the day of collection.

We did not do any solid-phase extractions to analyze the DOM composition. We agree with the reviewer that this is a minor missed opportunity, however there is no guarantee that agricultural or pharmaceutical contamination would have been present or enriched on the day of the surveys as these inclusions are temporally variable and can be largely influenced by increased rain, submarine groundwater discharge, etc. However, that is the power of assessing the bioaccumulation within benthic organismal

tissue; this accumulation can show build up over time in specific sites rather than single time point water concentrations.

Another key concern is whether a classical ANOVA was conducted based on site and metabolite rather than summed metabolite based on subclass? It is unclear as to the overall variability of the sampling approach and methods used as to whether individual metabolites would be identified as significant. This would be important to clarify.

As explained above, we utilized a linear mixed model to elucidate molecular family enrichment between the sites. This does not utilize any sum calculations based on molecular family or subclass. On lines 404 we have added text to better clarify this point: "To identify molecular families that vary between our sampling sites we tested the effect of site number on subnetwork abundance with metabolite number as a random effect using a linear mixed model (subnetwork abundance ~ siteNumber + (1|metabolite)). All metabolites and molecular families presented herein were limited to those that were significantly different between sites as defined from the linear mixed model (false discovery rate corrected LMER p-value < 0.05). "

Reviewer #3

The paper deals with a relevant and emerging topic: the impacts of anthropogenic activities, through the release of chemical pollutants, on corals and coral reef ecosystems. Since the collected dataset and its implications are potentially of great value, I support its publication in a high-quality platform like Nature Communications. However, given the serious shortcomings in the data analysis and interpretation, the manuscript requires substantial revisions before it can be considered for publication. I appreciate the work put into this manuscript by the authors, and hope the provided comments will help them improve the manuscript.

Thank you for your kind words and the considerable effort that went into this review. The suggestions on how to improve this manuscript have proven very helpful in making this manuscript stronger and more easily interpretable to wider audiences. Below you will find a point-by-point response to each critique or question which was brought about in the first round of reviews.

General comments

1) l. 36-37. "…the bioaccumulation of pollutants in corals impacts all but the most remote reef ecosystems." Did the study actually demonstrate the impact of the

accumulated chemicals on coral health? Although this relationship is likely, the study only showed a positive relationship between the biotic concentrations of anthropogenic chemicals and the intensity of human land use, rather than providing proof of a causal relationship between those concentrations and reduced coral health, for example, through toxicity.

The reviewer has raised a valid point. As this was just a survey we did not directly test how accumulation of anthropogenic contaminants and changes in metabolome chemodiversity reduce health of reefs but we do show a correlation between these metrics and overall benthic coral cover on reefs where we were able to recover temporal coral cover data from. As such we have modified the lines on 36-37 to better reflect this correlative relationship "Our results demonstrate that the bioaccumulation of pollutants and compositional changes in the metabolome of corals imposed by anthropogenic activities is correlated with coral benthic cover at specific reef sites. Together, our findings suggest a relationship between coral health, accumulation of dangerous contaminants within corals, and anthropogenic disturbance that is consistent across species."

2) l. 37. "…which will disproportionately threaten communities reliant on reefs." Do the authors mean biological communities, human communities, or both? And why disproportionally? If anything, I would say the reef-dependent communities are threatened proportionally to the level of anthropogenic activities nearby. And did the authors compare the impacts of the bioaccumulation of chemicals between reef-dependent and independent communities?

We thank the reviewer for identifying this point of confusion. This line was specifically about human communities that rely on reefs, such as those that rely on subsistence fisheries. However, due to space requirements, we have decided that this line of thought will not be sufficiently explained and may lead readers astray in an abstract. We have removed this phrase and left this topic to be discussed further in the discussion section.

3) The metabolite clusters were "classified as less, moderately or highly influenced by human activities based on the water quality and the onshore habitat disturbance." The metabolic clusters were defined by hierarchical clustering of the relative abundances of nontarget features. I have two concerns regarding this approach:

a) To do this, the relative abundances of metabolites within samples were used. However, these relative abundances are not directly related to actual concentrations in the coral tissues, since nontargeted analyses are at best semi-quantitative. Moreover, since they are relative within samples, they may not be comparable between samples. Additionally, they were grouped into

subnetworks/single-loop nodes, making a quantitative comparison and thus grouping based on metabolite abundances problematic, since we don't know what the composition and relative and absolute abundances of the individual features within these subnetworks were, and whether they were comparable between samples. I understand the authors are looking for an approach to make untargeted metabolomics data manageable and interpretable, but I worry that this approach may oversimplify the complexity of the situation in the field. I am curious how the authors see this, and whether they can come up with a more quantitative way of comparing their samples in terms of chemical diversity. An approach visualizing the overlap in individual features between samples, for example, using a Venn diagram, may better represent actual differences between sites. Additionally, a multivariate analytical tool such as NMDS and subsequent analysis of similarity may help group sites based on the actual composition of metabolites. Multilevel pattern analysis may then identify particular (groups of) features associated with those site clusters.

There was some confusion from Reviewer 1 on the topic of the hierarchical clustering and the sum calculation within subclass chemical classification. This was done largely to help readers more easily assess compositional variability between each cluster identified. However, as R1 pointed out this was hard to read and was confusing as it suggested a different statistic was used to identify these differentially abundant metabolites. As such we have reverted to including the total abundance of the molecular family within this plot. This removes the *in silico* predicted chemical classifications, however the figure is now much more readable and is in line with the statistic which was performed: a linear mixed model of the molecular family abundance with feature number as a random effect. We have additionally performed a principal component analysis on different levels of relative abundance grouping (feature, subnetwork, *in silico* predicted annotations) to demonstrate how robust the clustering of sites was (Supplemental Figure 2).

To your point about untargeted metabolomics being at best semi-quantitative, we agree. And this is actually the reason that we have incorporated relative abundance transformations to better evaluate our hypotheses. Our hypotheses are fundamentally compositional requiring the data to be proportional abundance of metabolites within coral tissues. Relative abundances are directly related to actual concentrations in that by relativizing to TIC we control for variance in tissue thickness, despite standardization during sample collection (2 cm^2). This data relativization is very common within the field of microbial ecology (specifically 16S amplicon sequencing) as the total amount of biomass can be hard to accurately assess – especially when the samples are taken from water; by relativizing to total peak area (TIC) we are ensuring that we can compare metabolome composition between samples without risking bias as the result of differences between ionization that can be found between ion features. This is akin to

how relative abundances are used within the sequencing field to correct for differences in sequencing affinity. Throughout the manuscript we had made sure to carefully word all phrases about metabolites as the abundance within samples: e.g. “Compared with the low metabolome disturbance site cluster, common coral metabolites in high disturbance areas had lower relative abundance by 50.9% and 16.7% in Montipora and Porites, respectively”. However, to make sure this nuance is understood by a broad range of readers we have added the following sentence to the methods on line 403: “To compare compositional differences between samples, metabolite XIC values were relativized to relative abundance of that metabolite within its sample by dividing the metabolite XIC value by its sample sum XIC (also referred to as total ion chromatogram or TIC). It should be noted that the XIC values and relative abundances within each sample are not quantitative (e.g. not equivalent to µM concentrations) and all comparisons in this manuscript are comparing the percent of total ion intensity within each sample to that of another sample.”

The suggestion to add an NMDS or equivalent plot was a great idea. It does a very good job of demonstrating the clustering in a more intuitive way and also demonstrates how robust the clustering of sites is regardless of the type of summation. Please see our new supplemental figure 2 and modifications to Figure 1.

The grouping of ion features into their respective subnetworks for statistical analysis has been an approach which our group has been using for a number of years (see Quinlan et al., 2023 and Quinlan et al., 2025). In this and our previous work we have used a linear mixed model to predict subnetwork abundance and enrichment with feature number as a random effect (subnetwork abundance ~ siteNumber + (1|metabolite)). This treats the molecular subnetwork as a molecular family of structurally similar ion features (see Nguyen et al., 2013). This approach helps to identify molecular families enrichment more readily as it is less biased by 0’s and noise of individual features (in individual samples) within the molecular family. The summing approach within the first figure is mostly in an effort to better summarize within visuals how molecular families shift between samples. It is also common within the untargeted MS field as molecular families are structurally similar with likely minimal differences in ionization affinity. Whether we take the ion feature abundance, molecular family abundance or *in silico* predicted annotations, we still observe the same robust clustering. We have added this comparison into our new Supplemental Figure 2 and added following text to lines 82: “Principal coordinate analysis (Figure 1A) and hierarchical clustering (Figure 1B; Supplemental Figure 1) of sites by mean molecular family relative abundance in coral tissue samples demonstrated consistent metabolome groupings in both coral species (Supplemental Figure 2) corresponding with land use disturbance levels (n = 10 to 15 corals per site).”

b) It seems that the qualification of the clusters as low, medium, or high anthropogenic metabolome influence was done on a purely qualitative basis. Although that classification may seem intuitive, the values for water quality and the onshore habitat disturbance represented in the heat map in Fig. 1 do not seem to allow such classifications. For example, there are locations in clusters 1 and 2 that have a relatively high level of watershed disturbance. Moreover, the water quality parameters are also not uniformly distinct for each of the clusters, with relatively high and low values distributed over the clusters. Is it correct that these classifications were performed by the authors and were not quantitatively determined based on the numerical values of the parameters? In that case, I strongly suggest refraining from such a bias-prone approach and applying a continuous numerical disturbance score based on measured values.

I believe this was a misunderstanding in what we meant by “anthropogenic metabolome influence” due to the phrasing within the main text and legends. We are naming each cluster based on how much the metabolome is influenced by anthropogenic activity. This cluster naming comes from a few distinct findings: 1) the combination of ammonium, watershed disturbance, phosphate and silicate explained greater than 50% of the chemodiversity variance, and between 37% and 50% of the variance in N content. 2) Anthropogenic metabolites have a higher relative abundance in cluster 3 compared to 1 and 2 (Figure 4). 3) What we annotated as “common coral metabolites” have the highest relative abundance in cluster 1 compared to cluster 3 (Figure 4). In concert these data suggest that the metabolome chemodiversity is influenced <u>more</u> in the third cluster than in cluster 1 and specifically by anthropogenic activities/nutrient dynamics. In writing this manuscript we wished to avoid the misunderstanding which you have brought up here. We have edited sentence on line 114 to now read: “The close correspondence of watershed disturbance, water quality metrics and cluster patterns in metabolite composition allowed us to assign sites into three groups based on the amount of influence that anthropogenic activities/nutrients had on the metabolome chemodiversity. These groups ranged from low (cluster group 1), medium (cluster group 2) and to high (cluster group 3) levels of metabolome disturbance (Figure 2)”. Further, we have modified every legend to read “Anthropogenic influence on the metabolome”.

4) The extraction of metabolites from the coral tissue used solely methanol as the extraction solvent. However, given the focus of the study on metabolites in tissue, a more nonpolar extraction solvent may have improved the extraction of the lipophilic chemicals that generally accumulate more strongly in biota. Why did the authors choose to extract only in methanol? Are any data available on the extraction efficiencies of this method?

Overall within the coral untargeted metabolomics field this extraction solvent is relatively common (Quinlan et al., 2023; Pei et al., 2022; Hartman et al., 2017; Gamba et al., 2022). The original collection team was specifically following a protocol designed for non-specialists (Greene et al., 2020). This protocol was based on previous work that has been done on coral metabolomes and continues to be used in the field to capture a wider range of metabolites (Roach et al., 2023; Berg et al., 2025). Further, Charriez et al., (2020) have previously shown the efficient extraction of lipids using a MeOH extract when compared against Bu:MeOH (3:1) mixture. There is of course always a chance we are excluding some lipophilic metabolites using this method, however going to a more nonpolar extraction solvent could reduce the visibility of other molecules of interest. Further, when comparing to other datasets that use MeOH or a MeOH:water extraction solvent, we could miss metabolites that were outside of our analytical window.

5) The authors use the nitrogen/phosphorus content to draw conclusions on the nutrient availability in the field, and the nominal oxidation state of carbon to derive the "energetic availability of the metabolome." What is the energetic availability? And can such conclusions be drawn from these data? Is previous research available that demonstrates these correlations? Moreover, these values were, again, based on the relative abundance of the metabolites, which are not directly related to concentrations. Hence, it seems premature to draw conclusions on nutrient availability and energetic availability from these values.

It is true that we cannot directly relate the nitrogen/phosphorus content and nominal oxidation state of carbon (NOSC) to concentrations and are not trying to make any claims that a specific nitrogen or phosphorus content (based on our values) is a set concentration. However, what we are doing within this manuscript is comparing relative nitrogen/phosphorus content and NOSC between corals, and sites. For example one conclusion we are drawing herein is that the nitrogen content of the corals in the lower metabolome influenced sites was higher than those in the higher metabolome influenced sites. The relationship between the nominal oxidation state of carbon and energetic availability comes from LaRowe and Van Cappellen (2011) and the organic matter field (see application of this empirical relationship in Wegley Kelly et al., 2022). LaRowe and Van Cappellen showed an empirical relationship between the Standard Gibbs energies of the oxidation half reactions ($\Delta G_{C\ OX}$) of various organic compounds to the NOSC at 25° C and 1 bar with a correlation coefficient R of 0.94. Following LaRowe and Van Cappellen's work, a compound with a lower NOSC has more potential energy, however that energy may be harder to obtain due to the stability of the bonds within the molecule (Gunina, and Kuzyakov 2022).

6) L. 153-154 & 164-168. Tukey's test is not a correlation analysis. It is a post-hoc test after an ANOVA that can indicate significant differences between treatments. A correlation analysis would require a continuous numerical value for the disturbance level and a different statistical test. See also my general comment 3b.

We thank the reviewer for catching this! Both of the sentences highlighted were originally reporting two separate findings that were conflated during the editorial process 1) The Tukey comparison using the *emmeans* function significance between the anthropogenic metabolome influence clusters and 2) The relationship between the environmental parameters ammonium, phosphate, silicate and habitat disturbance and the shannon diversity/nitrogen/phosphorus/NOSC using a linear model. We have corrected these sentences and they now read:

Line 162 - "In both coral species, Shannon diversity increased, and nitrogen content decreased in the sites with more anthropogenically influenced metabolomes (FDR-corrected estimate marginal means comparison p-value < 0.001; Fig 3). In both corals, benthic phosphorus, benthic ammonium, benthic silicate and onshore ecosystem disturbance explained 43.53% and 44.68% of the variance in Shannon diversity (Montipora: multiple linear regression p-value < 0.001; Porites: multiple linear regression p-value < 0.001; Table S1). In both corals, benthic phosphorus, benthic ammonium, benthic silicate and onshore ecosystem disturbance explained 28.97% and 36.49% of variance in nitrogen content (Montipora: multiple linear regression p-value < 0.001; Porites: multiple linear regression p-value < 0.001; Table S1)"

Line 164 - "In Montipora, NOSC and phosphorus content were not significantly related to the amount of anthropogenic influence on the metabolomes (FDR-corrected estimate marginal means comparison p-values > 0.05; Figure 3B and 3C). Benthic phosphorus, benthic ammonium, and benthic silicate explained 7.63% of the variance in NOSC (multiple linear regression p-value = 0.0034; Table S1). In Porites, NOSC was significantly lower in more anthropogenically influenced metabolomes, while phosphorus content was higher in more anthropogenically influenced metabolomes (FDR-corrected estimate marginal means comparison p-values < 0.001; Figures 3B and C). In Porites, benthic phosphorus, benthic ammonium, benthic silicate and onshore ecosystem disturbance explained 5.90% in NOSC score (multiple linear regression p-value < 0.001; Table S1) and 30.53% in phosphorus content (multiple linear regression p-value < 0.001; Table S1)."

7) L. 177-178. I understand the argument here, but is this metabolome represented by small organic molecules really the main energy reserve of corals? I assume that would be much better represented by larger lipids and complex DOM molecules, and labile energy sources such as glucose and ATP. I doubt

whether these molecules are adequately preserved and extracted using the applied methanol protocol, and they also do not seem amenable to the applied LC-MS method.

It is true that glucose and ATP are outside of our analytical window. However, what we are trying to show here is a broader view of the quality of the metabolites which are being incorporated into the metabolome of the corals under these different ecosystem circumstances. Given that the majority of the ion features detected within this study are unknown and only putatively annotated using *in silico* prediction tools (CANOPUS, SIRIUS, etc), the nominal oxidation state of carbon (NOSC) has been shown to be a good metric to summarize metabolome quality at least when used in the assessment of dissolved organic matter and its impact on microbial communities (Wegley kelly et al., 2022). To address your concerns and make it clear we are talking about energy available within the metabolites we are evaluated herein, we have modified the sentence on this line (now 186) to read: "The lower NOSC scores and organoheterocyclic compounds in Porites tissues suggest an increased accumulation of recalcitrant metabolites in the Porites tissues, which overall reduce the availability of energy from metabolites within the coral metabolomes."

8) L. 212-216. I find the drawn conclusions here too strong based on the presented data. Especially the "increased stress responses", based only on the enrichment of DMSP, seem too readily concluded. In Figure 4B, the proportional DMSP enrichment seems rather equally distributed across the clusters, based on the little pie chart. Can a generalized increased stress really be concluded based on these data?

The DMSP is just one example of the metabolites annotated as "stress response metabolites". The full list was originally available within Table S1. Because of this concern, we have moved the majority of this table to the main article. DMSP was originally used in this sentence as an example of a well characterized stress metabolite that is more familiar to a coral researcher audience. However, we do agree with the reviewer that how the sentence was originally written suggested too strong a relationship between DMSP and increased stress. This sentence has been removed and now in the text, when discussing stress response metabolites, we have made sure to specify that we are considering these holistically and not specifically only talking about DMSP.

9) Figure 4. What is the identification level of these chemical features? The authors present these features as fully identified, but no measure is given, for example, as Schymanski scores, of the certainty of these identifications. If these are only tentative identifications, the conclusions may certainly be too strong (see general comment 8).

These features have been matched to library spectra based on a cosine score > 0.7 and a minimum of 4 matching peaks. Every mirror plot (observed spectra vs. library spectra) was compared visually along with m/z between the library spectra and the precursor ion. These methods were provided on lines 364-368: "GNPS additionally matched experimental spectra to spectral libraries of previously identified compounds. Features that were matched to known library spectra were manually annotated with their prospective source based on previously identified literature (Supplemental Table 1). Each library match was additionally confirmed based on cosine score (> 0.7), m/z, and mirror match alignment (Supplemental Figure 6)."

10) The discussion contains very few references to the relevant scientific literature. As such, the findings are not properly placed in the context of the current state-of-the-art. For example, in l. 251-252, the authors state that anthropogenic chemicals accumulate with untold physiological consequences. Indeed, too little is known about the toxic effects of anthropogenic chemicals on coral health. However, multiple studies have demonstrated such effects, while the authors did not seem to try to link detected chemicals to potential negative outcomes in corals. The authors are strongly recommended to consult the review paper by Ouedraogo et al. (2023) as well as more recent publications to link detected chemicals with potential ecotoxicological effects in corals. This should also help revise the provided interpretation of the available literature in l. 259-264.

Thank you for providing this and other citations. We were originally referring only to the pollutants detected within this study when we stated that there could be untold impacts. However, we agree with the reviewer that Ouedraogo et al., (2023) is a great citation to include within this discussion as it points to the ecotoxicological effects in corals of many synthetic compounds even if it does not include ones observed within our study. Now on lines 267-275 we have added some extra discussion of this topic that reads: " It is not clear how the contaminants of concerns that we measured in our study impact coral health but targeted studies have demonstrated sediment-vectoring of pollutants to reefs and increased bleaching associated with persistent organic pollutants[11]. As reviewed in both Ouédraogo et al., (2023)[45] and Nalley et al., (2021)[9], numerous pharmaceuticals, hydrocarbons, and pesticides have been shown to impact coral physiology, mortality rates, bleaching susceptibility, gamete fertilization, and settlement. In other systems these contaminants have been demonstrated to cause larval deformation, modify feeding behavior, and increase mortality in zebrafish, daphnia, and mussels even well below accepted environmental concentrations[43,46]. " Additionally we have added more citations throughout the discussion section.

11) L. 280-282. I agree that the presented approach to coral reef impact monitoring is novel. However, monitoring of hydrophobic anthropogenic

chemicals on coral reefs could also be performed using sediment samples, which is much less invasive and does not carry the downsides of working with often endangered coral species. Although it does not provide the same information as biotic sampling, it could be an accessible and low-impact way of monitoring anthropogenic chemical pollution on coral reefs. Moreover, the authors applied chemical analytical methods that are generally more fit for detecting small molecules in the more polar spectrum. These methods seem more applicable when monitoring the water phase rather than biota or sediments. Ideally, water quality assessment on coral reefs would include anthropogenic chemical monitoring of water, sediment, and biota. The authors are recommended to include a reflection on these points, supported by the appropriate scientific literature, in their discussion.

We agree this is an important point to expand upon within our discussion. We have now revised this sentence into it's own paragraph on lines 287-301: "As mixotrophic sessile organisms, corals are especially capable of bioaccumulating contaminants through both the uptake of dissolved contaminants and the biomagnification of contaminants via heterotrophic feeding[50-52]. While this does mean corals are more susceptible at risk to anthropogenic contaminants, our results demonstrate that monitoring sessile reef metabolomes may be a useful environmental survey indicator of anthropogenic ecosystem disturbance. Being sessile, the bioaccumulates within coral tissues serve as a marker of both short term and chronic pollution, giving a less temporally dependent assessment of contaminant exposure compared to the discrete measurements derived from water quality monitoring[53]. Further, contaminants within organismal metabolomes demonstrate the biological uptake and accumulation of contaminants that sediment cores and water samples cannot reveal[54]. Tissue collections, especially of endangered coral species, do have their drawbacks. However, newer techniques such as the 16-guage tissue punch protocol from Berg et al., (2025), minimize the invasiveness of these methods[55]. Future studies should further compare tissue metabolome characterization with sediment and water monitoring strategies to understand the potential for metabolomics to expand the abilities of reef monitoring and management."

12) Were seawater blanks for each site included in the chemical analyses? Detected metabolomes should be corrected for the local seawater background to allow conclusions on the bioaccumulation of anthropogenic chemicals. If this was not done, how can the authors be sure that the detected anthropogenic chemicals were accumulated in the coral tissue, rather than simply being present in the surrounding water?

Seawater blanks were not taken for this study and unfortunately the comparison of seawater DOM to tissue levels would not be advisable as the concentration levels are

significantly different. For DOM, to be extracted and analyzed using untargeted metabolomics, we must conduct a solid phase extraction using a resin (PPL, HLB, C18, etc.) and then desalinated. Without the desalination process the DOM does not get separated on the LC very well and ionized correctly during the ESI-MS. Typically what we see in a DOM extraction is what we refer to as the “DOM-hump” (see the image below from Petras and Koester 2017). This occurs while the concentration of ACN is increasing in the LC gradient (between minutes 2-9 in the image below). If we were somehow getting background water extracting in our methanol extract we would expect to see this “DOM-hump” in our samples and is easy to scan for as part of our normal QC processing.

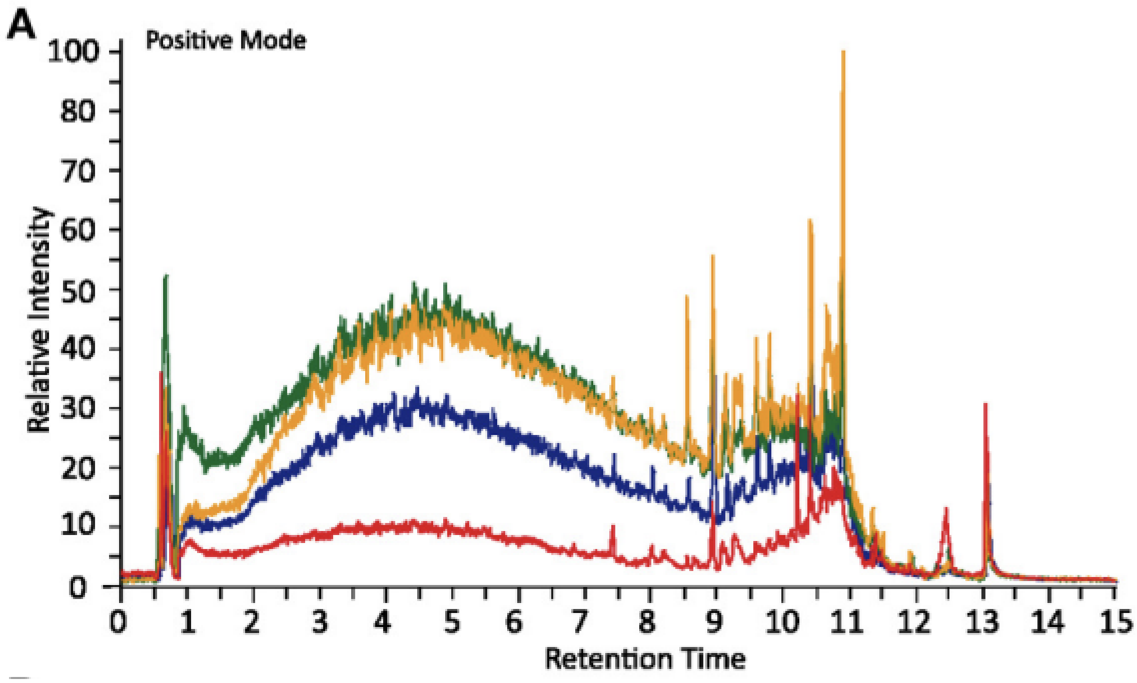

Specific comments

L. 1. The word “metabolism” in the title suggests that the authors studied the metabolism of the corals. Although the paper focuses on the metabolome, I would argue that they did not measure the metabolism of the studied corals.

We have changed the title to: “Coral metabolome quality and contaminant loads track human land use”

L. 48. “…poses a substantial threat…”

Changed as suggested.

l. 49-50. Why particularly CECs? I would argue that exposure to mixtures of all pollutants, regardless of whether they are legacy or emerging contaminants, is potentially harmful to corals. The cited references mostly focus on legacy pollutants, highlighting a knowledge gap on the impacts of CECs on coral health.

We agree that it is all pollutants which pose a threat to coral health. We have rephrased this sentence to better reflect what we were intending in this sentence: “Chronic exposure to both known pollutants, and contaminants of emerging concern (CECs), threatens coral physiology, behavior, reproduction, and microbial dynamics”

l. 56-57. This is a repetition of l. 50-52. I suggest removing the first mention because it will result in a better line of argument while maintaining the specific mention of water quality as a driver of coral reef health.

Thank you, this suggestion definitely helps those two paragraphs flow better.

l. 66. “was” = were.

Changed as suggested.

l. 66. Remove “directly”.

removed.

l. 69-70. “…were additionally accumulating…” = additionally accumulated.

Changed as suggested.

l. 72-75. Suggest changing to: These results suggest that corals use a portion of their energy reserves to respond to the stress levels imposed by local human activities, such as land-derived pollution, reducing their resilience by depleting the ability of these corals to respond to global stressors, such as increased sea surface temperature.

We have changed the sentence as suggested.

l. 82. “…features respectively…”.

Changed as suggested.

l. 88. “through” = of

Changed as suggested.

l. 89. Not only agricultural nutrient pollution, but also pesticides, especially in light of the manuscript’s focus on anthropogenic chemical pollution.

Yes, indeed. We have changed this sentence to read: “...agricultural pesticide and nutrient pollution…”

l. 88-91. Most anthropogenic contaminants find their way to the reefs through runoff, alongside other pollutants from tire wear and other road uses, untreated wastewater, and pesticide use, but this is not specifically mentioned, while subsurface discharge is mentioned. Please add this.

This sentence reads: “For example, anthropogenic impacts to coastal waters includes disturbance induced by coastal development[19], runoff of agricultural pesticide and nutrient pollution[10,20,21], heavy tourism[22], and the introduction of wastewater effluent and septage onto coral reefs via submarine groundwater discharge[23,24].”

L. 103-104. How were they correlated? Was this statistically tested?

Yes, originally cited this model one sentence later but it should have been more addressed here as well. This sentence now reads: “Watershed disturbance and nutrient concentrations were correlated to variation in metabolome chemodiversity (linear model p-value ≤ 0.017; Figure 1b). ”

l. 105. “…wherein the proportional” – such grammatical errors of leaving out the definite article occur throughout the manuscript. The authors are advised to do a final grammatical check of the whole manuscript.

We have reviewed the whole manuscript for grammatical errors and fixed them.

l. 107. “greater” = more

Changed as suggested.

l. 117. “experienced” = were part of

Changed as suggested.

l. 120. “from” = observed at

Changed as suggested.

l. 120. However, there is more information in these trends. For example, how do the authors explain that, according to their classification, the most heavily impacted monitored site was the only site that showed an increase in coral cover over the years preceding the bleaching?

The reviewer raises a very fair point that should be addressed within the manuscript so that readers understand the context herein. Now on lines 122-132 we have added the section “Prior to the 2015 bleaching event the only site where coral cover significantly increased was the site that had the most metabolome influence (Site 3: Kahekili). This increase can be largely attributed to the establishment of the Kahekili Herbivore Fisheries Management Area which sought to increase herbivorous fishes and contend with algal overgrowth at this location[30]. Since 2015, the steepest decline in coral cover was observed at the highly anthropogenically impacted Site 3 (Kahekili), followed by the moderately anthropogenically influenced Sites 1 (Honolua) and 11 (Māʻalaea) while the least influenced Site 15 (Molikini) and moderately influenced Site 8 (Olowalu) did not change significantly with time since 2015 (figure 2). In the case of Site

3, the Kahekili Herbivore Fisheries Management Area is still in effect, so the post-bleaching decline is in spite of these management efforts."

l. 126-127. Remove "…along with…"

Removed

l. 147-148. How is the diversity of metabolites indicative of metabolome energy reserves?

We have changed this sentence to read: "To assess broad-scale shifts in metabolome composition and energy reserves:"

l. 156 & 168. From which analyses are these explained variances derived?

In accordance with prior comments this section has been edited significantly and now reads: "In both corals, benthic phosphorus, benthic ammonium, benthic silicate and onshore ecosystem disturbance explained 43.53% and 44.68% of the variance in Shannon diversity (Montipora: multiple linear regression p-value < 0.001; Porites: multiple linear regression p-value < 0.001; Table S1). In both corals, benthic phosphorus, benthic ammonium, benthic silicate and onshore ecosystem disturbance explained 28.97% and 36.49% of variance in nitrogen content (Montipora: multiple linear regression p-value < 0.001; Porites: multiple linear regression p-value < 0.001; Table S1). These results illustrate an increased metabolome diversity with reduced nutrient content of corals in sites with higher anthropogenic metabolome influence."

L. 220. Is it really the reduced energy reserves that drive the susceptibility of these corals to additional stressors? Could it be something else? There seems to be a lot of room for confounding effects of other stressors.

This sentence was posed originally as a hypothesis for an underlying mechanism of resilience that could be explained by our data. However, to make this more clear we have modified this sentence to make it clear we need direct experiments to make this connection "We posit that human activities greatly reduce the resilience of nearby coral communities to additional stressors as the metabolomes of these impacted corals will have reduced energy reserves, although further experiments are needed to identify a direct link between coral resilience and metabolome energy reserves".

l. 226-227. But octocrylene is not listed as an anthropogenic chemical detected in the present study. Are toxic effects of the detected chemicals to coral described in the scientific literature? The review by Ouedraogo et al. (2023) may provide insights here.

In this sentence, Octocrylene was used as an example of an anthropogenic contaminant which can accumulate in coral tissues and shown to have direct impacts on coral health. Unfortunately neither Ouedrago et al., (2023), nor Nalley et al., (2021) described toxic effects of the detected chemicals observed within our study.

Fig. 4C. DEET is a common personal care product rather than an agricultural chemical.

The reviewer is correct. Originally this group was annotated as “agricultural and pesticides”. In figure 4C we no longer have listed examples of the compounds found and have left them in Table 1.

l. 257. Why “known”? Aren’t all the identified features known chemicals?

This is true. Originally this was to suggest there would be many unknown (unidentified) chemicals that are contaminants as well. However, that point was a bit tangential for this section. We have removed “known” from this sentence.

l. 265. Which management practices are currently being applied in the studied area?

On lines 283-288, we have added the section: “Our data suggest existing management practices are insufficient to address the effects of watershed development. This is evident at Site 3 where The Kahekili Herbivore Fisheries Management Area (KHFMA) has prohibited the take of herbivorous fishes and urchins since 2009 and banned spearfishing by SCUBA since 201330. While this was previously a successful management strategy, since the 2015/2016 bleaching event this site has significantly declined in coral cover despite the KHFMA’s continued efforts.”

l. 269. Although novel, anthropogenic chemical pollution is not a previously unrecognized threat to coral reef ecosystems. See, for example, the papers by Ouedraogo et al. (2023), Nalley et al. (2021), and De Jong et al. (2025).

Thank you for suggesting these manuscripts. We have cited them throughout the manuscript where appropriate.

L. 275. I agree that corals are potentially susceptible to bioaccumulation. However, why do the authors think those chemicals would otherwise break down in the environment, and not in corals? Corals have detoxification mechanisms and a rich microbiome, after all. Moreover, some of these chemicals are very persistent and resistant to (microbial) degradation. I would rather argue that the mixotrophic nature of the coral holobiont, alongside their sessile life history, makes them more susceptible to bioaccumulation since they accumulate chemicals through a variety of pathways.

We agree with the reviewer. “break down” was a poor choice of words for this scenario as not all of the compounds found within this study would be remineralized by the water microbial communities. We have modified this sentence with the suggested argument from the reviewer. Line 284 now reads: “As mixotrophic sessile organisms, corals are especially capable of bioaccumulating contaminants through multiple pathways. Being sessile, the bioaccumulates within coral tissues can also serve as a marker of contamination at specific locations that may not be continuous (e.g. acute-contamination, seasonal runoff, etc.).”

L. 394. Porites lobata, not capitata

This has been corrected.

Reviewer #1 (Remarks to the Author):

Overall, the authors did a good job in responding to this reviewers comments. While I am still concerned with samples stored at -20C in methanol, as along as the authors note this as a potential issue in their manuscript, then it is acceptable.

Thank you so much for your time and effort reviewing this manuscript.

Reviewer #3 (Remarks to the Author)

The authors have carefully reviewed the manuscript based on both reviewers' comments and revised sections accordingly. I appreciate the informed discussion that this gives rise to, and believe it has strongly improved the manuscript. It is now more fit for publication, but some minor concerns remain that should be addressed. I have listed those below:

Thank you again for your feedback and great suggestions. We have made each change as you have suggested. Below you will find our line-by-line responses to each minor concern.

- In response to my comment 3a, the authors explain their approach to standardizing the detected feature intensities. This is now sufficiently addressed. However, rather than using the term "relativized", I suggest the authors use the more common and appropriate term "normalized" or "standardized".

We have changed the text to read "standardized" instead of the less common term "relativized".

- I appreciate the explanation given for my comment 3b. However, based on the response, it seemed that the authors indeed assigned sites based on a qualitative measure of anthropogenic influence. My point was that this should be based on quantitative data, because the current approach appears to introduce a bias. Since subsequent data analyses are performed on these groups, the grouping is of great influence on the outcomes of the study, and should be performed purely based on numerical values and statistics. Either I haven't fully understood the quantitative way in which the clusters were assigned, suggesting that this requires better explanation in the manuscript, or this was indeed assigned based on expert opinion. In the latter case, I invite the authors to come up with a fully unbiased and quantitative approach to the clustering.

In accordance with this concern and comments from reviewer #4 we have added statistical tests about the separation of these clusters. On lines 86-89 we have added the sentences: "Importantly, these metabolome clusters were not defined *apriori* but were rather uncovered based on the separation of the sites metabolomic profiles in multidimensional space. For both coral species the metabolome clusters were significantly separated in multidimensional space (PERMANVA $p < 0.001$; PERMANOVA $R^2 = 0.863$ in *Montipora* and 0.714 in *Porites*; pairwise PERMANOVA < 0.01). There was no significant difference in dispersion within each metabolome cluster in either coral species ($p > 0.05$)."

We have added text in the methods on lines 464-469 to accompany these tests: "Significant separation and dispersion of metabolome clusters in multidimensional space was tested using PERMANOVA tests using the adonis2 and betadisper functions from the Vegan R package[66] with pairwise PERMANOVA's subsequently tested using the pairwiseAdonis2 function from pairwiseAdonis R Package [67]. Dispersion and group separation were tested using bray-curits distance matrices and in the case of *Montipora* did not include the outgroup site (site 12)."

- For comment 4, it may be true that MeOH is a common solvent for untargeted metabolomics in corals, and I understand the value of that. However, in the field of biomonitoring of anthropogenic chemicals, it is common to use (mixtures of) solvents that efficiently extract both polar and nonpolar anthropogenic

chemicals from tissue samples. Any extraction method will provide only a certain fraction of the chemical universe, and that is not a problem, especially if the extraction efficiency is reported. Do the authors have any numbers on the extraction efficiencies for anthropogenic pollutants from coral tissue using this method? That would greatly help readers interpret the presented data. For example, if a common pollutant, say sucralose or caffeine, was not detected in the coral tissue, is this because it was not extracted by the selected method or because it was absent from the tissue?

We agree that this is an important context for our readers. Unfortunately, we do not currently have data that evaluates the extraction efficiencies of coral tissues using different solvents. In the main discussion on line 318-323 we have added the sentence: "Additionally, the extraction solvent can drastically shift our analytical window[56-58]. While there have been recent efforts to standardize the extraction methodology for coral metabolomics[58], currently there have been no direct comparisons of coral tissue extraction efficiencies across different solvents. In context with this study this means that there are likely more anthropogenic contaminants and other metabolites that lie outside of our analytical window."

- The expansion of the discussion in response to comment 11 is valuable. However, some grammatical changes to the amended text are advised: (l. 292-293) "While this does mean corals are more susceptible at risk to anthropogenic contaminants…" should be "more susceptible to" OR "more at risk from"; (l.295) "bioaccumulates" should be "the chemicals bioaccumulated in coral tissues…"

We have made the changes as suggested.

- I understand the challenges of high salt content in LC-MS analyses, given in response to comment 12. However, I disagree that taking seawater blanks is not advisable. Any environmental chemistry study should include blanks, ideally, to correct for background contamination. This is especially true for non-target analyses. Even though I understand why the authors did not include them in this study, I strongly suggest they start using seawater blanks, despite the challenges, in future projects. However, the current findings are still of value, and we can leave it at this for the present study.

Thank you for this suggestion. Because of your advice we are currently investigating how effective this type of strategy is within another more recent dataset.

Reviewer #4 (Remarks to the Author):

The dataset collected here is impressive, particularly the multi-species comparison and the attempt to link metabolomic profiles to anthropogenic disturbance. The overall question is clearly relevant. That said, even after revision, I still find the definition and interpretation of the site clusters problematic, and this carries through several of the downstream analyses and ultimately affects how convincing the conclusions are.

Thank you for your nice comments about our manuscript; we greatly appreciate the effort that has gone into this review and believe all your comments have contributed to making this manuscript more impactful and easily understandable. Below we have responded to each of your individual critiques and revised the manuscript accordingly.

A central issue is that the manuscript relies heavily on clusters derived from hierarchical clustering of metabolomic data, yet I was not able to find a formal statistical test showing that these clusters are actually distinct in multivariate space. The additional ordination analyses (PCA/PCoA) help to visualize the structure, but they do not resolve this point. In fact, in Porites in particular, the pattern looks more like a continuum than clearly separated groups, which makes the current interpretation difficult to reconcile with the data.

At a minimum, I think the authors need to test for group separation using PERMANOVA, based on the same distance matrix used for clustering, and report effect sizes such as R2 alongside significance. It would also be important to include a test of dispersion using PERMDISP, since differences in within-group variance can otherwise give a misleading impression of separation. As it stands, the clusters appear to be defined visually rather than statistically.

Thank you for bringing up this point. It is indeed an important point to reinforce how robust these metabolite clusters with statistical tests. On lines 86-89 we have added the sentences: "Importantly, these metabolome clusters were not defined *apriori* but were rather uncovered based on the separation of the sites metabolomic profiles in multidimensional space. For both coral species the metabolome clusters were significantly separated in multidimensional space (PERMANVA $p < 0.001$; PERMANOVA $R^2 = 0.863$ in *Montipora* and 0.714 in *Porites*; pairwise PERMANOVA < 0.01). There was no significant difference in dispersion within each metabolome cluster in either coral species ($p > 0.05$)."

We have added text in the methods on lines 464-469 to accompany these tests: "Significant separation and dispersion of metabolome clusters in multidimensional space was tested using PERMANOVA tests using the adonis2 and betadisper functions from the Vegan R package[66] with pairwise PERMANOVA's subsequently tested using the pairwiseAdonis2 function from pairwiseAdonis R Package [67]. Dispersion and group separation were tested using bray-curits distance matrices and in the case of *Montipora* did not include the outgroup site (site 12)."

Related to this, the interpretation assumes discrete "low", "medium", and "high" impact groups, whereas the ordination suggests that a continuous gradient may underlie the data. It would help to test this explicitly, for example by asking whether metabolomic variation is better explained by cluster membership as a categorical factor or by environmental variables treated as continuous predictors. Looking at correlations between ordination axes and measures such as land-use disturbance or contaminant load would already go a long way toward clarifying this.

We agree that this is a good point that should be addressed. We have added a comparison of PcOA axis 1 vs. cluster membership to the section where we were already comparing environmental factors to PcOA axis 1. This section, on lines 120-133, now reads: "Hierarchical cluster membership explained more of the variability in multidimensional chemidiversity than the environmental disturbance metrics we collected for both *Montipora capitata* and *Porites lobata* (R^2 = 97.17% and 84.84%, respectively; linear model p-value < 0.001). However, the continuous gradients of watershed disturbance, benthic ammonium, and benthic silicate disturbance still explained more than 50% of the chemodiversity variance at each site in both species (linear model p-value ≤ 0.017), with phosphate concentration explaining an additional 6.74% of the multidimensional chemodiversity variance in *Montipora capitata* (Supplemental Figures 2 and 4). This suggests that the metabolome chemodiversity and cluster membership was driven largely by ecosystem impacts. In fact, every site maintained the same cluster membership in both coral species except for Sites 1 and 12 (Supplemental Figures 1,3,5). The close correspondence of watershed disturbance, water quality metrics and cluster patterns in metabolite composition allowed us to assign sites into three groups based on the amount of influence that anthropogenic activities/nutrients had on the metabolome chemodiversity. We categorized these groups based on the data in terms of levels of metabolome disturbance (Figure 2): low (cluster group 1), medium (cluster group 2) and to high (cluster group 3)."

I also found it difficult to fully understand how the clustering was performed. While hierarchical clustering of XIC intensities aggregated at subclass level is mentioned, key parameters are either missing or not clearly stated. In particular, I could not find a clear description of the distance metric used (Bray Curtis? Euclidean?), the linkage method, the preprocessing steps applied to the data, or how the number of clusters was chosen. Given how strongly these choices can influence the outcome, this level of detail is really needed for the analysis to be evaluated properly.

In Figure 1's caption in reference to the construction of the hierarchical clustering we have added: "Clustering was performed using Euclidian distances." Additionally in the methods on lines 480 – 482 we have added: "Hierarchical clustering was performed using Euclidian distances with dendrogram linkages generated using Ward's

minimum variance method.”

Another point that is not entirely clear to me is how the filtering of metabolites affects the downstream analyses. The manuscript states that analyses focus on metabolites that are significantly different between sites. While that is reasonable in principle, it does introduce a potential circularity, since features are selected precisely because they differ between sites, and are then used to interpret differences between clusters derived from the same data. It would therefore be helpful to clarify whether clustering itself was performed on the full dataset or on a filtered subset. As a simple robustness check, repeating the clustering on the full, unfiltered dataset would help to see how stable the structure actually is.

We have produced a new supplemental figure (Supplemental Figure 2; see below) where we included all subnetworks after the initial data cleaning steps (background removal and minimum peak intensities). As you can see from this analysis, the cluster membership did not change whether we filter subnetworks to only those that are significantly different between sites or not. In the manuscript we have added this additional analysis to lines 85-87: “The metabolome clustering was consistent whether dendrograms were built using molecular families that differed between sites or not (Supplemental Figure 3).”

Hierarchical clustering of molecular family abundance of full metabolite database

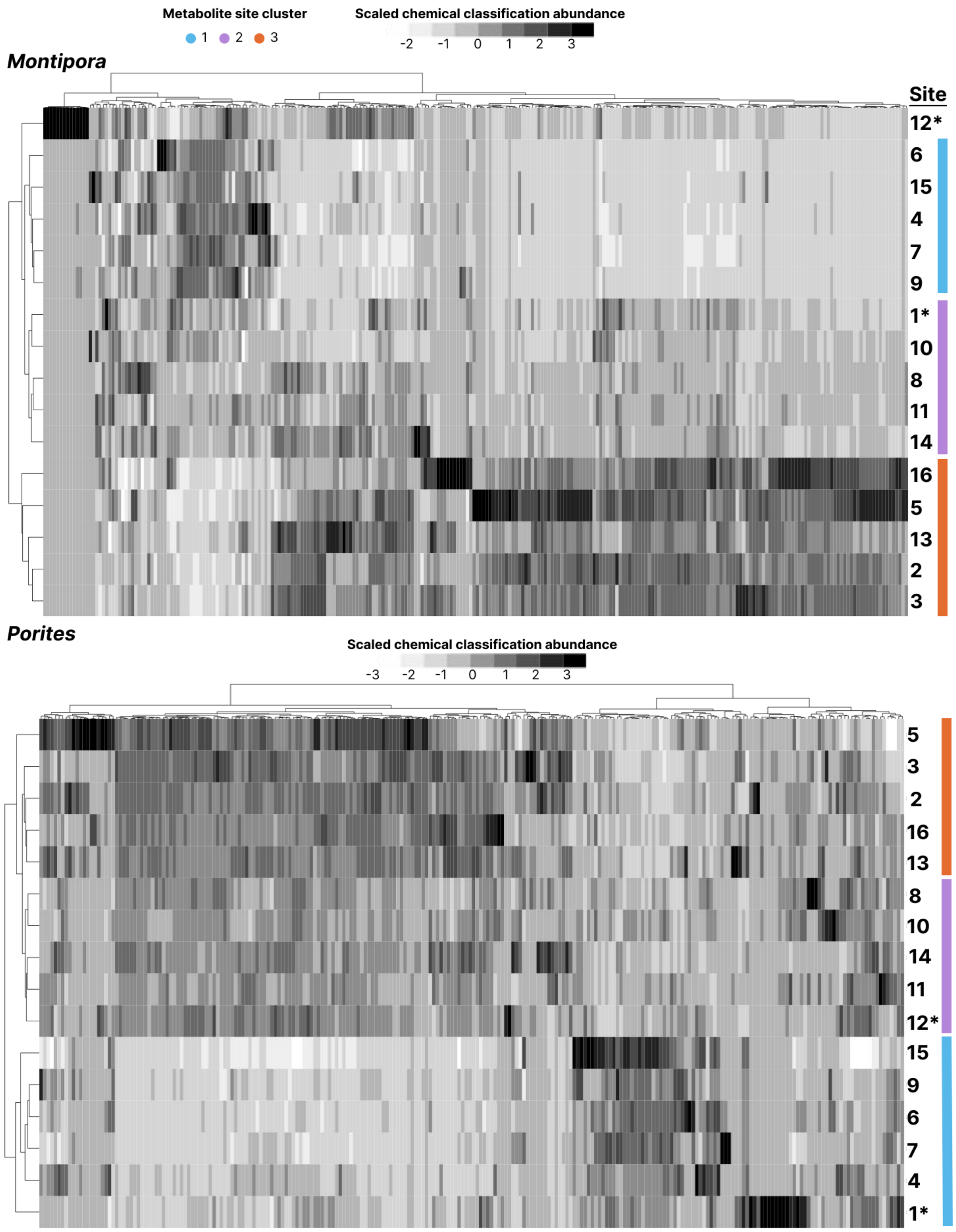

Figure S3. Hierarchical clustering of sites using full metabolome dataset and not only those that differed significantly between sites. Hierarchical clusters were built using metabolite relative abundance summed within molecular families for each sample and averaged for each site. Cells are shaded by z-scored relative abundance of each molecular family. Clustering was performed using Euclidian distances. Dendrogram linkages were generated using Ward's minimum variance method. Asterisks denote sites that changed group classification between each species.

The classification into “low”, “medium”, and “high” impact groups also seems to be derived from the metabolomic clustering and then interpreted in relation to environmental variables. This is fine as an exploratory approach, but it should be stated explicitly. At the moment, it reads somewhat as if these categories were defined independently. It would also strengthen the manuscript to quantify these relationships more formally, rather than relying mainly on descriptive comparisons.

We have added changed the wording throughout the manuscript to make it clearer that we are assigning these cluster designations based off the data directly. See lines 87 – 89: “Importantly, these metabolome clusters were not defined *apriori* but were rather uncovered based on the separation of the sites metabolomic profiles in multidimensional space.”

And lines 131-133: “We categorized these groups based on the data in terms of levels of metabolome disturbance (Figure 2): low (cluster group 1), medium (cluster group 2) and to high (cluster group 3).”

Finally, the fact that some sites cluster differently depending on species is mentioned, but not really explored. This made me wonder to what extent the clustering framework is consistent across taxa, and whether some of the conclusions might depend on species-specific patterns. A more explicit comparison would be useful here.

We agree that this is a valuable line of questioning. We investigated this separation a bit more thoroughly and have added sections to:

The results (lines 129-132): “In *Montipora* tissues there was a single cluster of molecular families that were enriched in site 12. We investigated whether removal of these molecular families in *Montipora* tissues would change the clustering of sites. Indeed, once removed sites 12 and 1 clustered the exact same as in *Porites* tissues (Supplemental Figure 5)”

The methods (line 520): “(see the Supplemental Discussion for a discussion on taxa differences)”.

The supplemental discussion now reads: “The two species evaluated within this study (*Montipora capitata* and *Porites lobata*) are morphologically distinct and vary greatly in life-history and growth strategies (Dubinikas, 2017). Surprisingly the majority of the sites maintained the mostly the same cluster designations within our analysis with the obvious exceptions of Site 1 and Site 12. Site 1 moved from cluster 2 (medium metabolome influence) in *Montipora* samples to cluster 1 in *Porites* (low metabolome). Site 12 grouped with cluster 2 in the *Porites* analyses but was a clear outgroup in *Montipora* samples. The differences in clustering was seemingly because of a single cluster of molecular families enriched in Site 12 evidenced by their removal producing the same clustering of sites in *Montipora* as *Porites* (Supplemental Figure 5). Of the 15 molecular families enriched at Site 12, only 4 had features with library matches. Molecular family 83 had library matches for Lauryldiethanolamine, Tetradecyldiethanolamine, Arachidoyl Ethanolamide, and C17-Sphinganine. Lauryldiethanolamine was the only clear contaminant as it is a well classified MS microtube contaminant. Whereas Tetradecyldiethanolamine was classified as an anthropogenic contaminant that is currently patented for use in industrial settings (Supplemental Table 2), and Arachidoyl Ethanolamide is a pharmaceutically active compound. The source of C17-Sphinganine is unknown. The single-node 145 was classified as the pesticide DEET. The library matches from molecular families 1020, 117 and 46 all had low cosine scores and poor mirror match alignment and thus could not be annotated further. Although these metabolites may be contaminants, because of the high level of uncertainty in their annotations or potential sources, we chose to keep them in our dataset but instead remove Site 12 from the analysis of *Montipora* tissues.”

Additionally, we have added the supplemental figure below:

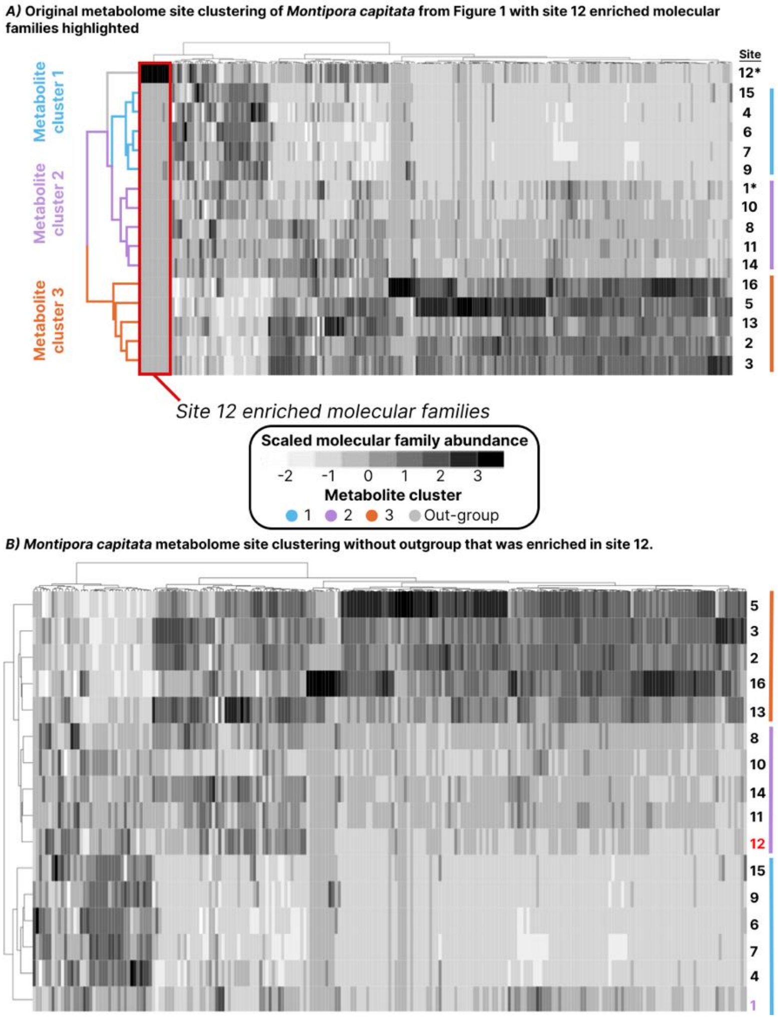

Figure S5. Removal of site 12 enriched molecular families in *Montipora* tissues to investigate clustering differences between *Porites* and *Montipora* tissues. A) Original hierarchical clustering of *Montipora* metabolomes from Figure 1 with site 12 enriched molecular families highlighted with the red rectangle. B) Hierarchical clustering of *Montipora* tissues when the site 12 enriched molecular families were removed from the clustering. Cells are shaded by z-scored relative abundance of each molecular family. Clustering was performed using Euclidian distances. Dendrogram linkages were generated using Ward’s minimum variance method. Asterisks denote sites that changed group classification between each species.

Overall, this is a valuable dataset addressing an important question, and I think the study has real potential. However, the current analysis relies on clusters that are not statistically validated and whose methodological basis is not sufficiently described. Addressing these points would make the conclusions much more robust.

Thank you so much for all the advice. We agree with all of your assessment and believe this has greatly helped the manuscript in both its interpretability and robustness.

Reviewer #4 (Remarks to the Author):

Thank you for the thorough revision. The manuscript is clearly stronger than the previous version, and I appreciate the serious and constructive way the authors responded to the earlier comments.

The additional detail on how the clustering was performed is useful, and the inclusion of the PERMANOVA and PERMDISP analyses addresses an important gap in the original submission. I also found the added robustness check using the full metabolome dataset reassuring, as well as the expanded discussion of the species-specific patterns.

Thank you for the kind words. We greatly appreciate all of your suggestions and agree the article is much stronger with your suggested changes.

My only remaining point is more about interpretation than analysis. Even though the clustering framework is now much better supported, some of the ordination patterns, particularly for Porites, still read to me more as a gradient than sharply separated groups. I would therefore encourage the authors to keep some restraint in describing these categories as fully discrete states, and to note that continuous environmental variation is also likely contributing to the observed structure.

In the supplement where we already had a section comparing the differences between species we have added the lines: "Although the clustering of sites still explains 71.4% of the variation in *Porites* metabolomes (PERMANOVA $p < 0.01$), the cluster dendrogram (Supplemental Figure 3) and overlap of the clusters in multidimensional space (Figure 1B) suggest the pattern underlying changes in *Porites* metabolomes may be continuous rather than discrete. This could explain some of the differences in site membership between the two corals: Site 12 grouped with cluster 2 in the *Porites* analyses but was a clear outgroup in *Montipora* samples…"

Overall, I think the main concerns have been dealt with satisfactorily. This is an interesting and timely study based on a substantial dataset, and in my view the manuscript is now suitable for publication.
